# A decision analysis model for KEGG pathway analysis

**DOI:** 10.1186/s12859-016-1285-1

**Published:** 2016-10-06

**Authors:** Junli Du, Manlin Li, Zhifa Yuan, Mancai Guo, Jiuzhou Song, Xiaozhen Xie, Yulin Chen

**Affiliations:** 1 College of sciences, Northwest A&F University, Yangling, 712100 People’s Republic of China; 2 College of Animal Science and Technology, Northwest A&F University, Yangling, 712100 People’s Republic of China; 3 Department of Animal and Avian Sciences, University of Maryland, College Park, MD 20742 USA

**Keywords:** Pathway analysis, Decision coefficient (DC), Coefficient of determination (CD), Bovine mammary

## Abstract

**Background:**

The knowledge base-driven pathway analysis is becoming the first choice for many investigators, in that it not only can reduce the complexity of functional analysis by grouping thousands of genes into just several hundred pathways, but also can increase the explanatory power for the experiment by identifying active pathways in different conditions. However, current approaches are designed to analyze a biological system assuming that each pathway is independent of the other pathways.

**Results:**

A decision analysis model is developed in this article that accounts for dependence among pathways in time-course experiments and multiple treatments experiments. This model introduces a decision coefficient—a designed index, to identify the most relevant pathways in a given experiment by taking into account not only the direct determination factor of each Kyoto Encyclopedia of Genes and Genomes (KEGG) pathway itself, but also the indirect determination factors from its related pathways. Meanwhile, the direct and indirect determination factors of each pathway are employed to demonstrate the regulation mechanisms among KEGG pathways, and the sign of decision coefficient can be used to preliminarily estimate the impact direction of each KEGG pathway. The simulation study of decision analysis demonstrated the application of decision analysis model for KEGG pathway analysis.

**Conclusions:**

A microarray dataset from bovine mammary tissue over entire lactation cycle was used to further illustrate our strategy. The results showed that the decision analysis model can provide the promising and more biologically meaningful results. Therefore, the decision analysis model is an initial attempt of optimizing pathway analysis methodology.

**Electronic supplementary material:**

The online version of this article (doi:10.1186/s12859-016-1285-1) contains supplementary material, which is available to authorized users.

## Background

To gain more mechanistic insights into the underlying biology of the condition being studied, analyzing high-throughput molecular measurements at the functional level has become more and more appealing [1]. Especially, the knowledge base-driven pathway analysis is becoming the first choice for many investigators, which mainly exploit pathway knowledge in public repositories, such as Gene Ontology (GO) and Kyoto Encyclopedia of Genes and Genomes (KEGG) [2]. KEGG pathway databases store the higher order functional information for systematic analysis of gene functions. Importantly, KEGG pathway databases can be viewed as a set of ortholog group tables including category pathways, subcategory pathways and the secondary pathways, which are often encoded by positionally coupled genes on the chromosome and particularly useful in predicting gene functions [3]. Therefore, KEGG pathway databases are more widely used in current enrichment analysis platforms. There are two advantages in this kind of pathway analysis. One is to reduce the complexity through grouping thousands of differentially expressed genes (DEG) from those high-throughput technologies to just several hundred pathways; another is to increase the explanatory power for the experiment through identifying the most impacted pathways under the given conditions [1, 2].

In the last decade, the pathway analysis has experienced the over-representation approach (ORA) [4–11] and a functional class scoring approach (FCS) stages [12–25]. Both ORA and FCS, including singular enrichment analysis (SEA), gene set enrichment analysis (GSEA) and modular enrichment analysis (MEA), aim to identify the significant pathways by considering the number of genes in a pathway or gene co-expression [2]. However, these methods are currently limited by the fact that they handle each pathway independently [19, 26]. In fact, the pathways can cross and overlap because each gene has multiple functions and can act in more than one pathway [2]. Hence, exploring advanced data analysis methods and considering inter-pathway dependence are still the most important challenge in pathway analysis up to now. To our knowledge, only Go-Bayes method has incorporated the dependence structure of the directed acyclic graph (DAG) in assessing Gene Ontology (GO) term over-representation [27]. Besides, a KEGG-PATH approach took into account the correlation among the KEGG pathways in identifying the most impact pathways and exploiting the regulations among the KEGG pathways [28].

For time-course experiments and multiple treatments experiments, the Dynamic Impact Approach (DIA) had been validated to be an effective functional analysis method in real study based on a priori biological knowledge [29, 30]. In DIA, the impact values and the impact direction were calculated as “Impact *= [Proportion of DEG in the pathway]* × *[average log2 fold change of the DEG]* × *[average of –log P-value of the DEG]*” and “Impact direction = *Impact of up-regulated DEG-Impact of down-regulated DEG*” [29]. In fact, the impact value was a pathway-level statistic aggregated the gene-level statistics for all DEGs in the pathway. By ranking the estimated impact values of pathways and considering the sign of impact direction values, the DIA approach can efficiently identify the most impact pathways and provide the impact direction of pathways. The outstanding advantage of this method is to capture the dynamic nature of the changing transcriptome. But, this ranking method by ‘average values’ had two limitations: 1) handling each pathway independently; 2) considering the effect of transcriptome expression in a cell for each time-course as “equal weight”. In fact, a biology mechanism (or better a biology process) is a very complex network consisting of multiple pathways/functions. Obviously, the mutual regulations among pathways must exist and the effect weight of transcriptome expression at different time-course is unequal. In KEGG-PATH approach, the indirect regulation from the other related pathways was considered in the calculation of total effect for each pathway, neglecting the retro-regulation of this pathway to the other related pathways.

In this study, we attempted to develop a decision analysis model to select the most important pathways from the same KEGG category or subcategory pathways, and to demonstrate the regulation mechanisms among the pathways belonging to the same KEGG category or subcategory. In this method, a decision coefficient (DC) index was conceived by considering the mutual regulation between pathways and was used to identify the most impacted pathways. Besides, the subdivision of DC included not only the direct determination factor of each pathway itself, but also the indirect determination factors from its related pathways. Meanwhile, the mutual regulation mechanisms among pathways can be demonstrated by the subdivision of DC. In addition, the impact direction of each pathway can be preliminarily estimated by using the sign of DC. Moreover, the decision percentage can be obtained by the ratio of DC absolute value of the given subcategory pathway divided by the sum of DC absolute values of all subcategories in the same category. According to the decision percentage, a decision tree can be constructed to visualize the decision results. We tested the utility of the method using the DIA impact value dataset from a functional analysis of the bovine mammary transcriptome during the lactation cycle.

## Methods

To introduce the decision analysis model, we take the KEGG pathways for an example to define the following notations.

We assumed that *X* = (*X*_1_, *X*_2_, ⋯, *X*_*m*_)^*T*^, *X*_*i*_ = (*X*_*i*1_, *X*_*i*2_, ⋯, *X*_*ip*_)^*T*^, (*i* = 1, 2, ⋯, *m*), *X*_*ij*_ = (*X*_*ij*1_, *X*_*ij*2_, ⋯, *X*_*ijk*_)^*T*^, (*i* = 1, 2, ⋯, *m*; *j* = 1, 2, ⋯, *p*) are the sets of KEGG pathway categories, subcategories and the secondary pathways, respectively. Let *y*_*i*_(*i* = 1, 2, ⋯, *m*) be the impact values of the *i-*th KEGG pathway category and *x* = (*x*_*i*1_, *x*_*i*2_, ⋯ *x*_*ip*_)^*T*^ be the impact values of its corresponding subcategory. The vector *x* is assumed to follow a normal distribution *x* ~ *N*(0, *R*_*x*_), where *R*_*x*_ is the correlation matrix of *x*. Based on the path analysis model, the total effect can be subdivided into the direct effect and indirect effect through the equation ${\widehat{R}}_x{b}^{*}={\widehat{R}}_{xy}$, where ${\widehat{R}}_x={\left({r}_{jt}\right)}_{p\times p}$ is the maximum likelihood estimation of correlation matrix *R*_*x*_, and ${\widehat{R}}_{xy}={\left({r}_{jy}\right)}_{p\times 1}$ is the correlation matrix of *x* and *y*_*i*_, b* = (*b*_1_^*^, *b*_2_^*^, ⋯, *b*_p_^*^)^*T*^ is the solved path coefficient indicating the direct effect of subcategory pathways [28]. In fact, the path analysis approach is a standard multiple linear regression model. In the linear regression analysis, the coefficient of determination (CD) (0 ≤ *R* ^2^ ≤ 1) is the proportion of total variation of outcomes explained by the model, which provides a measure of how well observed outcomes are replicated by the model [31]. In other words, the larger the value of CD is, the better the model is. Usually, the CD were defined by the formula ${R}^2=1-\frac{S{S}_{res}}{S{S}_{tot}}$, where *SS*_*res*_ is the residual sum of squares, indicating the discrepancy between the data and an estimation model; *SS*_*tot*_ is the total sum of squares, indicating the total “variability” of data set.

In the path analysis, the CD (*R* ^2^) can be subdivided into the direct CD (${\displaystyle \sum_{j=1}^p{R}_j^2}$) and indirect CD ($$ {\displaystyle \sum_{\begin{array}{l}j=1\\ {}j<t\end{array}}^p{R}_{jt}} $$), which can be denoted with the equation

1 $$ {R}^2={\displaystyle \sum_{j=1}^p{R}_j^2}+{\displaystyle \sum_{\begin{array}{l}j=1\\ {}j<t\end{array}}^p{R}_{jt}}={\displaystyle \sum_{j=1}^p{\left({b}_j^{*}\right)}^2}+{\displaystyle \sum_{\begin{array}{l}j=1\\ {}j<t\end{array}}^p2}{b}_j^{*}{r}_{jt}{b}_t^{*}. $$

The parameter *R* ^2^ characterizes the proportion of total variation of dependent variables *y*_*i*_ determined by all independent variables *x*_*i*1_, *x*_*i*2_, ⋯, *x*_*ip*_. According to the subdivision, the ratios of direct CD and indirect CD in the total CD can be calculated by the formulae

$$ {\displaystyle \sum_{j=1}^p{R}_j^2}/{R}^2,\kern1em \left|{\displaystyle \sum_{\begin{array}{l}j=1\\ {}j<t\end{array}}^p{R}_{jt}}\right|/{R}^2. $$

The comparison result of these two ratios for each pathway category or subcategory will indicate clearly which kind of determination is more important. The fact that the ratio of indirect CD of given pathway was larger indicates that the correlated regulation was more important than the direct determination for this pathway.

**Fig. 1:**
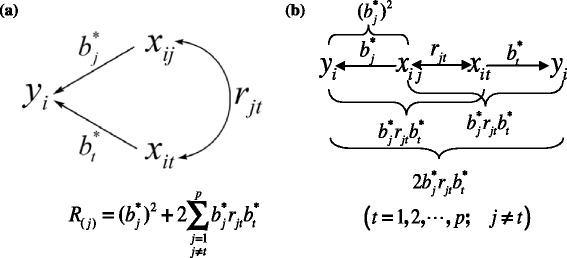
The construction principle of decision coefficient for a specified pathway. **a** One path-chain including one bi-directional arrow. **b** The decision coefficient of one specified pathway including the direct determination factor and the indirect determination factors from all other *p* − 1 pathways

To demonstrate the proportion of total variation of dependent variables *y*_*i*_ determined by a specified pathway *x*_*ij*_(*j* = 1, 2, ⋯, *p*), the decision coefficient (DC) *R*_(*j*)_ of a specified pathway is constructed as the sum of two terms (Fig. 1) based on the ‘coefficient of determination subdivision’ principle of path analysis:

2 $$ {R}_{(j)}={\left({b}_j^{*}\right)}^2+2{\displaystyle \sum_{\begin{array}{l}t=1\\ {}j\ne t\end{array}}^p{b}_j^{*}{r}_{jt}{b}_t^{*}} $$

The first term ((*b*_j_^*^)^2^) is the direct determination factor that demonstrates the direct decision-making capacity of the given pathway. The *b*_j_^*^ value corresponds to the direct effect in the path analysis model. The second term ($$ 2{\displaystyle \sum_{\begin{array}{l}t=1\\ {}j\ne t\end{array}}^p{b}_j^{*}{r}_{jt}{b}_t^{*}} $$) is the indirect determination factor including correlation determinations of the given pathway from all other *p* − 1 pathways. The indirect determination factor shows the indirect decision-making capacity of the given pathway. The phenomenon $$ \left|2{\displaystyle \sum_{\begin{array}{l}t=1\\ {}j\ne t\end{array}}^p{b}_j^{*}{r}_{jt}{b}_t^{*}}\right|>{\left({b}_j^{*}\right)}^2 $$ and $$ 2{\displaystyle \sum_{\begin{array}{l}t=1\\ {}j\ne t\end{array}}^p{b}_j^{*}{r}_{jt}{b}_t^{*}}>0 $$ showed that the correlation regulation will strengthen the decision capacity of the given pathway. On the contrary, $$ \left|2{\displaystyle \sum_{\begin{array}{l}t=1\\ {}j\ne t\end{array}}^p{b}_j^{*}{r}_{jt}{b}_t^{*}}\right|>{\left({b}_j^{*}\right)}^2 $$ and $$ 2{\displaystyle \sum_{\begin{array}{l}t=1\\ {}j\ne t\end{array}}^p{b}_j^{*}{r}_{jt}{b}_t^{*}}<0 $$ demonstrated that the correlation regulation will weaken the decision ability of the given pathway. In detail, each term 2*b*_j_^*^ *r*_*jt*_ *b*_t_^*^ can reflect the magnitude and direction of correlated regulation between pathways *x*_*ij*_ and *x*_*it*_. The larger the absolute value of this term is, the larger the correlated regulation between pathways *x*_*ij*_ and *x*_*it*_ is. The phenomenon that the sign of 2*b*_j_^*^ *r*_*jt*_ *b*_t_^*^ was negative revealed that the regulation was inhibited each other. Otherwise, the regulation was activated. Therefore, the complex regulation mechanisms among pathways can be demonstrated in numerical form through the subdivision of decision coefficient (Table 1). In addition, DC cut-off with the different significance level can be calculated according to the *t*-test statistics of DC as follows [32, 33]:

**Table 1:** The detailed subdivided result of decision coefficient

Subcategory/the secondary pathways	*x*_*i*1_	⋯	*x*_*ij*_	⋯	*x*_*it*_	⋯	*x*_*ip*_
Direct and indirect determination factor (*b*_j_^*^)^2^ and (2*b*_j_^*^ *r*_*jt*_ *b*_t_^*^) (*j*, *t* = 1, 2, ⋯, *p*; *j* ≠ *t*)	(***b***_1_^*^)^***2***^	⋯	2*b*_j_^*^ *r*_*j*1_ *b*_1_^*^	⋯	2*b*_t_^*^ *r*_*t*1_ *b*_1_^*^	⋯	2*b*_p_^*^ *r*_*p*1_ *b*_1_^*^
	⋮	⋱	⋮	⋮	⋮	⋮	⋮
	2*b*_1_^*^ *r*_1*j*_ *b*_j_^*^	⋯	(***b***_j_^*^)^***2***^	⋯	2*b*_t_^*^ *r*_*tj*_ *b*_j_^*^	⋯	2*b*_p_^*^ *r*_*pj*_ *b*_j_^*^
	⋮	⋮	⋮	⋱	⋮	⋮	⋮
	2*b*_1_^*^ *r*_1*t*_ *b*_t_^*^	⋯	2*b*_j_^*^ *r*_*jt*_ *b*_t_^*^	⋯	(***b***_t_^*^)^***2***^	⋯	2*b*_p_^*^ *r*_*pt*_ *b*_t_^*^
	⋮	⋮	⋮	⋮	⋮	⋱	⋮
	2*b*_1_^*^ *r*_1*p*_ *b*_p_^*^	⋯	2*b*_j_^*^ *r*_*jp*_ *b*_p_^*^	⋯	2*b*_t_^*^ *r*_*tp*_ *b*_p_^*^	⋯	(***b***_p_^*^)^***2***^
DC (*R*_(*j*)_)	*R*_(1)_	⋯	*R*_(*j*)_	⋯	*R*_(*t*)_	⋯	*R*_(*p*)_

*r*_*jt*_ (*j*, *t* = 1, 2, ⋯, *p*; *j* ≠ *t*) indicates the correlation coefficient *x*_*ij*_ and *x*_*it*_. Obviously, the data satisfy *r*_*jt*_ = *r*_*tj*_ and $$ {R}_{(j)}={\left({b}_j^{*}\right)}^2+2{\displaystyle \sum_{\begin{array}{l}t=1\\ {}j\ne t\end{array}}^p{b}_j^{*}{r}_{jt}{b}_t^{*}} $$ according to the decision analysis method. In order to distinguish between the direct and indirect determination factor clearly, the direct determination factor has been indicated in bold italics

3 $$ {R}_{(j) cut- off}=2{t}_p\left(n-q-1\right)\left|{r}_{jy}-{b}_j^{\ast}\right|\sqrt{\frac{c_{jj}\left(1-{R}^2\right)}{n-q-1}} $$

where *c*_*jj*_ is the *j*-th main diagonal element of inverse matrix of *R*_*x*_, *p* is the significance level (probability threshold), and *n* is the sample size, *q* is the number of independent variables. The *t*_*p*_(*n* − *q* − 1) is the upper (*p*/2) ‐ quantile of *t*-test statistics with degrees of freedom *n* − *q* − 1 under the given *p* probability threshold. This quantiles will increase when the probability threshold decreases due to *p* = *P*{|*t*| > *t*_*p*_(*n* − *q* − 1)}. The quantiles *t*_*p*_(*n* − *q* − 1) can be obtained by consulting the ‘Quantiles (Critical Values) for Student’s *t*-Distribution table’. For example, let *p* = 0.05, *t*_0.05_(*n* − *q* − 1) can be consulted, then *R*_(*j*)*cut* − *off*_ can be calculated. The results {|*R*_*obs*_| ≥ *R*_(*j*)*cut* − *off*_} demonstrate that the observation values (*R*_*obs*_) have statistical significance at the significant level of 0.05. Obviously, the two processes of *p* = *P*{|*R*_*obs*_| ≥ *R*_(*j*)*cut* − *off*_} ≤ 0.05 and {|*R*_*obs*_| ≥ *R*_(*j*)*cut* − *off*_} are equivalent.

**Fig. 2:**
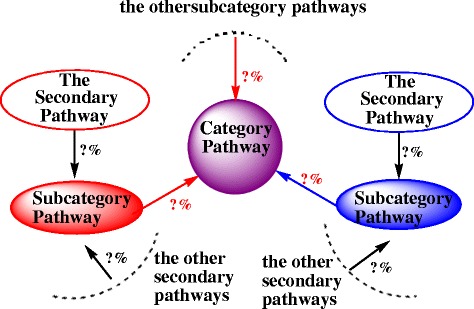
The decision tree of KEGG pathways according to the decision percentage. The red sign ‘?%’ denotes the decision percentage of KEGG subcategory pathway to its corresponding category pathway. Similarly, the black sign ‘?%’ denotes the decision percentage of the secondary KEGG pathway to its corresponding subcategory pathway. In addition, the activated KEGG subcategory pathways were marked with red color, the inhibited KEGG subcategory pathways were marked with blue color. In the same way, the activated secondary KEGG pathways were marked with red circles; the inhibited secondary KEGG pathways were marked with blue circles

The decision coefficient (DC) is a more scientific and comprehensive index conceived in reflecting the decision-making capability of the given pathway, and it truly reflects the variable decision of each independent variable to dependent variable. In this way, the most impacted pathway can be chosen. The larger the absolute value of DC of given pathway is, the larger the decision-making ability of given pathway to its upper level pathway. The value of DC can be positive or negative due to the positivity and negativity of indirect determination factor. Therefore, the sign of DC can characterize the impact direction of given pathway to a certain degree. Additionally, the decision tree was constructed below (Fig. 2) to display the decision results visually for biological researchers according to the decision percentage. The decision percentage (*dp*) is calculated as follows:

4 $$ dp=\frac{\left|{R}_{(j)}\right|}{{\displaystyle \sum_{j=1}^p\left|{R}_{(j)}\right|}}\times 100\% $$

### Demonstration of decision analysis on simulated data

To explain how the proposed decision analysis to identify the most significant pathways and to demonstrate the regulation among pathways, we performed computer simulations. The random data satisfied the assumption of normal distribution under the regression analysis. The detailed analysis results of simulated data were listed in Table 2.

**Table 2:** The decision analysis and path analysis results of simulated data

$$ \begin{array}{l}{x}_j\\ {}\mathrm{t}\mathrm{o}\\ {}y\end{array} $$	*b*_*j*_^∗^	*x*_*j*_ ↔ *x*_*k*_ → *y*	*r*_*jk*_ *b*_*k*_^∗^	2*b*_*j*_^∗^ *r*_*jk*_ *b*_*k*_^∗^	${\displaystyle \sum_{k\ne j}{r}_{jk}{b}_k^{\ast }}$	*r*_*jy*_	${\displaystyle \sum_{j\ne k}{r}_{kj}{b}_j^{\ast }}$	*R*_(*j*)_
$$ \begin{array}{l}{x}_1\\ {}\mathrm{t}\mathrm{o}\\ {}y\end{array} $$	0.383	*x*_1_ ↔ *x*_2_ → *y*	−0.162	−0.124	0.002	0.385	−0.700	0.148
		*x*_1_ ↔ *x*_3_ → *y*	2.504	1.916				
		*x*_1_ ↔ *x*_4_ → *y*	−2.340	−1.795				
$$ \begin{array}{l}{x}_2\\ {}\mathrm{t}\mathrm{o}\\ {}y\end{array} $$	−1.097	*x*_2_ ↔ *x*_1_ → *y*	0.057	−0.124	0.243	−0.854	−0.416	0.670
		*x*_2_ ↔ *x*_3_ → *y*	−0.198	0.433				
		*x*_2_ ↔ *x*_4_ → *y*	0.384	−0.842				
$$ \begin{array}{l}{x}_3\\ {}\mathrm{t}\mathrm{o}\\ {}y\end{array} $$	−2.593	*x*_3_ ↔ *x*_1_ → *y*	−0.369	1.196	2.009	−0.584	−0.278	−3.697
		*x*_3_ ↔ *x*_2_ → *y*	−0.084	0.434				
		*x*_3_ ↔ *x*_4_ → *y*	2.462	−12.772				
$$ \begin{array}{l}{x}_4\\ {}\mathrm{t}\mathrm{o}\\ {}y\end{array} $$	2.471	*x*_4_ ↔ *x*_1_ → *y*	−0.362	−1.795	−3.117	−0.647	0.506	−9.300
		*x*_4_ ↔ *x*_2_ → *y*	−0.170	−0.843				
		*x*_4_ ↔ *x*_3_ → *y*	−2.585	−12.799				

By fully incorporating the correlated structure of KEGG pathways, the decision analysis model shows distinctive advantages as below. First, the significant pathways can be identified through the DC cut-off calculated by formula (3) with the given significance level. To the given simulated data, when the significance level ( *p*-value) was set at 0.01, the two most significant subcategory pathways (*x*_3_ and *x*_4_) were identified. But when the significance level was set at 0.05, the three most significant subcategory pathways (*x*_2_, *x*_3_ and *x*_4_) were all selected. This result demonstrated that more significant pathways can be identified with the significance level increasing. Second, the direct and indirect determination factors from the DC can clearly display the correlated regulation among the pathways (*x*_1_, *x*_2_, *x*_3_ and *x*_4_). For illustrative purpose, we selected the most significant subcategory pathways *x*_3_ and *x*_4_ (*p* ≤ 0.01) to probe into the regulation mechanisms among pathways. As Table 2 shown, according to the subdivision of DC, the indirect regulations of *x*_1_ and *x*_2_ to *x*_3_ (2*b*_3_^∗^ *r*_31_ *b*_1_^∗^ = 2.046 and 2*b*_3_^∗^ *r*_32_ *b*_2_^∗^ = 0.4336) were all positive, but the regulation of *x*_4_ to *x*_3_ (2*b*_3_^∗^ *r*_34_ *b*_4_^∗^ = − 12.7715) was negative and far exceeded the positive regulation from *x*_1_ and *x*_2_. Thus, the total indirect determination factor of *x*_3_ ($2{\displaystyle \sum_{k\ne 3}{b}_3^{\ast }{r}_{3k}{b}_k^{\ast }}=-10.2918$) was negative. Obviously, the DC value of pathway *x*_3_ (*R*_(3)_ = − 3.6966) was negative because the total negative indirect determination from *x*_1_, *x*_2_ and *x*_4_ exceeded the direct determination of *x*_3_ ((*b*_3_^∗^)^2^ = 6.7252). However, the indirect regulations of *x*_1_, *x*_2_ and *x*_3_ to *x*_4_ were all negative. The larger negative indirect regulation led to the negative DC value. The detailed comparison of indirect regulation determination of *x*_1_, *x*_2_ and *x*_3_ to *x*_4_ showed that the negative regulation of pathway *x*_3_ was the largest and that of pathway *x*_2_ was the smallest. Third, the sign of DC value can be used to predict the impact direction of pathways. The calculated results of simulated data demonstrated that the impact direction of pathways *x*_1_ and *x*_2_ were up-regulated and that of the pathways *x*_3_ and *x*_4_ were down-regulated.

We have also compared the DC value (*R*_(*j*)_) of decision analysis and the total effect (*r*_*jy*_) of path analysis [28] based on the simulated data (Table 2). In general, both the path analysis and the decision analysis method emphasized the ‘correlation problem’ caused by the dependent structure among pathways. The path analysis demonstrated the identification of the significant pathways and the regulation among pathways through the total effect (*r*_*jy*_) and its subdivision. In fact, the regulations between pathways were mutual and non-equivalent. Take *x*_3_ and *x*_4_, for example, the regulation of *x*_4_ to *x*_3_ was positive (*r*_34_ *b*_4_^∗^ = 2.4624), conversely, the regulation of *x*_3_ to *x*_4_ was negative (*r*_43_ *b*_3_^∗^ = − 2.5847). The total effect of path analysis included the direct effect (*b*_*j*_^∗^) and indirect effect (${\displaystyle \sum_{k\ne j}{r}_{jk}{b}_k^{\ast }}$) of pathway *x*_*j*_, but ignored the retro-regulation of pathway *x*_*j*_ (${\displaystyle \sum_{j\ne k}{r}_{kj}{b}_j^{\ast }}$). The decision coefficient of decision analysis gave consideration to the regulation and retro-regulation of pathway *x*_*j*_ on the basis of the subdivision of the coefficient of determination of path analysis. For example, pathway *x*_2_ had a lower rank (fourth) according to the total effect of path analysis. In contrast, pathway *x*_2_ had a higher rank (first) according to the DC value of decision analysis due to the retro-regulation of pathway *x*_2_ to *x*_1_, *x*_3_ and *x*_4_. The strategy of borrowing information from retro-regulation allows the decision analysis to identify the most significant and mainly contributed pathways. It is true that there is no gold standard to compare the methods in real studies because the biological truth is unknown. Therefore, the analysis results based on the simulated data only help to illustrate the distinctive characteristics of decision analysis.

### Application

#### Datasets

The DIA impact values of the KEGG pathways from the functional analysis of the bovine mammary transcriptome during the lactation cycle were chosen to test the utility of decision analysis model [30]. The two most important pathway categories related to ‘Metabolism’ and ‘Environmental Information Processing’ were selected and discussed in detail in our analysis due to their high biological significance in bovine mammary [29, 30]. The decision analyses results of the other pathway categories were also attached in Additional file 1: Table S8. The detailed impact data of selected KEGG pathway categories and subcategories from -15 to 300 vs. -30d were shown in Additional file 2: Table S1. In addition, few pathways were deleted in that the number of missing data of these pathways was greater than or equal to three. Meanwhile, when the number of the missing data included in the pathway was less than three, they were filled with the average value of the other values belonging to this pathway. The filled data were marked in red color in Additional file 2: Table S1.

In order to compare the results of impact direction produced by the decision analysis model and the DIA method, the detailed impact direction data of selected KEGG pathway categories and subcategories from -15 to 300 vs. -30d were also listed in Additional file 3: Table S2. Similarly, the pathways including the missing data were processed as mentioned above.

## Results

The results of KEGG pathway categories and subcategories based on the subdivision of total CDs were shown in Table 3. The most impacted pathways identified according to different DC cutoff values were displayed in Additional file 4: Table S3. The comparison results of the most impacted pathways (DC value ≥ 0.4) under decision analysis model and DIA method were listed in Additional file 5: Table S4. The detailed comparison results of all pathways under the decision analysis model and DIA method were displayed in Additional file 6: Table S5. The DC subdivision results of selected KEGG pathway categories and subcategories were listed in Additional file 7: Table S6. The decision trees of selected pathway categories and subcategories were displayed in Additional file 8: Figure S1 according to the decision percentage.

**Table 3:** The percentage of direct and indirect CD in the total CD for selected KEGG pathway categories and subcategories

KEGG pathway category and subcategory	Total CD	
	direct CD	indirect CD
1. Metabolism	0.179	0.821
1.1 Carbohydrate Metabolism	0.168	0.832
1.2 Energy Metabolism	0.616	0.384
1.3 Lipid Metabolism	0.260	0.740
1.4 Nucleotide Metabolism	0.538	0.462
1.5 Amino Acid Metabolism	0.203	0.797
1.6 Metabolism of Other Amino Acids	0.478	0.523
1.7 Glycan Biosynthesis and Metabolism	0.238	0.762
1.8 Metabolism of Cofactors and Vitamins	0.379	0.621
1.11 Xenobiotics Biodegradation and Metabolism	0.453	0.547
3. Environmental Information Processing	0.512	0.488
3.2 Signal Transduction	0.139	0.861
3.3 Signaling Molecules and Interaction	0.364	0.636

### The subdivision results of total CDs

According to the path analysis approach, the total CD (*R* ^2^) of the selected KEGG pathway categories and subcategories had been calculated. The CDs (*R* ^2^) of subcategories ‘Energy Metabolism’ and ‘Metabolism of Other Amino Acids’ were 0.8613 and 0.9972, respectively. The CDs (*R* ^2^) of the other KEGG pathway categories and subcategories were almost up to 1. These results showed that the observed outcomes were replicated by the model very well.

The detailed ratios of direct and indirect CD for all selected pathways were shown in Table 3. For the selected KEGG pathway category, the indirect CD ratio of category ‘Metabolism’ (up to 82 %) was far greater than its corresponding direct CD ratio, indicating that the correlated regulations among pathways in this category were very important. On the contrary, the direct and indirect CD ratios of the other category ‘Environmental Information Processing’ (51 and 49 %) generally balanced, which showed that the direct and indirect effect were almost equally important. Similarly, the indirect CD ratios of almost all subcategory pathways were greater than their corresponding direct CD ratios. The exceptions were subcategories ‘Energy Metabolism’ and ‘Nucleotide Metabolism’ in category ‘Metabolism’. Among of them, the direct CD ratio of ‘Energy Metabolism’ was far greater than the indirect CD ratio. The direct CD ratio of ‘Nucleotide Metabolism’ was only slightly larger than its indirect CD ratio. In short, the fact that almost all indirect CD ratios were greater than their corresponding direct CD ratios further revealed that the complex regulating mechanisms existed and were very important in the KEGG pathways.

### The results of decision analysis

#### Identification of the most impacted pathways

The KEGG categories ‘Metabolism’ and ‘Environmental Information Processing’, including their all subcategories and the secondary pathways, were analyzed to test the utility of the decision analysis model. In order to use a more suitable DC cut-off to identify the most impacted pathways, the significance levels of 0.01, 0.05 and 0.1 were set to calculate the DC cut-off based on formula (3). The results showed that the different DC cut-offs were identified for different category and subcategory pathways. After integration, three DC cut-offs (0.3, 0.4, and 0.5) were chosen to compare (Additional file 4: Table S3). It should be noted that the cut-off of 0.3 satisfied the condition of *p* ≤ 0.1 for all category and subcategory pathways; the cut-off of 0.4 satisfied the condition of *p* ≤ 0.05 for a large majority category and subcategory pathways, with very few exceptions; the cut-off of 0.5 satisfied the condition of *p* ≤ 0.01 for only some of category and subcategory pathways, with some exceptions. The results of comparison showed that more suitable cut-off was ≥0.4. Therefore, when the absolute value of calculated DC for a pathway was greater than or equal to 0.4, this pathway was considered to be the most impacted.

As Additional file 5: Table S4 (a) shown, for KEGG pathway category ‘Metabolism’, its four subcategories are found to be the most activated pathways based on the DC values. The pathway with the highest positive DC value is ‘Lipid Metabolism’. Four subcategories are found to be the most inhibited pathways. Especially ‘Carbohydrate Metabolism’ has the largest negative decision capability. Differently, the three most impacted subcategories of category ‘Environmental Information Processing’ are all activated. The most impacted pathway is ‘Signal Transduction’, with the largest positive DC value.

As Additional file 5: Table S4 (b) shown, for the secondary pathways, the DC measure suggests that six pathways are the most impacted pathways (three activated; three inhibited) in subcategory ‘Lipid Metabolism’. Four pathways related to subcategory ‘Glycan Biosynthesis and Metabolism’ are found to be the most impacted (one activated; three inhibited). Five the secondary pathways of ‘Signal Transduction’ are the most impacted (four activated; one inhibited) according to the DC values.

In some cases, the most impacted pathways highlighted by the decision analysis model match our expectations. It is well known that the three main components of milk in dairy cow are lactose, fat and protein [29]. Thus, the presence of ‘Lipid Metabolism’ pathway with the highest positive DC value might be expected to appear, in that the lipid metabolism has something to do with the lactose synthesis. In other cases, the pathways are not immediately expected, but subsequent investigations revealed that these pathways identified by decision analysis are supported by previous experiment results. For example, the largest activation of ‘Glutathione metabolism’ in subcategory ‘Metabolism of Other Amino Acids’ appears to confirm previous data [34, 35], demonstrating that this process was very important in amino acids availability to mammary gland. In subcategory ‘Glycan Biosynthesis and Metabolism’, the secondary pathways related to ‘Glycosphingolipid biosynthesis’, particularly ganglio series, showed the largest decision-making ability in agreement with the findings reported by DIA method. In fact, the glycosphingolipid synthesized by these pathways have been reported to display beneficial health properties, especially for the defense of newborns against pathogens [36]. In addition, gangliosides have an important role in membrane function including cell signaling, cell adhesion and protein sorting [37]. In still other cases, no direct corroborative evidence could be found (e.g. for ‘Calcium signaling pathway’ in subcategory ‘Signal Transduction’). Thus, this finding serves as a hypothesis for future testing.

### Comparative analysis of KEGG pathways

In order to compare the results of the decision analysis method with those of the DIA approach, we checked the permutation order of DIA mean impact value of the most impacted pathways (DC value ≥ 0.4) and their impact directions. The details of compare results are listed in Additional file 5: Table S4. As a whole (Table 4), for the most impacted pathways comparison, the results showed that in about 85 % (11/13) of the selected pathway categories and subcategories, the concordance rate of the most impacted pathways under the two methods reaches or exceeds 50 %, even to 100 %. For the impact direction comparison, in 77 % (10/13) of the selected pathway categories and subcategories, the concordance rate of impact direction under the two methods reaches or exceeds 50 %, and in the remaining pathway categories and subcategories, the minimum concordance rate was 33.3 %.

**Table 4:** The comparison results of the most impacted pathways and impact direction under decision analysis model, KEGG-PATH and DIA method

	KEGG pathway Categories/Sub-categories		The concordance rate of impact direction		The concordance rate of the most impacted pathways	
			Decision analysis	KEGG-PATH	DIA	KEGG-PATH
1	1. Metabolism	For its sub-category pathways	54.5 %(6/11)	45.5 %(5/11)	62.5 % (5/8)	87.5 %(7/8)
2	3. Environmental Information Processing		100 %(3/3)	33.3 %(1/3)	0 (0/1)	100 %(1/1)
3	1.1 Carbohydrate Metabolism	For its secondary pathways	35.7 % (5/14)	50 %(7/14)	40 % (2/5)	50 % (3/6)
4	1.2 Energy Metabolism		33.3 %(1/3)	33.3 %(1/3)	100 %(2/2)	100 %(2/2)
5	1.3 Lipid Metabolism		76.9 %(10/13)	30.8 %(4/13)	50 %(3/6)	33.3 %(2/6)
6	1.4 Nucleotide Metabolism		50 %(1/2)	0 (0/2)	100 %(2/2)	100 %(2/2)
7	1.5 Amino Acid Metabolism		54.5 %(6/11)	72.7 %(8/11)	50 %(2/4)	60 %(3/5)
8	1.6 Metabolism of Other Amino Acids		75 %(3/4)	75 %(3/4)	100 %(2/2)	100 %(2/2)
9	1.7 Glycan Biosynthesis and Metabolism		58.3 %(7/12)	41.7 %(5/12)	50 %(2/4)	25 %(1/4)
10	1.8 Metabolism of Cofactors and Vitamins		37.5 %(3/8)	37.5 %(3/8)	60 %(3/5)	60 %(3/5)
11	1.11 Xenobiotics Biodegradation and Metabolism		66.7 %(2/3)	100 %(3/3)	50 %(1/2)	100 %(2/2)
12	3.2 Signal Transduction		63.6 %(7/11)	63.6 %(7/11)	60 %(3/5)	60 %(3/5)
13	3.3 Signaling Molecules and Interaction		66.7 %(2/3)	33.3 %(1/3)	100 %(3/3)	100 %(3/3)

For the ‘The concordance rate of impact direction’ column, the denominator of each fraction in the parentheses denotes the number of subcategory pathways and the secondary pathways from the front corresponding categories and sub-categories for two columns, and the numerator of each fraction for two columns denotes the number of pathways with the same impact direction under DIA and decision analysis, and under DIA and KEGG-PATH respectively. For the ‘The concordance rate of the most impacted pathways’ column, the denominator of each fraction in the parentheses denotes the number (*a*) of the most impacted pathways identified based on DC values in corresponding pathway categories and sub-categories for two columns, and the numerator of each fraction for two columns denotes the number of pathways which also appeared in top *a* pathways identified by DIA average impact values and by total effect from KEGG-PATH, respectively

In addition, we roughly compared the results of the decision analysis with those of KEGG-PATH approach. The results demonstrated that the concordance rate of pathway impact direction under decision analysis was significantly higher than that under KEGG-PATH approach when DIA pathway impact directions were used as standard. For example, for pathway categories ‘Metabolism’ and ‘Environmental Information Processing’, the concordance rates of pathway impact direction under decision analysis were 54.5 % (6/11) and 100 % (3/3), respectively. However, the corresponding concordance rates under KEGG-PATH were 45.5 % (5/11) and 33.3 % (1/3). From the view of all the secondary pathways belonging to the same category, the concordance rates of pathway impact direction were also obviously improved from 49.3 % (34/69) and 57.1 % (8/14) under KEGG-PATH to 58 % (40/69) and 64.3 % (9/14) under decision analysis for categories ‘Metabolism’ and ‘Environmental Information Processing’, respectively. For the most impacted pathways comparison, the concordance rate compared with KEGG-PATH seemed still higher than that compared with DIA (Table 4). Based on this comparison, several distinctions between the three approaches can be made.

First, overwhelming majority of the most relevant function pathways in the mammary gland during lactation are captured based on DC values (Additional file 5: Table S4). Some of them also were found by mean impact values in DIA approach and by total effect in KEGG-PATH approach. For example, for the subcategories ‘Energy Metabolism’, ‘Nucleotide Metabolism’, ‘Metabolism of Other Amino Acids’ and ‘Signaling Molecules and Interaction’, the most impacted secondary pathways are almost the same under the three methods. The results showed that the correlation regulations strengthen the direct determination of these secondary pathways to some extent. These results also can be confirmed by the subdivision of decision coefficient. (Additional file 7: Table S6 (b)) In addition, the decision analysis method highlights some more biologically meaningful results. For example, the ‘Lipid Metabolism’ subcategory has the largest positive DC value. This result is potentially the most interesting given the strong literature support described above. The largely impacted pathway ‘Glycosphingolipid biosynthesis–ganglio series’ was also present in the results as we expected due to its importance role of modulating enzyme properties, cell signaling, cell adhesion [30]. It is interesting that the inhibition of gangliosides presents in the results. This is in consistent with the fact that the concentration of glycosphingolipids showed a large decrease during the transition from colostrums to mature milk [38].

Second, the pathways were identified as the most impacted pathways based on the DC values, but they were not found according to the mean DIA impact value. For example, two subcategories, ‘Nucleotide Metabolism’ and ‘Metabolism of Other Amino Acids’, are not the most impacted by DIA mean impact values, but are demonstrated to be the most impacted based on the DC values. The subdivision results of decision coefficients (Additional file 7: Table S6 (a)) showed that subcategory ‘Nucleotide Metabolism’ has the relatively larger direct determination, and is positively regulated by pathways ‘Lipid Metabolism’ and ‘Metabolism of Other Amino Acids’ to a large extent. The subcategory ‘Metabolism of Other Amino Acids’ is also largely positively regulated by ‘Nucleotide Metabolism’ and ‘Lipid Metabolism’. These subdivision results revealed that the correlation regulation among pathways highlights the importance of these two subcategories. In category ‘Environmental Information Processing’, subcategory ‘Signal Transduction’ is unexpectedly ranked the first according to the DC value. On the contrary, this subcategory has the smallest average impact value in DIA approach. Obviously, ‘Signal Transduction’ is very important to the mammary gland during lactation [30]. These three sub-categories, ‘Nucleotide Metabolism’ and ‘Metabolism of Other Amino Acids’ and ‘Signal Transduction’, also were selected as the most impacted pathways under KEGG-PATH approach. The phenomenon showed that the correlation regulation was very important in identifying the most impacted pathways. In addition, the strongly inhibition of pathways ‘Lysine degradation’ and ‘Tryptophan metabolism’ appeared to be consistent with the inhibition of ‘Citrate cycle (TCA cycle)’. The result can be supported by the fact that many of the products of these two pathways could be precursors of TCA cycle pathway [30]. The secondary pathways ‘Fatty acid elongation in mitochondria’, ‘Fatty acid metabolism’ and ‘Steroid hormone biosynthesis’ in the subcategory ‘Lipid Metabolism’ are selected as the most impacted pathways based on the DC values. But their DIA mean impact values are relatively small. The result indicated that the correlation regulation has resulted in the change of the importance of these pathways. But it was strange that these three secondary pathways were not selected as the most impacted pathways according to KEGG-PATH. The result demonstrated that the retro-regulation among these pathways should be very important. Therefore, researchers should pay much more attention to these correlation regulations. To shed light on the difference, we checked the subdivision of decision coefficient. (Additional file 7: Table S6 (b)) The results showed that the ‘Fatty acid elongation in mitochondria’ and ‘Fatty acid metabolism’ pathways were inhibited in that they were negatively regulated by pathway ‘Arachidonic acid metabolism’ to a great extent. The reduction of fatty acid metabolism also can be supported by the fact that the fatty acid taken up by the mammary tissue mainly was used towards the synthesis of milk fat, including the components of cellular membranes [30, 39]. Conversely, few of the most impacted pathways based on mean DIA impact values were not found according to DC values. For example, the subcategories ‘Metabolism of Terpenoids and Polyketides’ and ‘Biosynthesis of Other Secondary Metabolites’ in category ‘Metabolism’ were not found based on DC values. As Additional file 9: Table S7 (a) showed that the two subcategories have very small direct determination and were slightly regulated by the other pathways. The importance of these two subcategories was weakened just because of the approximate balance of direct and indirect determination. Although the secondary pathways ‘Hedgehog signaling pathway’ and ‘TGF-beta signaling pathway’ in subcategory ‘Signal Transduction’ were not found to be the most impacted pathways based on the DC values, they would be selected when the cut-off of ≥ 0.3 was used.

Third, the results based on the decision analysis model were displayed through the construction of decision tree (Additional file 8: Figure S1). In the decision tree ‘network’, the activated KEGG subcategory pathways were marked with red color, the inhibited KEGG subcategory pathways were marked with blue color. Similarly, the activated secondary KEGG pathways were marked with red circles; the inhibited secondary KEGG pathways were marked with blue circles. Meanwhile, the red and black numbers were used to denote the decision percentage of KEGG subcategory pathway to its corresponding category pathway, the decision percentage of the secondary KEGG pathway to its corresponding subcategory pathway, respectively. In this way, the researchers can catch important information fleetly and exactly.

### Why does decision analysis perform better prediction effects?

To explain why decision analysis outperforms the KEGG-PATH approach, we calculated the retro-regulation of each pathway in detail and listed all the results of decision analysis and KEGG-PATH approach in Additional file 9: Table S7.

The first superiority of the decision analysis model was that the retro-regulation of each pathway was considered in identifying the most significant pathways based on the coefficient of determination. As Additional file 9: Table S7 (a) showed, in category pathway “Environmental Information Processing”, the total effects of the three subcategories were all relatively large by KEGG-PATH method and there was few difference among them; but their DC values had very big difference when the retro-regulation was considered. More importantly, the selected significant subcategory pathway “Signal Transduction” based on the DC values was highly impacted indeed as the documents reported during the lactation [30]. Obviously, the calculation results was also demonstrated that the retro-regulation of “Signal Transduction” was relatively larger compared to the other subcategory pathways. Thus, the positive mutual regulation highlighted the significance of pathway “Signal Transduction”. Differently, the subcategory pathway “Lipid Metabolism” was negatively regulated by the other subcategory pathways; conversely, the pathway “Lipid Metabolism” had larger positive retro-regulation on the other subcategory pathways and the positive direct effect. Thus, the pathway “Lipid Metabolism” had the positive decision-making ability. Similarly, the subcategory pathways “Carbohydrate Metabolism” and “Glycan Biosynthesis and Metabolism” were positively regulated by the other subcategory pathways; conversely, they had larger negative retro-regulation on the other subcategory pathways and the negative direct effect. Thus, the two pathways had the largely negative decision-making ability.

Another superiority of the decision analysis model was that the impact directions of pathways could be estimated preliminarily and directly according to the sign (positive or negative) of the DC. Still further, the sign of the DC also gave consideration to the dependences among the pathways. In this study, the result of the decision analysis showed that the subcategory pathway “Lipid Metabolism” had the largest positive decision-making ability; however, the impact direction of its secondary pathway ‘Fatty acid metabolism’ was negative. These results match our expectations because the lipid metabolism had a lot to do with the synthesis of the lactose and the reduction of fatty acid metabolism was considered towards the synthesis of milk fat through taking up the fatty acids by the mammary tissue. Besides, the fact that the impact direction of the TGF-beta pathway was negative based on the decision analysis was in accordance with the fact that this pathway appeared to have a negative role on mammary cell proliferation [40].

## Discussion

In this study, a decision analysis model is first proposed to identify the most impacted pathways. The decision analysis model borrows the decision coefficient to judge the importance of the pathways, which not only considers the direct determination factor of pathway itself, but also adds the correlation indirect determination factor with the other related pathways. Compared with DIA approach, the decision analysis method overcomes the deficiency of analyzing each pathway independently. Compared with KEGG-PATH approach, the decision analysis method constructs a DC index based on the coefficient of determination of regression analysis, rather than correlation coefficient. Importantly, the retro-regulation among pathways was considered in decision analysis. Therefore, the decision analysis model is a statistical data mining at a deeper level. For the estimation of impact direction, the DIA method averages the impact direction values of the pathway during different time course. The KEGG-PATH approach needs to use the gradient analysis from principal component analysis (PCA) to estimate the impact directions of pathways. However, the decision analysis can judge the impact direction directly through the sign of decision coefficient. More importantly, the sign of decision coefficient was caused by the correlated regulation from the other related pathways. Thus, the identification of pathway impact direction (up-regulating or down-regulating) through the decision coefficient also gave consideration to the dependences among the pathways. Hence, it is a major bright spot of the decision analysis model that the identification of the most impacted pathways and their impact directions through the decision coefficient took account of the correlation among pathways from the angle of ‘variation determination’.

In addition, the regulation mechanisms among pathways can be demonstrated through the subdivision of decision coefficient. This numerical expression of the correlation regulation among pathways is another major highlight of the decision analysis model. The construction of decision tree can visually display the results of decision analysis. We have developed a program in Matlab (R2008a, version 7.6.0.324) to implement the decision analysis (Additional file 10: S1). In the calculations, we found that the results might be inaccurate when the correlation matrix was close to singular or badly scaled. But the relative error is basically controlled to 10^−15^ and it can be neglected.

Although the decision analysis model is designed to analyze the KEGG pathways, it is theoretically also applicable to the other databases with similar dependency structure, such as Reactome, Wikipathways, etc. However, considering the information about how cell and tissue type, age, and environmental exposures affect pathway interactions, how to apply the decision analysis to general cases with the original gene expression value rather than the DIA impact values is still a challenge. In order to better understand large biological system, addressing these issues, coupled with technological advances will likely improve the confidence in results.

## Conclusions

The decision coefficient (DC) based on coefficient of determination (CD) of regression analysis gives consideration to the inter-pathway dependence in identifying the most impacted pathways and their impact directions. Meanwhile, the regulation mechanisms among pathways were demonstrated from the angle of ‘variation determination’. The decision analysis model is an initial attempt of optimizing pathway analysis methodology.

## Additional files

Additional file 1: Table S8. This file provides the original DIA impact values and the detailed subdivided results of decision coefficient for the other KEGG subcategory pathways and the other KEGG secondary pathways in Table S8 (a)–(d), respectively. In order to distinguish between the direct and indirect determination factor clearly, the direct determination factor has been indicated in red box. (DOCX 67 kb)
Additional file 2: Table S1. This file provide the detailed impact data of selected KEGG pathway categories, subcategories and the secondary pathways from− 15 to 300 vs.− 30d in bovine mammary tissue during lactation in Table S1 (a) and (b), respectively. The numbers colored in red color are the filled data by the average of all the other impact values in this pathway, which is the missing data originally. (DOCX 35 kb)
Additional file 3: Table S2. This file provide the detailed impact direction data of selected KEGG pathway categories, subcategories and the secondary pathways from− 15 to 300 vs.− 30d in bovine mammary tissue during lactation in Table S2 (a) and (b), respectively. The numbers colored in red color are the filled data by the average of all the other impact values in this pathway, which is the missing data originally. (DOCX 35 kb)
Additional file 4: Table S3. The file gives the comparison results of the most impacted pathways identified according to different Decision Coefficient (DC) values. (DOCX 24 kb)
Additional file 5: Table S4. This file gives the comparison of the most impacted pathway subcategories and the most impacted secondary pathways (DC value ≥ 0.4) under decision analysis model and DIA method in Table S4 (a) and (b), respectively. In ‘Group’ column, ‘a’ showed that the pathway was the most impacted pathway under both decision analysis model and DIA method; ‘b’ showed that the pathway was the most impacted pathways only under decision analysis model, and was not under DIA method. ‘Rank_*DIA*_’ was the order of mean impact value for the pathway. The sign “+” and “−” represent the up-regulating and down-regulating impact direction, respectively. (DOCX 30 kb)
Additional file 6: Table S5. The file gives the comparison results of all the selected subcategory pathways and all the selected secondary pathways under DIA method and Decision analysis method in Table S5 (a) and (b), respectively. The sign “+” and “−” represent the up-regulating and down-regulating impact direction, respectively. (DOCX 36 kb)
Additional file 7: Table S6. This file provides the detailed subdivided results of decision coefficient for the selected KEGG subcategory pathways and the selected KEGG secondary pathways in Table S6 (a) and (b), respectively. In order to distinguish between the direct and indirect determination factor clearly, the direct determination factor has been indicated in red box. (DOCX 59 kb)
Additional file 8: Figure S1. The file gives the decision trees of selected pathway categories and subcategories plotted according to the decision percentage. (for (a) Metabolism and (b) Environmental Information Processing) The activated KEGG subcategory pathways were marked with red color, the inhibited KEGG subcategory pathways were marked with blue color. In the same way, the activated secondary KEGG pathways were marked with red circles; the inhibited secondary KEGG pathways were marked with blue circles. (DOCX 431 kb)
Additional file 9: Table S7. This file provides the decision analysis and path analysis results of the selected category pathways and the selected subcategory pathways in Table S7 (a) and (b). (DOCX 1278 kb)
Additional file 10: S1. This file provides the code for the decision analysis model in Matlab (R2008a, version 7.6.0.324). (DOCX 11 kb)
