# Supplementary material for: A decision analysis model for KEGG pathway analysis
Source: BMC Bioinformatics. 2016 Oct 6;17:407. doi: 10.1186/s12859-016-1285-1 (PMC5053338; doi:10.1186/s12859-016-1285-1)
Supplement: Additional file 1: Table S8. — This file provides the original DIA impact values and the detailed subdivided results of decision coefficient for the other KEGG subcategory pathways and the other KEGG secondary pathways in Table S8 (a)–(d), respectively. In order to distinguish between the direct and indirect determination factor clearly, the direct determination factor has been indicated in red box. (DOCX 67 kb) [file 12859_2016_1285_MOESM10_ESM.docx]

**Table S8 (a)** The detailed impact data of KEGG pathway categories and subcategories from -15 to 300 vs. -30d in bovine mammary tissue during lactation.

| **The categories and subcategories** | **Impact data** | | | | | | | |
| --- | --- | --- | --- | --- | --- | --- | --- | --- |
|  | **-15vs-30** | **1vs-30** | **15vs-30** | **30vs-30** | **60vs-30** | **120vs-30** | **240vs-30** | **360vs-30** |
| **2. Genetic Information Processing** | 29.01225 | 59.45205 | 45.5399 | 66.78752 | 104.2094 | 93.087316 | 28.197056 | 26.77298 |
| 2.1 Transcription | 18.08969 | 35.95684 | 27.42328 | 44.71803 | 78.51178 | 74.964483 | 13.083188 | 11.93853 |
| 2.2 Translation | 47.33266 | 57.32102 | 48.5645 | 73.91251 | 103.8245 | 104.92385 | 22.158949 | 27.90119 |
| 2.3 Folding, Sorting and Degradation | 19.28328 | 61.81901 | 49.5252 | 66.31969 | 102.4447 | 99.692027 | 24.417172 | 24.92087 |
| 2.4 Replication and Repair | 28.92013 | 70.21404 | 47.42818 | 72.43058 | 119.4377 | 82.877884 | 47.839345 | 35.10215 |
| **4. Cellular Processes** | 21.00953 | 77.63061 | 78.0869 | 85.51778 | 147.9573 | 122.93389 | 47.182412 | 35.75154 |
| 4.1 Transport and Catabolism | 23.81129 | 85.993 | 89.56679 | 94.8448 | 163.8031 | 115.98393 | 50.440697 | 38.21824 |
| 4.2 Cell Motility | 17.58833 | 61.91513 | 48.14498 | 73.21768 | 139.2492 | 142.24465 | 46.69402 | 30.41049 |
| 4.3 Cell Growth and Death | 21.28725 | 73.14617 | 81.78033 | 81.41566 | 135.3701 | 127.22432 | 40.269125 | 37.30837 |
| 4.4 Cell Communication | 18.78536 | 75.59092 | 67.52907 | 81.03614 | 142.9142 | 122.50323 | 50.144941 | 32.4466 |
| **5. Organismal Systems** | 24.38492 | 91.86445 | 92.20447 | 100.0193 | 166.3564 | 145.78139 | 46.063706 | 39.86538 |
| 5.1 Immune System | 24.49465 | 86.43688 | 78.55136 | 104.9152 | 177.1679 | 150.85335 | 37.914993 | 47.33609 |
| 5.2 Endocrine System | 24.51365 | 125.6249 | 116.3495 | 122.4282 | 215.1971 | 170.22358 | 60.498675 | 47.93448 |
| 5.3 Circulatory System | 17.60523 | 66.0782 | 63.41387 | 65.29133 | 101.289 | 107.14785 | 56.740204 | 27.20796 |
| 5.4 Digestive System | 23.4884 | 88.25928 | 105.1363 | 90.06987 | 150.4517 | 135.47008 | 29.921965 | 30.83602 |
| 5.5 Excretory System | 24.44551 | 103.007 | 125.4215 | 110.7031 | 168.2854 | 187.73929 | 87.337454 | 39.24459 |
| 5.6 Nervous System | 28.45942 | 90.9831 | 79.40237 | 89.29623 | 148.4604 | 127.49941 | 25.893301 | 25.92762 |
| 5.7 Sensory System | 27.92789 | 84.62284 | 81.24336 | 76.38106 | 134.1434 | 86.436673 | 57.290086 | 50.02818 |
| 5.8 Development | 27.21408 | 93.31438 | 69.16293 | 97.16665 | 153.1451 | 131.63415 | 47.075955 | 41.36885 |
| 5.9 Environmental Adaptation | 17.79245 | 5.438773 | 34.39059 | 50.38982 | 69.36291 | 33.800125 | 49.51526 | 13.5956 |

**Table S8 (b)** The detailed impact data of the secondary KEGG pathways from -15 to 300 vs. -30d in bovine mammary tissue during lactation.

| **The secondary pathways** | **Impact data** | | | | | | | |
| --- | --- | --- | --- | --- | --- | --- | --- | --- |
|  | **-15vs-30** | **1vs-30** | **15vs-30** | **30vs-30** | **60vs-30** | **120vs-30** | **240vs-30** | **360vs-30** |
| **2.1 Transcription** |  |  |  |  |  |  |  |  |
| 2.1.1 Basal transcription factors | 27.6247 | 13.6710 | 19.8353 | 34.9367 | 62.4781 | 56.1516 | 10.8713 | 16.1225 |
| 2.1.2 RNA polymerase | 18.6325 | 50.6165 | 35.0510 | 51.5730 | 85.2643 | 68.2125 | 22.2699 | 7.0090 |
| 2.1.3 Spliceosome | 8.0118 | 43.5830 | 27.3835 | 47.6443 | 87.7930 | 100.5293 | 6.1084 | 12.6841 |
| **2.2 Translation** |  |  |  |  |  |  |  |  |
| 2.2.1 Aminoacyl-tRNA biosynthesis | 29.9700 | 88.2182 | 90.9813 | 71.8975 | 85.4941 | 95.2067 | 29.1410 | 20.1142 |
| 2.2.2 mRNA surveillance pathway | 5.5786 | 92.2371 | 63.9552 | 110.8202 | 157.8990 | 150.8313 | 20.5008 | 26.0601 |
| 2.2.3 Ribosome | 146.4002 | 14.7469 | 21.1503 | 65.6618 | 123.7840 | 95.1985 | 33.2958 | 29.3736 |
| 2.2.4 Ribosome biogenesis in eukaryotes | 28.7883 | 44.5355 | 28.5171 | 58.6097 | 79.3972 | 90.4277 | 2.6080 | 14.8726 |
| 2.2.5 RNA transport | 25.9263 | 46.8674 | 38.2185 | 62.5733 | 72.5484 | 92.9551 | 25.2491 | 49.0855 |
| **2.3 Folding, Sorting and Degradation** |  |  |  |  |  |  |  |  |
| 2.3.1 Proteasome | 23.0468 | 34.5102 | 18.1886 | 23.8841 | 44.5052 | 77.9248 | 8.8279 | 34.5815 |
| 2.3.2 Protein export | 86.6874 | 124.4562 | 96.2146 | 81.6850 | 125.6816 | 81.5519 | 86.6874 | 10.5349 |
| 2.3.3 Protein processing in endoplasmic reticulum | 10.0443 | 72.3292 | 62.6196 | 70.3863 | 131.8188 | 108.8753 | 39.1677 | 38.4607 |
| 2.3.4 RNA degradation | 19.5734 | 50.2250 | 29.5821 | 60.8702 | 66.8910 | 101.8864 | 30.8860 | 12.2964 |
| 2.3.5 SNARE interactions in vesicular transport | 29.3199 | 69.3502 | 36.0728 | 55.4254 | 77.3043 | 93.1490 | 24.7521 | 39.2650 |
| 2.3.6 Sulfur relay system | 17.0479 | 27.2995 | 49.1947 | 116.6933 | 168.6621 | 132.1567 | 26.3758 | 76.7757 |
| 2.3.7 Ubiquitin mediated proteolysis | 16.6673 | 54.5628 | 54.8040 | 55.2935 | 102.2496 | 102.3000 | 16.4936 | 14.3867 |
| **2.4 Replication and Repair** |  |  |  |  |  |  |  |  |
| 2.4.1 Base excision repair | 31.3711 | 59.8128 | 30.8209 | 59.4510 | 62.8045 | 65.9173 | 32.9073 | 36.1362 |
| 2.4.2 DNA replication | 26.9354 | 76.5538 | 81.0965 | 82.7432 | 129.0976 | 109.4388 | 53.5854 | 46.6473 |
| 2.4.3 Homologous recombination | 28.3723 | 97.2052 | 53.4809 | 126.9121 | 193.7701 | 111.4630 | 67.7248 | 41.6450 |
| 2.4.4 Mismatch repair | 29.2393 | 54.0388 | 39.0646 | 64.0497 | 66.9435 | 60.5363 | 53.1814 | 27.3978 |
| 2.4.5 Non-homologous end-joining | 69.2157 | 77.5233 | 28.4587 | 30.0641 | 171.2605 | 69.2157 | 69.2457 | 38.7717 |
| 2.4.6 Nucleotide excision repair | 28.6826 | 56.1503 | 51.6476 | 71.3636 | 92.7501 | 67.0341 | 31.7979 | 20.0149 |
| **4.1 Transport and Catabolism** |  |  |  |  |  |  |  |  |
| 4.1.1 Endocytosis | 22.5098 | 49.7486 | 58.9954 | 59.1782 | 117.4333 | 88.5926 | 41.1411 | 29.0780 |
| 4.1.2 Lysosome | 20.4253 | 59.3815 | 49.9510 | 66.8025 | 129.9858 | 97.3483 | 36.6775 | 37.1362 |
| 4.1.3 Peroxisome | 32.9660 | 108.6937 | 138.5018 | 135.2491 | 232.5274 | 170.5600 | 58.9020 | 46.8933 |
| 4.1.4 Phagosome | 19.3441 | 107.6980 | 108.3229 | 131.0068 | 196.7680 | 184.5880 | 103.2921 | 72.5338 |
| 4.1.5 Regulation of autophagy | 68.1809 | 104.4431 | 92.0629 | 81.9873 | 142.3012 | 38.8308 | 12.1907 | 5.4499 |
| **4.3 Cell Growth and Death** |  |  |  |  |  |  |  |  |
| 4.3.1 Apoptosis | 24.7850 | 56.6594 | 55.8005 | 65.0792 | 125.9284 | 112.5841 | 41.4237 | 33.9571 |
| 4.3.2 Cell cycle | 13.5036 | 86.7704 | 88.6280 | 88.3041 | 135.0274 | 126.0311 | 36.8813 | 37.6037 |
| 4.3.3 Oocyte meiosis | 15.6111 | 79.3127 | 68.9021 | 91.5868 | 140.6267 | 112.6886 | 26.0484 | 26.9444 |
| 4.3.4 p53 signaling pathway | 31.2494 | 69.8421 | 113.7907 | 80.6926 | 139.8981 | 157.5936 | 56.7231 | 50.7283 |
| **4.4 Cell Communication** |  |  |  |  |  |  |  |  |
| 4.4.1 Adherens junction | 14.7221 | 51.1057 | 42.7151 | 57.5265 | 87.1063 | 81.5489 | 44.6887 | 23.2475 |
| 4.4.2 Focal adhesion | 17.3666 | 61.2405 | 63.7557 | 77.7385 | 144.0062 | 121.1237 | 65.3078 | 37.0423 |
| 4.4.3 Gap junction | 25.2270 | 94.8339 | 72.0472 | 93.0751 | 165.8898 | 144.0317 | 45.0338 | 22.5329 |
| 4.4.4 Tight junction | 17.8257 | 95.1835 | 91.5983 | 95.8045 | 174.6544 | 143.3086 | 45.5495 | 46.9637 |
| **5.1 Immune System** |  |  |  |  |  |  |  |  |
| 5.1.1 Antigen processing and presentation | 24.9246 | 119.1127 | 115.9640 | 212.4793 | 347.9441 | 327.9763 | 93.4834 | 123.9742 |
| 5.1.2 B cell receptor signaling pathway | 26.4566 | 74.0560 | 63.5314 | 78.8397 | 153.9693 | 128.1762 | 43.5977 | 34.9818 |
| 5.1.3 Chemokine signaling pathway | 20.2963 | 82.2162 | 73.4752 | 87.6151 | 139.1736 | 127.5344 | 37.4401 | 42.9790 |
| 5.1.4 Complement and coagulation cascades | 7.1384 | 81.7695 | 117.6822 | 133.9028 | 179.5708 | 146.6077 | 55.1171 | 59.9689 |
| 5.1.5 Cytosolic DNA-sensing pathway | 36.7138 | 40.1017 | 47.1312 | 44.9932 | 112.2147 | 68.7512 | 3.4967 | 19.9231 |
| 5.1.6 Fc epsilon RI signaling pathway | 19.3481 | 103.3176 | 86.3545 | 139.1239 | 249.5420 | 193.4499 | 45.7887 | 49.3701 |
| 5.1.7 Fc gamma R-mediated phagocytosis | 13.2291 | 79.9740 | 59.7775 | 95.8548 | 169.3161 | 157.7590 | 47.3323 | 37.8674 |
| 5.1.8 Hematopoietic cell lineage | 35.2650 | 125.9446 | 133.5587 | 135.5418 | 221.5530 | 165.1029 | 46.6374 | 72.4061 |
| 5.1.9 Intestinal immune network for IgA production | 24.7061 | 105.2775 | 96.5048 | 120.4796 | 191.2465 | 172.2098 | 35.8436 | 53.8181 |
| 5.1.10 Leukocyte transendothelial migration | 13.5869 | 72.5502 | 64.8354 | 72.8391 | 136.9136 | 128.4893 | 33.8666 | 39.5842 |
| 5.1.11 Natural killer cell mediated cytotoxicity | 24.4738 | 102.6260 | 82.8547 | 120.1266 | 165.9205 | 172.5044 | 29.1594 | 43.6724 |
| 5.1.12 NOD-like receptor signaling pathway | 20.8794 | 81.9792 | 59.6835 | 90.5166 | 146.6598 | 139.6451 | 17.3259 | 28.2089 |
| 5.1.13 RIG-I-like receptor signaling pathway | 35.7352 | 72.9792 | 52.9821 | 68.1060 | 166.9535 | 97.4729 | 21.2056 | 32.1585 |
| 5.1.14 T cell receptor signaling pathway | 34.6984 | 76.3181 | 58.1454 | 80.3669 | 138.3962 | 115.8473 | 27.6688 | 29.6781 |
| 5.1.15 Toll-like receptor signaling pathway | 29.9681 | 78.3307 | 65.7896 | 92.9432 | 138.1441 | 121.2739 | 30.7618 | 41.4507 |
| **5.2 Endocrine System** |  |  |  |  |  |  |  |  |
| 5.2.1 Adipocytokine signaling pathway | 30.4134 | 137.8819 | 153.4261 | 144.2353 | 244.2621 | 192.5327 | 72.3407 | 45.8904 |
| 5.2.2 GnRH signaling pathway | 27.9087 | 122.4916 | 92.9678 | 122.1599 | 216.8166 | 166.0279 | 26.7129 | 29.2052 |
| 5.2.3 Insulin signaling pathway | 19.4422 | 77.0145 | 61.4148 | 74.2447 | 136.6769 | 120.8497 | 42.0164 | 33.5119 |
| 5.2.4 Melanogenesis | 33.9943 | 88.9745 | 49.5073 | 83.4333 | 129.7480 | 138.7395 | 28.7844 | 26.8935 |
| 5.2.5 PPAR signaling pathway | 22.0233 | 257.8563 | 355.2313 | 312.6265 | 433.3840 | 368.0951 | 161.2477 | 126.6981 |
| 5.2.6 Renin-angiotensin system | 13.2999 | 69.5303 | 55.8454 | 78.7198 | 135.4942 | 114.6632 | 31.8898 | 25.4077 |
| **5.3 Circulatory System** |  |  |  |  |  |  |  |  |
| 5.3.1 Cardiac muscle contraction | 9.7679 | 55.0501 | 60.1010 | 53.9839 | 90.7023 | 98.8524 | 78.3310 | 27.7165 |
| 5.3.1 Vascular smooth muscle contraction | 25.4425 | 77.1063 | 66.7268 | 76.5988 | 111.8758 | 115.4433 | 35.1494 | 26.6994 |
| **5.4 Digestive System** |  |  |  |  |  |  |  |  |
| 5.4.1 Bile secretion | 46.7982 | 127.5758 | 135.7992 | 134.3431 | 212.1350 | 201.1753 | 40.3591 | 15.7304 |
| 5.4.2 Carbohydrate digestion and absorption | 6.1399 | 58.7890 | 103.9118 | 60.9106 | 85.7570 | 84.4818 | 6.2881 | 58.0398 |
| 5.4.3 Fat digestion and absorption | 21.2833 | 125.8631 | 117.4985 | 100.3651 | 199.0327 | 182.6429 | 38.0233 | 43.1205 |
| 5.4.4 Gastric acid secretion | 19.2825 | 91.7550 | 82.0455 | 83.3381 | 149.8280 | 155.2970 | 19.7018 | 22.2308 |
| 5.4.5 Mineral absorption | 29.8183 | 53.4733 | 92.6053 | 72.7387 | 104.3349 | 98.5519 | 49.7342 | 18.1891 |
| 5.4.6 Pancreatic secretion | 41.3669 | 88.7042 | 101.0493 | 85.8202 | 165.7897 | 162.1501 | 21.3276 | 25.2635 |
| 5.4.7 Protein digestion and absorption | 6.4086 | 72.0141 | 143.2536 | 88.4057 | 126.0439 | 100.3847 | 47.6758 | 61.3188 |
| 5.4.8 Salivary secretion | 21.4685 | 115.2198 | 119.7488 | 109.8644 | 200.2214 | 179.6704 | 16.2658 | 29.9990 |
| 5.4.9 Vitamin digestion and absorption | 18.8295 | 60.9393 | 50.3143 | 74.8430 | 110.9225 | 54.8765 | 61.7875 | 61.7875 |
| **5.5 Excretory System** |  |  |  |  |  |  |  |  |
| 5.5.1 Aldosterone-regulated sodium reabsorption | 14.2778 | 78.1081 | 105.2802 | 78.1513 | 154.3094 | 158.7702 | 3.4739 | 6.3419 |
| 5.5.2 Collecting duct acid secretion | 25.6534 | 108.1045 | 91.8192 | 125.6651 | 154.2024 | 130.6536 | 102.7965 | 70.7198 |
| 5.5.3 Endocrine and other factor-regulated calcium reabsorption | 37.7918 | 98.3413 | 128.8146 | 110.3328 | 163.0553 | 190.1646 | 59.1739 | 27.6770 |
| 5.5.4 Proximal tubule bicarbonate reclamation | 32.7189 | 194.4290 | 277.0443 | 204.6749 | 322.9102 | 401.5919 | 226.5285 | 67.8257 |
| 5.5.5 Vasopressin-regulated water reabsorption | 11.7856 | 36.0524 | 24.1490 | 34.6915 | 46.9498 | 57.5161 | 44.7145 | 23.6586 |
| **5.6 Nervous System** |  |  |  |  |  |  |  |  |
| 5.6.1 Glutamatergic synapse | 25.5654 | 105.1728 | 89.9110 | 102.5245 | 160.1590 | 145.7472 | 18.0896 | 24.9511 |
| 5.6.2 Long-term depression | 35.3774 | 87.4887 | 86.5246 | 95.8708 | 168.7396 | 141.5792 | 33.1305 | 24.1751 |
| 5.6.3 Long-term potentiation | 33.7597 | 93.4093 | 79.8990 | 85.9348 | 145.9800 | 128.7391 | 28.7175 | 30.0028 |
| 5.6.4 Neurotrophin signaling pathway | 19.1353 | 77.8615 | 61.2749 | 72.8549 | 118.9629 | 93.9321 | 23.6357 | 24.5815 |
| **5.8 Development** |  |  |  |  |  |  |  |  |
| 5.8.1 Axon guidance | 37.3980 | 116.9966 | 75.2288 | 105.8081 | 167.6733 | 164.9784 | 40.1506 | 52.5687 |
| 5.8.2 Dorso-ventral axis formation | 99.3535 | 97.1541 | 63.2063 | 98.8841 | 143.8990 | 123.7430 | 69.2347 | 99.3535 |
| 5.8.3 Osteoclast differentiation | 17.0302 | 65.7925 | 69.0536 | 86.8077 | 147.8630 | 106.1810 | 31.8426 | 30.1690 |

**Table S8(c)** The detailed subdivided results of decision coefficient for the other KEGG subcategory pathways. The direct determination factor has been marked using the red frame.

| **2. Genetic information processing** | | | | |
| --- | --- | --- | --- | --- |
|  | **Subdivision of decision coefficient** | | | |
|  | 2.1 | 2.2 | 2.3 | 2.4 |
| **the direct determination factor and indirect**  **determination factor** | 0.012 | 0.060 | 0.078 | 0.054 |
|  | 0.060 | 0.080 | 0.197 | 0.130 |
|  | 0.078 | 0.197 | 0.134 | 0.183 |
|  | 0.054 | 0.130 | 0.183 | 0.072 |
| **Decision coefficient** | 0.204 | 0.468 | 0.592 | 0.438 |

| **4. Cellular processes** | | | | |
| --- | --- | --- | --- | --- |
|  | **Subdivision of decision coefficient** | | | |
|  | 4.1 | 4.2 | 4.3 | 4.4 |
| **the direct determination factor and indirect**  **determination factor** | 0.136 | 0.047 | 0.197 | 0.213 |
|  | 0.047 | 0.005 | 0.037 | 0.040 |
|  | 0.197 | 0.037 | 0.076 | 0.161 |
|  | 0.213 | 0.040 | 0.161 | 0.088 |
| **Decision coefficient** | 0.593 | 0.129 | 0.471 | 0.502 |

| **5. Organismal systems** | | | | | | | | | |
| --- | --- | --- | --- | --- | --- | --- | --- | --- | --- |
|  | **Subdivision of decision coefficient** | | | | | | | | |
|  | 5.1 | 5.2 | 5.3 | 5.4 | 5.5 | 5.6 | 5.7 | 5.8 | 5.9 |
| **the direct determination factor and indirect**  **determination factor** | 0.067 | -1.766 | -1.753 | -1.735 | 2.166 | 1.438 | 0.867 | 0.204 | -0.064 |
|  | -1.766 | 12.226 | 24.292 | 24.013 | -30.142 | -19.613 | -12.409 | -2.744 | 0.865 |
|  | -1.753 | 24.292 | 13.603 | 23.795 | -33.724 | -19.188 | -11.783 | -2.760 | 0.933 |
|  | -1.735 | 24.013 | 23.795 | 12.391 | -30.523 | -19.727 | -11.788 | -2.655 | 0.754 |
|  | 2.166 | -30.142 | -33.724 | -30.523 | 21.408 | 23.969 | 14.459 | 3.364 | -1.151 |
|  | 1.438 | -19.613 | -19.188 | -19.727 | 23.969 | 8.148 | 9.566 | 2.229 | -0.601 |
|  | 0.867 | -12.409 | -11.783 | -11.788 | 14.459 | 9.566 | 3.399 | 1.354 | -0.493 |
|  | 0.204 | -2.744 | -2.760 | -2.655 | 3.364 | 2.229 | 1.354 | 0.159 | -0.095 |
|  | -0.064 | 0.865 | 0.933 | 0.754 | -1.151 | -0.601 | -0.493 | -0.095 | 0.050 |
| **Decision coefficient** | -0.578 | -5.278 | -6.587 | -5.477 | -30.175 | -13.780 | -6.829 | -0.945 | 0.197 |

**Table S8 (d)** The detailed subdivided results of decision coefficient for the other KEGG secondary pathways. The direct determination factor has been marked using the red frame.

| **2.1 Transcription** | | | |
| --- | --- | --- | --- |
|  | **Subdivision of decision coefficient** | | |
|  | 2.1.1 | 2.1.2 | 2.1.3 |
| **the direct determination factor and indirect**  **determination factor** | 0.062 | 0.136 | 0.198 |
|  | 0.136 | 0.113 | 0.284 |
|  | 0.198 | 0.284 | 0.207 |
| **Decision coefficient** | 0.396 | 0.533 | 0.689 |

| **2.2 Translation** | | | | | |
| --- | --- | --- | --- | --- | --- |
|  | **Subdivision of decision coefficient** | | | | |
|  | 2.2.1 | 2.2.2 | 2.2.3 | 2.2.4 | 2.2.5 |
| **the direct determination factor and indirect**  **determination factor** | 0.041 | 0.127 | -0.003 | 0.060 | 0.038 |
|  | 0.127 | 0.142 | 0.053 | 0.138 | 0.101 |
|  | -0.003 | 0.053 | 0.103 | 0.061 | 0.025 |
|  | 0.060 | 0.138 | 0.061 | 0.039 | 0.053 |
|  | 0.038 | 0.101 | 0.025 | 0.053 | 0.022 |
| **Decision coefficient** | 0.263 | 0.561 | 0.239 | 0.351 | 0.239 |

| **2.3 Folding, Sorting and Degradation** | | | | | | | |
| --- | --- | --- | --- | --- | --- | --- | --- |
|  | **Subdivision of decision coefficient** | | | | | | |
|  | 2.3.1 | 2.3.2 | 2.3.3 | 2.3.4 | 2.3.5 | 2.3.6 | 2.3.7 |
| **the direct determination factor and indirect**  **determination factor** | 0.038 | 0.000 | -0.015 | -0.026 | -0.130 | -0.033 | -0.161 |
|  | 0.000 | 0.000 | 0.000 | 0.000 | 0.000 | 0.000 | 0.000 |
|  | -0.015 | 0.000 | 0.003 | 0.008 | 0.038 | 0.013 | 0.061 |
|  | -0.026 | 0.000 | 0.008 | 0.007 | 0.059 | 0.016 | 0.085 |
|  | -0.130 | 0.000 | 0.038 | 0.059 | 0.143 | 0.072 | 0.378 |
|  | -0.033 | 0.000 | 0.013 | 0.016 | 0.072 | 0.018 | 0.117 |
|  | -0.161 | 0.000 | 0.061 | 0.085 | 0.378 | 0.117 | 0.310 |
| **Decision coefficient** | -0.327 | -0.001 | 0.108 | 0.149 | 0.559 | 0.203 | 0.790 |

| **2.4 Replication and Repair** | | | | | | |
| --- | --- | --- | --- | --- | --- | --- |
|  | **Subdivision of decision coefficient** | | | | | |
|  | 2.4.1 | 2.4.2 | 2.4.3 | 2.4.4 | 2.4.5 | 2.4.6 |
| **the direct determination factor and indirect**  **determination factor** | 0.017 | 0.070 | 0.083 | 0.009 | 0.022 | 0.005 |
|  | 0.070 | 0.116 | 0.230 | 0.022 | 0.071 | 0.015 |
|  | 0.083 | 0.230 | 0.145 | 0.027 | 0.101 | 0.017 |
|  | 0.009 | 0.022 | 0.027 | 0.002 | 0.007 | 0.002 |
|  | 0.022 | 0.071 | 0.101 | 0.007 | 0.036 | 0.005 |
|  | 0.005 | 0.015 | 0.017 | 0.002 | 0.005 | 0.001 |
| **Decision coefficient** | 0.206 | 0.522 | 0.603 | 0.068 | 0.242 | 0.043 |

| **4.1 Transport and Catabolism** | | | | | |
| --- | --- | --- | --- | --- | --- |
|  | **Subdivision of decision coefficient** | | | | |
|  | 4.1.1 | 4.1.2 | 4.1.3 | 4.1.4 | 4.1.5 |
| **the direct determination factor and indirect**  **determination factor** | 0.000 | -0.002 | -0.006 | -0.007 | -0.002 |
|  | -0.002 | 0.019 | 0.090 | 0.107 | 0.033 |
|  | -0.006 | 0.090 | 0.118 | 0.263 | 0.099 |
|  | -0.007 | 0.107 | 0.263 | 0.173 | 0.067 |
|  | -0.002 | 0.033 | 0.099 | 0.067 | 0.046 |
| **Decision coefficient** | -0.017 | 0.247 | 0.565 | 0.604 | 0.243 |

| **4.3 Cell Growth and Death** | | | | |
| --- | --- | --- | --- | --- |
|  | **Subdivision of decision coefficient** | | | |
|  | 4.3.1 | 4.3.2 | 4.3.3 | 4.3.4 |
| **the direct determination factor and indirect**  **determination factor** | 0.048 | 0.109 | 0.113 | 0.110 |
|  | 0.109 | 0.070 | 0.140 | 0.134 |
|  | 0.113 | 0.140 | 0.074 | 0.129 |
|  | 0.110 | 0.134 | 0.129 | 0.074 |
| **Decision coefficient** | 0.380 | 0.452 | 0.456 | 0.447 |

| **4.4 Cell Communication** | | | | |
| --- | --- | --- | --- | --- |
|  | **Subdivision of decision coefficient** | | | |
|  | 4.4.1 | 4.4.2 | 4.4.3 | 4.4.4 |
| **the direct determination factor and indirect**  **determination factor** | 0.022 | 0.072 | 0.090 | 0.088 |
|  | 0.072 | 0.060 | 0.145 | 0.144 |
|  | 0.090 | 0.145 | 0.097 | 0.188 |
|  | 0.088 | 0.144 | 0.188 | 0.095 |
| **Decision coefficient** | 0.272 | 0.420 | 0.519 | 0.515 |

| **5.2 Endocrine System** | | | | | | |
| --- | --- | --- | --- | --- | --- | --- |
|  | **Subdivision of decision coefficient** | | | | | |
|  | 5.2.1 | 5.2.2 | 5.2.3 | 5.2.4 | 5.2.5 | 5.2.6 |
| **the direct determination factor and indirect**  **determination factor** | 0.003 | 0.062 | 0.087 | -0.021 | 0.023 | -0.042 |
|  | 0.062 | 0.300 | 0.825 | -0.206 | 0.195 | -0.399 |
|  | 0.087 | 0.825 | 0.597 | -0.292 | 0.276 | -0.569 |
|  | -0.021 | -0.206 | -0.292 | 0.039 | -0.062 | 0.140 |
|  | 0.023 | 0.195 | 0.276 | -0.062 | 0.040 | -0.133 |
|  | -0.042 | -0.399 | -0.569 | 0.140 | -0.133 | 0.137 |
| **Decision coefficient** | 0.113 | 0.778 | 0.924 | -0.402 | 0.337 | -0.867 |

| **5.1 Immune System** | | | | | | | | | | | | | | | |
| --- | --- | --- | --- | --- | --- | --- | --- | --- | --- | --- | --- | --- | --- | --- | --- |
|  | **Subdivision of decision coefficient** | | | | | | | | | | | | | | |
|  | 5.1.1 | 5.1.2 | 5.1.3 | 5.1.4 | 5.1.5 | 5.1.6 | 5.1.7 | 5.1.8 | 5.1.9 | 5.1.10 | 5.1.11 | 5.1.12 | 5.1.13 | 5.1.14 | 5.1.15 |
| **the direct determination factor and indirect determination factor** | 0.404 | 3.286 | -6.178 | -0.116 | -0.966 | -4.978 | -0.902 | 3.032 | -6.611 | 3.902 | 0.038 | -2.440 | -1.019 | 1.376 | 9.543 |
|  | 3.286 | 7.347 | -27.270 | -0.493 | -4.569 | -21.748 | -3.898 | 13.744 | -29.068 | 17.103 | 0.166 | -10.823 | -4.766 | 6.265 | 42.015 |
|  | -6.178 | -27.270 | 26.373 | 0.974 | 8.280 | 40.837 | 7.326 | -26.815 | 56.334 | -32.668 | -0.326 | 20.792 | 8.650 | -11.768 | -80.711 |
|  | -0.116 | -0.493 | 0.974 | 0.010 | 0.148 | 0.759 | 0.133 | -0.515 | 1.043 | -0.595 | -0.006 | 0.371 | 0.154 | -0.209 | -1.479 |
|  | -0.966 | -4.569 | 8.280 | 0.148 | 0.882 | 6.817 | 1.142 | -4.513 | 9.074 | -5.155 | -0.050 | 3.398 | 1.681 | -2.044 | -13.324 |
|  | -4.978 | -21.748 | 40.837 | 0.759 | 6.817 | 16.409 | 5.805 | -20.840 | 43.777 | -25.362 | -0.250 | 16.216 | 7.123 | -9.349 | -63.556 |
|  | -0.902 | -3.898 | 7.326 | 0.133 | 1.142 | 5.805 | 0.528 | -3.600 | 7.790 | -4.594 | -0.045 | 2.906 | 1.217 | -1.653 | -11.248 |
|  | 3.032 | 13.744 | -26.815 | -0.515 | -4.513 | -20.840 | -3.600 | 7.210 | -28.848 | 16.403 | 0.163 | -10.445 | -4.607 | 5.995 | 41.222 |
|  | -6.611 | -29.068 | 56.334 | 1.043 | 9.074 | 43.777 | 7.790 | -28.848 | 30.235 | -34.792 | -0.350 | 22.353 | 9.339 | -12.662 | -86.908 |
|  | 3.902 | 17.103 | -32.668 | -0.595 | -5.155 | -25.362 | -4.594 | 16.403 | -34.792 | 10.242 | 0.200 | -12.854 | -5.387 | 7.283 | 49.696 |
|  | 0.038 | 0.166 | -0.326 | -0.006 | -0.050 | -0.250 | -0.045 | 0.163 | -0.350 | 0.200 | 0.001 | -0.131 | -0.052 | 0.073 | 0.505 |
|  | -2.440 | -10.823 | 20.792 | 0.371 | 3.398 | 16.216 | 2.906 | -10.445 | 22.353 | -12.854 | -0.131 | 4.208 | 3.487 | -4.778 | -32.417 |
|  | -1.019 | -4.766 | 8.650 | 0.154 | 1.681 | 7.123 | 1.217 | -4.607 | 9.339 | -5.387 | -0.052 | 3.487 | 0.859 | -2.089 | -13.712 |
|  | 1.376 | 6.265 | -11.768 | -0.209 | -2.044 | -9.349 | -1.653 | 5.995 | -12.662 | 7.283 | 0.073 | -4.778 | -2.089 | 1.383 | 18.478 |
|  | 9.543 | 42.015 | -80.711 | -1.479 | -13.324 | -63.556 | -11.248 | 41.222 | -86.908 | 49.696 | 0.505 | -32.417 | -13.712 | 18.478 | 63.051 |
| **DC** | -1.630 | -12.710 | -16.171 | 0.179 | 0.798 | -8.342 | 0.905 | -12.414 | -19.293 | -16.577 | -0.064 | -0.158 | 0.879 | -3.698 | -78.846 |

| **5.3 Circulatory System** | | |
| --- | --- | --- |
|  | **Subdivision of decision coefficient** | |
|  | 5.3.1 | 5.3.2 |
| **the direct determination factor and indirect determination factor** | 0.235 | 0.437 |
|  | 0.437 | 0.327 |
| **Decision coefficient** | 0.673 | 0.673 |

| **5.4 Digestive System** | | | | | | | | | |
| --- | --- | --- | --- | --- | --- | --- | --- | --- | --- |
|  | **Subdivision of decision coefficient** | | | | | | | | |
|  | 5.4.1 | 5.4.2 | 5.4.3 | 5.4.4 | 5.4.5 | 5.4.6 | 5.4.7 | 5.4.8 | 5.4.9 |
| **the direct determination factor and indirect determination factor** | 1.121 | -0.139 | 0.148 | 0.493 | -0.918 | 1.285 | 0.885 | -1.991 | 0.078 |
|  | -0.139 | 0.008 | -0.010 | -0.032 | 0.061 | -0.083 | -0.094 | 0.138 | -0.006 |
|  | 0.148 | -0.010 | 0.005 | 0.034 | -0.059 | 0.087 | 0.063 | -0.136 | 0.006 |
|  | 0.493 | -0.032 | 0.034 | 0.056 | -0.195 | 0.289 | 0.193 | -0.448 | 0.018 |
|  | -0.918 | 0.061 | -0.059 | -0.195 | 0.217 | -0.518 | -0.439 | 0.798 | -0.033 |
|  | 1.285 | -0.083 | 0.087 | 0.289 | -0.518 | 0.381 | 0.505 | -1.162 | 0.043 |
|  | 0.885 | -0.094 | 0.063 | 0.193 | -0.439 | 0.505 | 0.313 | -0.846 | 0.045 |
|  | -1.991 | 0.138 | -0.136 | -0.448 | 0.798 | -1.162 | -0.846 | 0.912 | -0.078 |
|  | 0.078 | -0.006 | 0.006 | 0.018 | -0.033 | 0.043 | 0.045 | -0.078 | 0.005 |
| **Decision coefficient** | 0.962 | -0.156 | 0.137 | 0.408 | -1.085 | 0.826 | 0.625 | -2.813 | 0.078 |

| **5.5 Excretory System** | | | | | |
| --- | --- | --- | --- | --- | --- |
|  | **Subdivision of decision coefficient** | | | | |
|  | 5.5.1 | 5.5.2 | 5.5.3 | 5.5.4 | 5.5.5 |
| **the direct determination factor and indirect**  **determination factor** | 0.664 | 1.554 | -1.007 | 0.027 | -0.835 |
|  | 1.554 | 2.505 | -1.146 | 0.037 | -2.626 |
|  | -1.007 | -1.146 | 0.427 | -0.022 | 0.644 |
|  | 0.027 | 0.037 | -0.022 | 0.000 | -0.021 |
|  | -0.835 | -2.626 | 0.644 | -0.021 | 0.751 |
| **Decision coefficient** | 0.403 | 0.325 | -1.103 | 0.022 | -2.088 |

| **5.6 Nervous System** | | | | |
| --- | --- | --- | --- | --- |
|  | **Subdivision of decision coefficient** | | | |
|  | 5.6.1 | 5.6.2 | 5.6.3 | 5.6.4 |
| **the direct determination factor and indirect**  **determination factor** | 0.470 | -0.336 | 0.394 | 0.059 |
|  | -0.336 | 0.151 | -0.239 | -0.074 |
|  | 0.394 | -0.239 | 0.234 | 0.227 |
|  | 0.059 | -0.074 | 0.227 | 0.112 |
| **Decision coefficient** | 0.589 | -0.497 | 0.617 | 0.324 |

| **5.8 Development** | | | |
| --- | --- | --- | --- |
|  | **Subdivision of decision coefficient** | | |
|  | 5.8.1 | 5.8.2 | 5.8.3 |
| **the direct determination factor and indirect**  **determination factor** | 0.380 | 0.810 | -0.870 |
|  | 0.810 | 0.540 | -0.693 |
|  | -0.870 | -0.693 | 0.830 |
| **Decision coefficient** | 0.320 | 0.657 | -0.732 |
