# Supplementary material for: A decision analysis model for KEGG pathway analysis
Source: BMC Bioinformatics. 2016 Oct 6;17:407. doi: 10.1186/s12859-016-1285-1 (PMC5053338; doi:10.1186/s12859-016-1285-1)
Supplement: Additional file 2: Table S1. — This file provide the detailed impact data of selected KEGG pathway categories, subcategories and the secondary pathways from− 15 to 300 vs.− 30d in bovine mammary tissue during lactation in Table S1 (a) and (b), respectively. The numbers colored in red color are the filled data by the average of all the other impact values in this pathway, which is the missing data originally. (DOCX 35 kb) [file 12859_2016_1285_MOESM2_ESM.docx]

**Table S1 (a)：** The detailed impact data of selected KEGG pathway categories and subcategories from -15 to 300 vs. -30d in bovine mammary tissue during lactation

| **The categories and subcategories** | **Impact data** | | | | | | | |
| --- | --- | --- | --- | --- | --- | --- | --- | --- |
|  | **-15vs-30** | **1vs-30** | **15vs-30** | **30vs-30** | **60vs-30** | **120vs-30** | **240vs-30** | **300vs-30** |
| **1. Metabolism** | 23.247 | 94.482 | 118.048 | 120.704 | 184.008 | 159.201 | 59.612 | 42.519 |
| 1.1 Carbohydrate Metabolism | 19.968 | 114.800 | 137.771 | 130.305 | 195.378 | 175.222 | 66.955 | 40.259 |
| 1.2 Energy Metabolism | 21.327 | 59.478 | 90.627 | 119.313 | 89.373 | 161.289 | 67.237 | 78.201 |
| 1.3 Lipid Metabolism | 31.957 | 77.396 | 93.415 | 113.233 | 195.801 | 173.038 | 58.885 | 38.757 |
| 1.4 Nucleotide Metabolism | 14.531 | 56.586 | 63.725 | 78.279 | 95.130 | 111.325 | 42.901 | 29.988 |
| 1.5 Amino Acid Metabolism | 20.380 | 60.075 | 81.121 | 70.021 | 107.043 | 95.820 | 39.656 | 25.198 |
| 1.6 Metabolism of Other Amino Acids | 21.528 | 50.006 | 99.907 | 62.168 | 219.393 | 157.885 | 29.694 | 29.514 |
| 1.7 Glycan Biosynthesis and Metabolism | 25.820 | 77.129 | 77.345 | 93.841 | 193.043 | 156.463 | 50.782 | 33.611 |
| 1.8 Metabolism of Cofactors and Vitamins | 23.131 | 96.368 | 81.516 | 112.827 | 152.915 | 137.105 | 95.926 | 90.819 |
| 1.9 Metabolism of Terpenoids and Polyketides | 87.328 | 63.168 | 109.477 | 90.912 | 93.967 | 79.118 | 87.328 | 87.328 |
| 1.10 Biosynthesis of Other Secondary Metabolites | 741.878 | 582.643 | 874.281 | 1515.113 | 778.046 | 530.191 | 170.992 | 741.878 |
| 1.11 Xenobiotics Biodegradation and Metabolism | 18.299 | 80.541 | 115.983 | 116.500 | 168.462 | 164.500 | 73.052 | 41.689 |
| **3. Environmental Information Processing** | 26.406 | 92.296 | 91.827 | 110.829 | 181.155 | 148.582 | 46.662 | 40.412 |
| 3.1 Membrane transport | 33.576 | 159.775 | 163.780 | 200.414 | 334.573 | 265.012 | 42.150 | 26.267 |
| 3.2 Signal Transduction | 25.168 | 85.627 | 82.073 | 100.538 | 164.229 | 136.302 | 37.781 | 35.373 |
| 3.3 Signaling Molecules and Interaction | 28.555 | 94.256 | 103.607 | 118.699 | 192.077 | 154.795 | 80.732 | 63.604 |

**Table S1 (b)：** The detailed impact data of the selected secondary KEGG pathways from -15 to 300 vs. -30d in bovine mammary tissue during lactation

| **The secondary pathways** | **Impact data** | | | | | | | |
| --- | --- | --- | --- | --- | --- | --- | --- | --- |
|  | **-15vs-30** | **1vs-30** | **15vs-30** | **30vs-30** | **60vs-30** | **120vs-30** | **240vs-30** | **300vs-30** |
| **1.1 Carbohydrate Metabolism** |  |  |  |  |  |  |  |  |
| 1.1.1 Amino sugar and nucleotide sugar metabolism | 26.906 | 69.477 | 89.332 | 54.550 | 140.266 | 89.478 | 20.357 | 24.924 |
| 1.1.2 Ascorbate and aldarate metabolism | 21.974 | 176.164 | 203.399 | 192.854 | 298.971 | 372.099 | 61.783 | 42.745 |
| 1.1.3 Butanoate metabolism | 93.706 | 46.441 | 52.979 | 126.287 | 146.336 | 113.867 | 104.301 | 65.729 |
| 1.1.4 Citrate cycle (TCA cycle) | 7.127 | 189.771 | 229.294 | 216.473 | 325.272 | 251.654 | 131.100 | 53.147 |
| 1.1.5 Fructose and mannose metabolism | 50.282 | 81.574 | 50.299 | 86.549 | 140.712 | 186.826 | 37.094 | 31.617 |
| 1.1.6 Galactose metabolism | 11.037 | 391.477 | 422.062 | 468.176 | 523.290 | 473.040 | 295.464 | 188.855 |
| 1.1.7 Glycolysis / Gluconeogenesis | 25.879 | 83.309 | 129.277 | 101.741 | 147.689 | 120.436 | 52.203 | 23.158 |
| 1.1.8 Glyoxylate and dicarboxylate metabolism | 13.578 | 65.701 | 74.502 | 104.030 | 136.130 | 76.090 | 31.086 | 15.643 |
| 1.1.9 Inositol phosphate metabolism | 26.242 | 91.914 | 74.441 | 109.235 | 165.464 | 126.721 | 29.402 | 18.716 |
| 1.1.10 Pentose and glucuronate interconversions | 9.270 | 140.108 | 151.516 | 98.463 | 207.241 | 214.119 | 50.307 | 8.910 |
| 1.1.11 Pentose phosphate pathway | 20.165 | 5.950 | 27.601 | 36.081 | 40.792 | 53.995 | 21.665 | 14.561 |
| 1.1.12 Propanoate metabolism | 11.911 | 70.799 | 99.934 | 54.894 | 133.662 | 86.923 | 11.874 | 4.633 |
| 1.1.13 Pyruvate metabolism | 15.248 | 105.098 | 119.504 | 92.557 | 163.199 | 164.808 | 72.873 | 30.728 |
| 1.1.14 Starch and sucrose metabolism | 95.579 | 89.416 | 94.489 | 82.376 | 166.275 | 123.053 | 17.865 | 95.579 |
| **1.2 Energy Metabolism** |  |  |  |  |  |  |  |  |
| 1.2.1 Nitrogen metabolism | 43.831 | 31.606 | 59.065 | 34.977 | 31.715 | 90.813 | 14.808 | 43.831 |
| 1.2.2 Oxidative phosphorylation | 21.327 | 76.388 | 68.609 | 87.301 | 119.563 | 109.980 | 62.218 | 45.015 |
| 1.2.3 Sulfur metabolism | 155.185 | 70.439 | 144.207 | 235.661 | 116.842 | 283.073 | 124.687 | 111.388 |
| **1.3 Lipid Metabolism** |  |  |  |  |  |  |  |  |
| 1.3.1 alpha-Linolenic acid metabolism | 28.519 | 61.269 | 41.135 | 46.450 | 74.019 | 107.121 | 53.836 | 18.336 |
| 1.3.2 Arachidonic acid metabolism | 14.415 | 111.211 | 118.180 | 116.674 | 212.650 | 175.410 | 48.195 | 25.141 |
| 1.3.3 Biosynthesis of unsaturated fatty acids | 223.708 | 80.685 | 216.720 | 267.595 | 512.720 | 327.201 | 92.313 | 68.722 |
| 1.3.4 Ether lipid metabolism | 40.809 | 111.247 | 84.470 | 127.891 | 188.719 | 224.666 | 17.852 | 31.152 |
| 1.3.5 Fatty acid elongation in mitochondria | 69.617 | 67.591 | 30.904 | 68.091 | 132.765 | 96.829 | 69.617 | 21.518 |
| 1.3.6 Fatty acid metabolism | 18.114 | 50.240 | 96.757 | 67.170 | 107.519 | 100.428 | 43.650 | 17.310 |
| 1.3.7 Glycerolipid metabolism | 36.979 | 93.721 | 135.282 | 103.389 | 165.748 | 148.192 | 24.842 | 34.447 |
| 1.3.8 Glycerophospholipid metabolism | 33.471 | 115.137 | 99.302 | 103.964 | 171.564 | 182.521 | 18.349 | 30.134 |
| 1.3.9 Primary bile acid biosynthesis | 78.100 | 67.662 | 117.560 | 55.250 | 92.055 | 68.356 | 67.713 | 78.100 |
| 1.3.10 Sphingolipid metabolism | 20.696 | 81.179 | 56.643 | 84.564 | 175.663 | 115.058 | 24.571 | 18.139 |
| 1.3.11 Steroid biosynthesis | 64.770 | 39.509 | 43.432 | 123.440 | 317.843 | 267.755 | 75.545 | 35.757 |
| 1.3.12 Steroid hormone biosynthesis | 29.842 | 61.457 | 41.564 | 68.168 | 94.981 | 172.818 | 91.169 | 18.538 |
| 1.3.13 Synthesis and degradation of ketone bodies | 215.092 | 116.102 | 132.447 | 315.719 | 318.805 | 263.142 | 143.537 | 145.893 |
| **1.4 Nucleotide Metabolism** |  |  |  |  |  |  |  |  |
| 1.4.1 Purine metabolism | 17.639 | 65.860 | 63.652 | 90.633 | 113.867 | 124.005 | 35.844 | 25.947 |
| 1.4.2 Pyrimidine metabolism | 11.424 | 47.313 | 63.798 | 65.925 | 76.393 | 98.646 | 49.957 | 34.029 |
| **1.5 Amino Acid Metabolism** |  |  |  |  |  |  |  |  |
| 1.5.1 Alanine, aspartate and glutamate metabolism | 23.439 | 12.654 | 4.867 | 22.982 | 50.942 | 51.363 | 7.929 | 13.335 |
| 1.5.2 Arginine and proline metabolism | 23.645 | 57.972 | 93.865 | 35.567 | 96.444 | 104.747 | 18.452 | 40.559 |
| 1.5.3 Cysteine and methionine metabolism | 29.877 | 86.624 | 106.239 | 98.570 | 166.901 | 76.231 | 6.386 | 50.805 |
| 1.5.4 Glycine, serine and threonine metabolism | 46.147 | 108.385 | 84.368 | 123.108 | 175.150 | 122.350 | 46.463 | 8.233 |
| 1.5.5 Histidine metabolism | 16.866 | 91.231 | 142.921 | 75.860 | 145.458 | 121.555 | 60.239 | 20.808 |
| 1.5.6 Lysine degradation | 16.212 | 52.131 | 72.496 | 47.985 | 77.800 | 99.948 | 5.216 | 4.455 |
| 1.5.7 Phenylalanine metabolism | 7.585 | 30.417 | 39.498 | 22.178 | 34.176 | 16.522 | 53.403 | 12.201 |
| 1.5.8 Tryptophan metabolism | 14.137 | 53.726 | 116.790 | 84.603 | 103.502 | 115.650 | 53.039 | 34.724 |
| 1.5.9 Tyrosine metabolism | 3.338 | 20.405 | 63.115 | 59.057 | 55.466 | 54.103 | 65.786 | 28.949 |
| 1.5.10 Valine, leucine and isoleucine biosynthesis | 42.445 | 69.410 | 70.419 | 80.671 | 99.930 | 117.195 | 35.724 | 14.083 |
| 1.5.11 Valine, leucine and isoleucine degradation | 3.545 | 77.867 | 97.758 | 119.652 | 171.702 | 174.352 | 83.574 | 49.023 |
| **1.6 Metabolism of Other Amino Acids** |  |  |  |  |  |  |  |  |
| 1.6.1 beta-Alanine metabolism | 22.637 | 80.322 | 130.286 | 68.054 | 127.658 | 109.230 | 26.051 | 15.606 |
| 1.6.2 Glutathione metabolism | 14.525 | 80.055 | 129.344 | 111.174 | 245.959 | 210.402 | 65.025 | 44.254 |
| 1.6.3 Selenoamino acid metabolism | 27.422 | 30.782 | 40.091 | 52.841 | 79.335 | 115.057 | 9.729 | 12.756 |
| 1.6.4 Taurine and hypotaurine metabolism | 101.489 | 8.864 | 101.489 | 39.385 | 322.006 | 177.368 | 23.832 | 37.478 |
| **1.7 Glycan Biosynthesis and Metabolism** |  |  |  |  |  |  |  |  |
| 1.7.1 Glycosaminoglycan biosynthesis - chondroitin sulfate | 9.412 | 72.239 | 85.056 | 141.802 | 159.156 | 147.846 | 43.587 | 72.211 |
| 1.7.2 Glycosaminoglycan biosynthesis - heparan sulfate | 43.641 | 107.524 | 142.514 | 114.250 | 206.687 | 134.360 | 57.302 | 17.753 |
| 1.7.3 Glycosaminoglycan biosynthesis - keratan sulfate | 22.852 | 60.785 | 27.042 | 41.947 | 124.906 | 87.501 | 12.656 | 53.956 |
| 1.7.4 Glycosaminoglycan degradation | 21.060 | 41.134 | 62.886 | 57.946 | 174.810 | 99.200 | 26.454 | 43.821 |
| 1.7.5 Glycosphingolipid biosynthesis - ganglio series | 37.063 | 59.103 | 32.894 | 67.241 | 241.103 | 186.627 | 91.018 | 13.098 |
| 1.7.6 Glycosphingolipid biosynthesis - globo series | 31.415 | 52.691 | 74.693 | 30.172 | 178.112 | 138.276 | 74.693 | 17.491 |
| 1.7.7 Glycosphingolipid biosynthesis - lacto and neolacto series | 13.421 | 46.659 | 63.471 | 94.732 | 167.412 | 127.505 | 43.843 | 17.498 |
| 1.7.8 Glycosylphosphatidylinositol(GPI)-anchor biosynthesis | 51.444 | 308.939 | 304.812 | 378.960 | 500.669 | 574.608 | 183.587 | 128.087 |
| 1.7.9 N-Glycan biosynthesis | 4.432 | 40.891 | 57.214 | 49.892 | 134.325 | 74.763 | 22.776 | 5.918 |
| 1.7.10 O-Glycan biosynthesis | 27.826 | 55.620 | 17.203 | 18.288 | 109.963 | 104.737 | 55.606 | 55.606 |
| 1.7.11 O-Mannosyl glycan biosynthesis | 32.497 | 62.601 | 45.033 | 71.428 | 146.928 | 160.515 | 16.052 | 7.139 |
| 1.7.12 Other glycan degradation | 14.783 | 17.365 | 12.671 | 59.434 | 172.447 | 41.615 | 47.345 | 13.098 |
| **1.8 Metabolism of Cofactors and Vitamins** |  |  |  |  |  |  |  |  |
| 1.8.1 Folate biosynthesis | 37.677 | 5.744 | 34.986 | 48.601 | 125.492 | 47.777 | 64.953 | 154.391 |
| 1.8.2 Nicotinate and nicotinamide metabolism | 23.751 | 16.200 | 56.080 | 77.878 | 89.339 | 120.391 | 73.754 | 71.907 |
| 1.8.3 One carbon pool by folate | 56.575 | 28.066 | 35.723 | 16.590 | 125.076 | 27.476 | 56.575 | 106.521 |
| 1.8.4 Pantothenate and CoA biosynthesis | 21.192 | 60.811 | 82.724 | 63.624 | 141.003 | 145.394 | 90.304 | 26.698 |
| 1.8.5 Porphyrin and chlorophyll metabolism | 13.586 | 57.089 | 89.657 | 105.601 | 206.134 | 163.922 | 45.137 | 97.304 |
| 1.8.6 Retinol metabolism | 11.508 | 75.372 | 101.430 | 136.791 | 215.428 | 164.410 | 44.719 | 14.057 |
| 1.8.7 Riboflavin metabolism | 107.475 | 132.031 | 157.680 | 137.421 | 89.968 | 123.656 | 37.556 | 74.012 |
| 1.8.8 Vitamin B6 metabolism | 198.952 | 198.952 | 93.851 | 146.195 | 192.394 | 246.539 | 284.087 | 230.648 |
| **1.11 Xenobiotics Biodegradation and Metabolism** |  |  |  |  |  |  |  |  |
| 1.11.1 Drug metabolism - cytochrome P450 | 21.592 | 45.096 | 88.615 | 96.782 | 129.649 | 142.666 | 108.900 | 74.892 |
| 1.11.2 Drug metabolism - other enzymes | 9.014 | 151.141 | 183.235 | 173.901 | 250.564 | 210.413 | 41.822 | 28.847 |
| 1.11.3 Metabolism of xenobiotics by cytochrome P450 | 24.291 | 45.386 | 76.098 | 78.818 | 125.172 | 140.422 | 68.435 | 21.328 |
| **3.2 Signal Transduction** |  |  |  |  |  |  |  |  |
| 3.2.1 Calcium signaling pathway | 25.513 | 93.957 | 76.065 | 100.100 | 164.415 | 138.900 | 23.626 | 40.477 |
| 3.2.2 ErbB signaling pathway | 25.392 | 67.828 | 67.323 | 96.452 | 145.420 | 123.505 | 18.387 | 19.613 |
| 3.2.3 Hedgehog signaling pathway | 30.979 | 114.345 | 148.575 | 148.247 | 242.320 | 226.304 | 77.285 | 73.302 |
| 3.2.4 Jak-STAT signaling pathway | 35.582 | 104.889 | 105.666 | 135.951 | 219.897 | 167.184 | 33.231 | 25.526 |
| 3.2.5 MAPK signaling pathway | 27.752 | 72.625 | 74.173 | 77.000 | 144.019 | 121.368 | 28.686 | 29.455 |
| 3.2.6 mTOR signaling pathway | 18.535 | 64.631 | 35.180 | 64.378 | 113.625 | 64.344 | 14.703 | 13.852 |
| 3.2.7 Notch signaling pathway | 5.155 | 78.851 | 76.671 | 103.924 | 150.617 | 109.100 | 30.675 | 33.842 |
| 3.2.8 Phosphatidylinositol signaling system | 19.998 | 67.747 | 61.220 | 67.784 | 145.603 | 111.330 | 30.128 | 23.698 |
| 3.2.9 TGF-beta signaling pathway | 8.743 | 101.926 | 130.659 | 135.474 | 206.199 | 149.720 | 96.667 | 50.822 |
| 3.2.10 VEGF signaling pathway | 48.294 | 98.847 | 63.953 | 91.343 | 139.549 | 152.345 | 26.645 | 41.537 |
| 3.2.11 Wnt signaling pathway | 30.902 | 76.249 | 63.319 | 85.266 | 134.855 | 135.228 | 35.555 | 36.975 |
| **3.3 Signaling Molecules and Interaction** |  |  |  |  |  |  |  |  |
| 3.3.1 Cell adhesion molecules (CAMs) | 22.371 | 103.036 | 102.092 | 125.849 | 204.747 | 191.979 | 72.873 | 77.700 |
| 3.3.2 Cytokine-cytokine receptor interaction | 38.676 | 91.617 | 94.678 | 106.992 | 161.011 | 139.295 | 49.006 | 42.773 |
| 3.3.3 ECM-receptor interaction | 24.617 | 88.114 | 114.051 | 123.256 | 210.472 | 133.110 | 120.318 | 70.338 |
