# Supplementary material for: A decision analysis model for KEGG pathway analysis
Source: BMC Bioinformatics. 2016 Oct 6;17:407. doi: 10.1186/s12859-016-1285-1 (PMC5053338; doi:10.1186/s12859-016-1285-1)
Supplement: Additional file 4: Table S3. — The file gives the comparison results of the most impacted pathways identified according to different Decision Coefficient (DC) values. (DOCX 24 kb) [file 12859_2016_1285_MOESM4_ESM.docx]

**Table S3：** The comparison of the most impacted pathways identified according to different Decision Coefficient (DC) values

|  |  |  |  |
| --- | --- | --- | --- |
| 1 | 1.1.4 Citrate cycle (TCA cycle) | 1.1.4 Citrate cycle (TCA cycle) | 1.1.4 Citrate cycle (TCA cycle) |
| 2 | 1.1.7 Glycolysis / Gluconeogenesis | 1.1.7 Glycolysis / Gluconeogenesis | 1.1.10 Pentose and glucuronate interconversions |
| 3 | 1.1.10 Pentose and glucuronate interconversions | 1.1.10 Pentose and glucuronate interconversions | 1.1.12 Propanoate metabolism |
| 4 | 1.1.12 Propanoate metabolism | 1.1.12 Propanoate metabolism | 1.1.13 Pyruvate metabolism |
| 5 | 1.1.13 Pyruvate metabolism | 1.1.13 Pyruvate metabolism | 1.4.1 Purine metabolism |
| 6 | 1.2.2 Oxidative phosphorylation | 1.2.2 Oxidative phosphorylation | 1.4.2 Pyrimidine metabolism |
| 7 | 1.2.3 Sulfur metabolism | 1.2.3 Sulfur metabolism | 1.5.3 Cysteine and methionine metabolism |
| 8 | 1.3.2 Arachidonic acid metabolism | 1.3.2 Arachidonic acid metabolism | 1.5.4 Glycine, serine and threonine metabolism |
| 9 | 1.3.8 Glycerophospholipid metabolism | 1.3.8 Glycerophospholipid metabolism | 1.5.10 Valine, leucine and isoleucine biosynthesis |
| 10 | 1.3.13 Synthesis and degradation of ketone bodies | 1.3.13 Synthesis and degradation of ketone bodies | 1.6.2 Glutathione metabolism |
| 11 | 1.4.1 Purine metabolism | 1.4.1 Purine metabolism | 1.6.4 Taurine and hypotaurine metabolism |
| 12 | 1.4.2 Pyrimidine metabolism | 1.4.2 Pyrimidine metabolism | 1.8.5 Porphyrin and chlorophyll metabolism |
| 13 | 1.5.3 Cysteine and methionine metabolism | 1.5.3 Cysteine and methionine metabolism | 1.8.6 Retinol metabolism |
| 14 | 1.5.4 Glycine, serine and threonine metabolism | 1.5.4 Glycine, serine and threonine metabolism | 1.8.7 Riboflavin metabolism |
| 15 | 1.5.6 Lysine degradation | 1.5.6 Lysine degradation | 1.11.2 Drug metabolism - other enzymes |
| 16 | 1.5.10 Valine, leucine and isoleucine biosynthesis | 1.5.10 Valine, leucine and isoleucine biosynthesis | 3.2.1 Calcium signaling pathway |
| 17 | 1.6.2 Glutathione metabolism | 1.6.2 Glutathione metabolism | 3.2.4 Jak-STAT signaling pathway |
| 18 | 1.6.4 Taurine and hypotaurine metabolism | 1.6.4 Taurine and hypotaurine metabolism | 3.2.10 VEGF signaling pathway |
| 19 | 1.7.1 Glycosaminoglycan biosynthesis - chondroitin sulfate | 1.7.1 Glycosaminoglycan biosynthesis - chondroitin sulfate | 3.3.1 Cell adhesion molecules (CAMs) |
| 20 | 1.7.5 Glycosphingolipid biosynthesis - ganglio series | 1.7.5 Glycosphingolipid biosynthesis - ganglio series |  |
| 21 | 1.7.8Glycosylphosphatidylinositol(GPI)-anchor biosynthesis | 1.8.5 Porphyrin and chlorophyll metabolism |  |
| 22 | 1.8.2 Nicotinate and nicotinamide metabolism | 1.8.6 Retinol metabolism |  |
| 23 | 1.8.4 Pantothenate and CoA biosynthesis | 1.8.7 Riboflavin metabolism |  |
| 24 | 1.8.5 Porphyrin and chlorophyll metabolism | 1.11.2 Drug metabolism - other enzymes |  |
| 25 | 1.8.6 Retinol metabolism | 3.2.1 Calcium signaling pathway |  |
| 26 | 1.8.7 Riboflavin metabolism | 3.2.4 Jak-STAT signaling pathway |  |
| 27 | 1.11.1 Drug metabolism - cytochrome P450 | 3.2.10 VEGF signaling pathway |  |
| 28 | 1.11.2 Drug metabolism - other enzymes | 3.3.1 Cell adhesion molecules (CAMs) |  |
| 29 | 1.11.3 Metabolism of xenobiotics by cytochrome P450 | 3.3.3 ECM-receptor interaction |  |
| 30 | 3.2.1 Calcium signaling pathway |  |  |
| 31 | 3.2.2 ErbB signaling pathway |  |  |
| 32 | 3.2.3 Hedgehog signaling pathway |  |  |
| 33 | 3.2.4 Jak-STAT signaling pathway |  |  |
| 34 | 3.2.5 MAPK signaling pathway |  |  |
| 35 | 3.2.7 Notch signaling pathway |  |  |
| 36 | 3.2.9 TGF-beta signaling pathway |  |  |
| 37 | 3.2.10 VEGF signaling pathway |  |  |
| 38 | 3.3.1 Cell adhesion molecules (CAMs) |  |  |
| 39 | 3.3.3 ECM-receptor interaction |  |  |
