# Supplementary material for: A decision analysis model for KEGG pathway analysis
Source: BMC Bioinformatics. 2016 Oct 6;17:407. doi: 10.1186/s12859-016-1285-1 (PMC5053338; doi:10.1186/s12859-016-1285-1)
Supplement: Additional file 5: Table S4. — This file gives the comparison of the most impacted pathway subcategories and the most impacted secondary pathways (DC value ≥ 0.4) under decision analysis model and DIA method in Table S4 (a) and (b), respectively. In ‘Group’ column, ‘a’ showed that the pathway was the most impacted pathway under both decision analysis model and DIA method; ‘b’ showed that the pathway was the most impacted pathways only under decision analysis model, and was not under DIA method. ‘RankDIA’ was the order of mean impact value for the pathway. The sign “+” and “−” represent the up-regulating and down-regulating impact direction, respectively. (DOCX 30 kb) [file 12859_2016_1285_MOESM5_ESM.docx]

**Table S4 (a)：** The comparison of the most impacted pathway subcategories (DC value$\geq0.4$) under decision analysis model and DIA method

| **KEGG**  **pathway category** | **KEGG pathway sub-category** | **DIA**  **average value** | **DIA value**  **order** | **Decision coefficient** | **DIA impact direction** | **Group** |
| --- | --- | --- | --- | --- | --- | --- |
| **Metabolism;**  **Environmental Information Processing;** | 1.1 Carbohydrate Metabolism | 110.082 | 2 | -7.668 | + | a |
|  | 1.2 Energy Metabolism | 85.856 | 8 | -0.974 | - | a |
|  | 1.3 Lipid Metabolism | 97.810 | 4 | 0.940 | + | a |
|  | 1.4 Nucleotide Metabolism | 61.558 | 11 | 0.881 | - | b |
|  | 1.5 Amino Acid Metabolism | 62.414 | 10 | -0.509 | + | b |
|  | 1.6 Metabolism of Other Amino Acids | 83.762 | 9 | 0.727 | + | b |
|  | 1.7 Glycan Biosynthesis and Metabolism | 88.504 | 6 | -7.305 | + | a |
|  | 1.8 Metabolism of Cofactors and Vitamins | 98.826 | 3 | 0.644 | + | a |
|  | 3.2 Signal Transduction | 83.386 | 3 | 0.894 | + | b |

**Table S4 (b)：**The comparison of the most impacted secondary pathways (DC value$\geq0.4$) under decision analysis model and DIA method

|  |  | **DIA Average impact value** | **Rank*_DIA_*** | **decision coefficient** | **DIA impact direction** | **Group** |
| --- | --- | --- | --- | --- | --- | --- |
| **1.1 Carbohydrate Metabolism** | 1.1.4 Citrate cycle (TCA cycle) | 175.480 | 2 | 0.760 | + | a |
|  | 1.1.7 Glycolysis / Gluconeogenesis | 85.462 | 8 | 0.469 | - | b |
|  | 1.1.10 Pentose and glucuronate interconversions | 109.992 | 4 | 0.537 | + | a |
|  | 1.1.12 Propanoate metabolism | 59.329 | 13 | -1.591 | + | b |
|  | 1.1.13 Pyruvate metabolism | 95.502 | 6 | 0.535 | - | b |
| **1.2 Energy Metabolism** | 1.2.2 Oxidative phosphorylation | 73.800 | 2 | 0.526 | + | a |
|  | 1.2.3 Sulfur metabolism | 155.185 | 1 | 0.453 | - | a |
| **1.3 Lipid Metabolism** | 1.3.2 Arachidonic acid metabolism | 102.735 | 5 | 0.571 | + | a |
|  | 1.3.5 Fatty acid elongation in mitochondria | 69.617 | 11 | -0.422 | - | b |
|  | 1.3.6 Fatty acid metabolism | 62.648 | 12 | -1.832 | - | b |
|  | 1.3.8 Glycerophospholipid metabolism | 94.305 | 6 | -1.210 | + | a |
|  | 1.3.12 Steroid hormone biosynthesis | 72.317 | 9 | 0.420 | - | b |
|  | 1.3.13 Synthesis and degradation of ketone bodies | 206.342 | 2 | 0.409 | + | a |
| **1.4 Nucleotide Metabolism** | 1.4.1 Purine metabolism | 67.181 | 1 | 0.834 | + | a |
|  | 1.4.2 Pyrimidine metabolism | 55.936 | 2 | 0.628 | - | a |
| **1.5 Amino Acid Metabolism** | 1.5.3 Cysteine and methionine metabolism | 77.704 | 4 | 0.514 | - | a |
|  | 1.5.4 Glycine, serine and threonine metabolism | 89.276 | 2 | -0.567 | - | a |
|  | 1.5.6 Lysine degradation | 47.030 | 8 | -0.423 | + | b |
|  | 1.5.10 Valine, leucine and isoleucine biosynthesis | 66.235 | 6 | 0.803 | + | b |
| **1.6 Metabolism of Other Amino Acids** | 1.6.2 Glutathione metabolism | 112.592 | 1 | 0.834 | + | a |
|  | 1.6.4 Taurine and hypotaurine metabolism | 101.489 | 2 | 0.536 | + | a |
| **1.7 Glycan Biosynthesis and Metabolism** | 1.7.1 Glycosaminoglycan biosynthesis - chondroitin sulfate | 91.414 | 3 | -1.460 | + | a |
|  | 1.7.4 Glycosaminoglycan degradation | 65.914 | 8 | -0.411 | + | b |
|  | 1.7.5 Glycosphingolipid biosynthesis - ganglio series | 91.018 | 4 | -3.439 | + | a |
|  | 1.7.10 O-Glycan biosynthesis | 55.606 | 9 | 0.499 | - | b |
| **1.8 Metabolism of Cofactors and Vitamins** | 1.8.2 Nicotinate and nicotinamide metabolism | 66.162 | 6 | -3.031 | + | b |
|  | 1.8.3 One carbon pool by folate | 56.575 | 8 | -3.395 | - | b |
|  | 1.8.5 Porphyrin and chlorophyll metabolism | 97.304 | 3 | -3.065 | - | a |
|  | 1.8.6 Retinol metabolism | 95.465 | 4 | -1.760 | + | a |
|  | 1.8.7 Riboflavin metabolism | 107.475 | 2 | -1.999 | + | a |
| **1.11 Xenobiotics Biodegradation and Metabolism** | 1.11.2 Drug metabolism - other enzymes | 131.117 | 1 | 0.747 | + | a |
|  | 1.11.3 Metabolism of xenobiotics by cytochrome P450 | 72.494 | 3 | 0.440 | - | b |
| **3.2 Signal Transduction** | 3.2.1 Calcium signaling pathway | 82.882 | 4 | -1.578 | + | a |
|  | 3.2.4 Jak-STAT signaling pathway | 103.491 | 3 | 0.658 | + | a |
|  | 3.2.7 Notch signaling pathway | 73.605 | 7 | 0.903 | - | a |
|  | 3.2.8 Phosphatidylinositol signaling system | 65.939 | 10 | 0.811 | + | b |
|  | 3.2.10 VEGF signaling pathway | 82.814 | 5 | 0.582 | + | a |
| **3.3 Signaling Molecules and Interaction** | 3.3.1 Cell adhesion molecules (CAMs) | 112.581 | 1 | 0.621 | - | a |
|  | 3.3.2 Cytokine-cytokine receptor interaction | 90.506 | 3 | 0.480 | + | a |
|  | 3.3.3 ECM-receptor interaction | 110.535 | 2 | 0.535 | + | a |
