# Supplementary material for: A decision analysis model for KEGG pathway analysis
Source: BMC Bioinformatics. 2016 Oct 6;17:407. doi: 10.1186/s12859-016-1285-1 (PMC5053338; doi:10.1186/s12859-016-1285-1)
Supplement: Additional file 7: Table S6. — This file provides the detailed subdivided results of decision coefficient for the selected KEGG subcategory pathways and the selected KEGG secondary pathways in Table S6 (a) and (b), respectively. In order to distinguish between the direct and indirect determination factor clearly, the direct determination factor has been indicated in red box. (DOCX 59 kb) [file 12859_2016_1285_MOESM7_ESM.docx]

**Table S6 (a):** The detailed subdivided results of decision coefficient for the selected KEGG subcategory pathways

| **1.Metobolism** | | | | | | | | | | | |
| --- | --- | --- | --- | --- | --- | --- | --- | --- | --- | --- | --- |
|  | **Subdivision of the decision coefficient** | | | | | | | | | | |
|  | 1.1 | 1.2 | 1.3 | 1.4 | 1.5 | 1.6 | 1.7 | 1.8 | 1.9 | 1.1 | 1.11 |
| **the direct ditermination and indirect ditermination** | 3.794 | 1.394 | -3.961 | -2.820 | -8.578 | -1.983 | 6.961 | -1.821 | 0.095 | -0.134 | -0.614 |
|  | 1.394 | 0.251 | -0.765 | -0.653 | -1.545 | -0.323 | 1.238 | -0.418 | 0.016 | -0.041 | -0.127 |
|  | -3.961 | -0.765 | 1.145 | 1.540 | 4.503 | 1.162 | -4.053 | 1.022 | -0.044 | 0.058 | 0.332 |
|  | -2.820 | -0.653 | 1.540 | 0.573 | 3.161 | 0.727 | -2.662 | 0.736 | -0.005 | 0.047 | 0.238 |
|  | -8.578 | -1.545 | 4.503 | 3.161 | 4.897 | 2.304 | -7.891 | 1.976 | -0.181 | 0.149 | 0.696 |
|  | -1.983 | -0.323 | 1.162 | 0.727 | 2.304 | 0.324 | -2.092 | 0.488 | -0.056 | 0.012 | 0.165 |
|  | 6.961 | 1.238 | -4.053 | -2.662 | -7.891 | -2.092 | 3.638 | -1.829 | 0.030 | -0.066 | -0.578 |
|  | -1.821 | -0.418 | 1.022 | 0.736 | 1.976 | 0.488 | -1.829 | 0.310 | 0.016 | 0.008 | 0.156 |
|  | 0.095 | 0.016 | -0.044 | -0.005 | -0.181 | -0.056 | 0.030 | 0.016 | 0.053 | -0.027 | -0.012 |
|  | -0.134 | -0.041 | 0.058 | 0.047 | 0.149 | 0.012 | -0.066 | 0.008 | -0.027 | 0.031 | 0.009 |
|  | -0.614 | -0.127 | 0.332 | 0.238 | 0.696 | 0.165 | -0.578 | 0.156 | -0.012 | 0.009 | 0.026 |
| **decision coefficient** | -7.668 | -0.974 | 0.940 | 0.881 | -0.509 | 0.727 | -7.305 | 0.644 | -0.114 | 0.046 | 0.291 |

| **3. Environmental Information Processing** | | | |
| --- | --- | --- | --- |
|  | **Subdivision of the decision coefficient** | | |
|  | 3.1 | 3.2 | 3.3 |
| **the direct ditermination and indirect ditermination** | 0.020 | 0.188 | 0.051 |
|  | 0.188 | 0.456 | 0.249 |
|  | 0.051 | 0.249 | 0.036 |
| **decision coefficient** | 0.259 | 0.894 | 0.336 |

**Table S6 (b):** The detailed subdivided results of decision coefficient for the selected KEGG secondary pathways

| **1.1 Carbohydrate Metabolism** | | | | | | | | | | | | | | |
| --- | --- | --- | --- | --- | --- | --- | --- | --- | --- | --- | --- | --- | --- | --- |
|  | **Subdivision of decision coefficient** | | | | | | | | | | | | | |
|  | 1.1.1 | 1.1.2 | 1.1.3 | 1.1.4 | 1.1.5 | 1.1.6 | 1.1.7 | 1.1.8 | 1.1.9 | 1.1.10 | 1.1.11 | 1.1.12 | 1.1.13 | 1.1.14 |
| **the direct determination factor and indirect determination factor** | 0.000 | -0.002 | -0.001 | 0.006 | 0.000 | -0.001 | 0.003 | 0.003 | 0.002 | 0.004 | 0.001 | -0.008 | 0.004 | 0.002 |
|  | -0.002 | 0.030 | 0.022 | -0.164 | 0.016 | 0.038 | -0.088 | -0.062 | -0.045 | -0.112 | -0.037 | 0.191 | -0.109 | -0.040 |
|  | -0.001 | 0.022 | 0.026 | -0.069 | 0.009 | 0.014 | -0.032 | -0.038 | -0.025 | -0.031 | -0.032 | 0.054 | -0.038 | -0.019 |
|  | 0.006 | -0.164 | -0.069 | 0.277 | -0.038 | -0.131 | 0.289 | 0.219 | 0.137 | 0.330 | 0.090 | -0.616 | 0.333 | 0.098 |
|  | 0.000 | 0.016 | 0.009 | -0.038 | 0.003 | 0.008 | -0.020 | -0.015 | -0.012 | -0.029 | -0.011 | 0.044 | -0.028 | -0.012 |
|  | -0.001 | 0.038 | 0.014 | -0.131 | 0.008 | 0.017 | -0.066 | -0.051 | -0.031 | -0.075 | -0.020 | 0.134 | -0.078 | -0.017 |
|  | 0.003 | -0.088 | -0.032 | 0.289 | -0.020 | -0.066 | 0.080 | 0.116 | 0.072 | 0.178 | 0.051 | -0.346 | 0.175 | 0.058 |
|  | 0.003 | -0.062 | -0.038 | 0.219 | -0.015 | -0.051 | 0.116 | 0.050 | 0.060 | 0.122 | 0.036 | -0.250 | 0.122 | 0.049 |
|  | 0.002 | -0.045 | -0.025 | 0.137 | -0.012 | -0.031 | 0.072 | 0.060 | 0.020 | 0.086 | 0.026 | -0.161 | 0.083 | 0.037 |
|  | 0.004 | -0.112 | -0.031 | 0.330 | -0.029 | -0.075 | 0.178 | 0.122 | 0.086 | 0.112 | 0.059 | -0.399 | 0.217 | 0.074 |
|  | 0.001 | -0.037 | -0.032 | 0.090 | -0.011 | -0.020 | 0.051 | 0.036 | 0.026 | 0.059 | 0.019 | -0.094 | 0.061 | 0.024 |
|  | -0.008 | 0.191 | 0.054 | -0.616 | 0.044 | 0.134 | -0.346 | -0.250 | -0.161 | -0.399 | -0.094 | 0.404 | -0.378 | -0.165 |
|  | 0.004 | -0.109 | -0.038 | 0.333 | -0.028 | -0.078 | 0.175 | 0.122 | 0.083 | 0.217 | 0.061 | -0.378 | 0.108 | 0.064 |
|  | 0.002 | -0.040 | -0.019 | 0.098 | -0.012 | -0.017 | 0.058 | 0.049 | 0.037 | 0.074 | 0.024 | -0.165 | 0.064 | 0.034 |
| **decision coefficient** | 0.012 | -0.362 | -0.160 | 0.760 | -0.085 | -0.260 | 0.469 | 0.360 | 0.248 | 0.537 | 0.172 | -1.591 | 0.535 | 0.186 |

| **1.2 Energy Metabolism** | | | |
| --- | --- | --- | --- |
|  | **Subdivision of decision coefficient** | | |
|  | 1.2.1 | 1.2.2 | 1.2.3 |
| **the direct determination factor and indirect determination factor** | 0.042 | 0.055 | 0.116 |
|  | 0.055 | 0.310 | 0.160 |
|  | 0.116 | 0.160 | 0.178 |
| **decision coefficient** | 0.214 | 0.526 | 0.453 |

| **1.4 Nucleotide Metabolism** | | |
| --- | --- | --- |
|  | **Subdivision of decision coefficient** | |
|  | 1.4.1 | 1.4.2 |
| **the direct determination factor and indirect determination factor** | 0.372 | 0.462 |
|  | 0.462 | 0.167 |
| **decision coefficient** | 0.834 | 0.628 |

| **1.6 Metabolism of Other Amino Acids** | | | | |
| --- | --- | --- | --- | --- |
|  | **Subdivision of decision coefficient** | | | |
|  | 1.6.1 | 1.6.2 | 1.6.3 | 1.6.4 |
| **the direct determination factor and indirect determination factor** | 0.009 | 0.103 | -0.006 | 0.044 |
|  | 0.103 | 0.398 | -0.045 | 0.378 |
|  | -0.006 | -0.045 | 0.002 | -0.021 |
|  | 0.044 | 0.378 | -0.021 | 0.135 |
| **decision coefficient** | 0.150 | 0.834 | -0.070 | 0.536 |

| **1.11 Xenobiotics Biodegradation and Metabolism** | | | |
| --- | --- | --- | --- |
|  | **Subdivision of decision coefficient** | | |
|  | 1.11.1 | 1.11.2 | 1.11.3 |
| **the direct determination factor and indirect determination factor** | 0.063 | 0.178 | 0.118 |
|  | 0.178 | 0.319 | 0.250 |
|  | 0.118 | 0.250 | 0.071 |
| **decision coefficient** | 0.360 | 0.747 | 0.440 |

| **1.3 Lipid Metabolism** | | | | | | | | | | | | | |
| --- | --- | --- | --- | --- | --- | --- | --- | --- | --- | --- | --- | --- | --- |
|  | **Subdivision of decision coefficient** | | | | | | | | | | | | |
|  | 1.3.1 | 1.3.2 | 1.3.3 | 1.3.4 | 1.3.5 | 1.3.6 | 1.3.7 | 1.3.8 | 1.3.9 | 1.3.10 | 1.3.11 | 1.3.12 | 1.3.13 |
| **the direct determination factor and indirect determination factor** | 0.034 | 0.458 | 0.001 | 0.010 | -0.063 | -0.192 | 0.011 | -0.154 | -0.021 | 0.000 | -0.016 | 0.133 | 0.063 |
|  | 0.458 | 2.497 | 0.017 | 0.089 | -0.526 | -2.135 | 0.139 | -1.547 | 0.172 | 0.001 | -0.142 | 0.797 | 0.752 |
|  | 0.001 | 0.017 | 0.000 | 0.000 | -0.003 | -0.007 | 0.001 | -0.005 | 0.001 | 0.000 | -0.001 | 0.002 | 0.005 |
|  | 0.010 | 0.089 | 0.000 | 0.001 | -0.010 | -0.036 | 0.003 | -0.031 | -0.001 | 0.000 | -0.003 | 0.017 | 0.017 |
|  | -0.063 | -0.526 | -0.003 | -0.010 | 0.057 | 0.185 | -0.012 | 0.160 | 0.028 | 0.000 | 0.023 | -0.120 | -0.141 |
|  | -0.192 | -2.135 | -0.007 | -0.036 | 0.185 | 0.524 | -0.063 | 0.639 | -0.175 | -0.001 | 0.057 | -0.347 | -0.281 |
|  | 0.011 | 0.139 | 0.001 | 0.003 | -0.012 | -0.063 | 0.002 | -0.045 | 0.010 | 0.000 | -0.004 | 0.019 | 0.020 |
|  | -0.154 | -1.547 | -0.005 | -0.031 | 0.160 | 0.639 | -0.045 | 0.261 | -0.030 | 0.000 | 0.044 | -0.265 | -0.239 |
|  | -0.021 | 0.172 | 0.001 | -0.001 | 0.028 | -0.175 | 0.010 | -0.030 | 0.104 | 0.000 | 0.001 | -0.069 | -0.067 |
|  | 0.000 | 0.001 | 0.000 | 0.000 | 0.000 | -0.001 | 0.000 | 0.000 | 0.000 | 0.000 | 0.000 | 0.000 | 0.000 |
|  | -0.016 | -0.142 | -0.001 | -0.003 | 0.023 | 0.057 | -0.004 | 0.044 | 0.001 | 0.000 | 0.003 | -0.031 | -0.037 |
|  | 0.133 | 0.797 | 0.002 | 0.017 | -0.120 | -0.347 | 0.019 | -0.265 | -0.069 | 0.000 | -0.031 | 0.143 | 0.141 |
|  | 0.063 | 0.752 | 0.005 | 0.017 | -0.141 | -0.281 | 0.020 | -0.239 | -0.067 | 0.000 | -0.037 | 0.141 | 0.175 |
| **decision coefficient** | 0.265 | 0.571 | 0.012 | 0.056 | -0.422 | -1.832 | 0.081 | -1.210 | -0.046 | 0.001 | -0.105 | 0.420 | 0.409 |

| **1.5 Amino Acid Metabolism** | | | | | | | | | | | |
| --- | --- | --- | --- | --- | --- | --- | --- | --- | --- | --- | --- |
|  | **Subdivision of decision coefficient** | | | | | | | | | | |
|  | 1.5.1 | 1.5.2 | 1.5.3 | 1.5.4 | 1.5.5 | 1.5.6 | 1.5.7 | 1.5.8 | 1.5.9 | 1.5.10 | 1.5.11 |
| **the direct determination factor and indirect determination factor** | 0.001 | 0.005 | 0.011 | -0.010 | -0.003 | -0.007 | -0.004 | 0.005 | 0.000 | 0.030 | 0.007 |
|  | 0.005 | 0.029 | 0.103 | -0.060 | -0.039 | -0.064 | 0.005 | 0.057 | -0.006 | 0.192 | 0.045 |
|  | 0.011 | 0.103 | 0.173 | -0.189 | -0.085 | -0.124 | 0.015 | 0.113 | -0.012 | 0.412 | 0.099 |
|  | -0.010 | -0.060 | -0.189 | 0.075 | 0.058 | 0.091 | -0.023 | -0.077 | 0.010 | -0.362 | -0.079 |
|  | -0.003 | -0.039 | -0.085 | 0.058 | 0.018 | 0.048 | -0.029 | -0.049 | 0.008 | -0.157 | -0.039 |
|  | -0.007 | -0.064 | -0.124 | 0.091 | 0.048 | 0.042 | -0.003 | -0.071 | 0.008 | -0.283 | -0.059 |
|  | -0.004 | 0.005 | 0.015 | -0.023 | -0.029 | -0.003 | 0.059 | 0.032 | -0.015 | 0.013 | 0.024 |
|  | 0.005 | 0.057 | 0.113 | -0.077 | -0.049 | -0.071 | 0.032 | 0.039 | -0.014 | 0.234 | 0.062 |
|  | 0.000 | -0.006 | -0.012 | 0.010 | 0.008 | 0.008 | -0.015 | -0.014 | 0.002 | -0.028 | -0.012 |
|  | 0.030 | 0.192 | 0.412 | -0.362 | -0.157 | -0.283 | 0.013 | 0.234 | -0.028 | 0.533 | 0.221 |
|  | 0.007 | 0.045 | 0.099 | -0.079 | -0.039 | -0.059 | 0.024 | 0.062 | -0.012 | 0.221 | 0.031 |
| **decision coefficient** | 0.034 | 0.266 | 0.514 | -0.567 | -0.270 | -0.423 | 0.072 | 0.329 | -0.060 | 0.803 | 0.299 |

| **1.7 Glycan Biosynthesis and Metabolism** | | | | | | | | | | | | |
| --- | --- | --- | --- | --- | --- | --- | --- | --- | --- | --- | --- | --- |
|  | **Subdivision of decision coefficient** | | | | | | | | | | | |
|  | 1.7.1 | 1.7.2 | 1.7.3 | 1.7.4 | 1.7.5 | 1.7.6 | 1.7.7 | 1.7.8 | 1.7.9 | 1.7.10 | 1.7.11 | 1.7.12 |
| **the direct determination factor and indirect determination factor** | 0.376 | 0.071 | -0.075 | 0.197 | 0.989 | -0.024 | -2.013 | -0.205 | -0.100 | -0.329 | -0.219 | -0.127 |
|  | 0.071 | 0.006 | -0.008 | 0.026 | 0.129 | -0.004 | -0.245 | -0.023 | -0.014 | -0.034 | -0.027 | -0.017 |
|  | -0.075 | -0.008 | 0.007 | -0.029 | -0.151 | 0.004 | 0.235 | 0.022 | 0.013 | 0.069 | 0.030 | 0.019 |
|  | 0.197 | 0.026 | -0.029 | 0.039 | 0.397 | -0.011 | -0.661 | -0.056 | -0.037 | -0.147 | -0.072 | -0.056 |
|  | 0.989 | 0.129 | -0.151 | 0.397 | 1.316 | -0.067 | -3.760 | -0.331 | -0.196 | -1.007 | -0.446 | -0.310 |
|  | -0.024 | -0.004 | 0.004 | -0.011 | -0.067 | 0.001 | 0.098 | 0.009 | 0.006 | 0.026 | 0.011 | 0.008 |
|  | -2.013 | -0.245 | 0.235 | -0.661 | -3.760 | 0.098 | 3.267 | 0.607 | 0.342 | 1.218 | 0.725 | 0.480 |
|  | -0.205 | -0.023 | 0.022 | -0.056 | -0.331 | 0.009 | 0.607 | 0.033 | 0.031 | 0.115 | 0.074 | 0.033 |
|  | -0.100 | -0.014 | 0.013 | -0.037 | -0.196 | 0.006 | 0.342 | 0.031 | 0.010 | 0.064 | 0.037 | 0.027 |
|  | -0.329 | -0.034 | 0.069 | -0.147 | -1.007 | 0.026 | 1.218 | 0.115 | 0.064 | 0.259 | 0.164 | 0.101 |
|  | -0.219 | -0.027 | 0.030 | -0.072 | -0.446 | 0.011 | 0.725 | 0.074 | 0.037 | 0.164 | 0.048 | 0.045 |
|  | -0.127 | -0.017 | 0.019 | -0.056 | -0.310 | 0.008 | 0.480 | 0.033 | 0.027 | 0.101 | 0.045 | 0.026 |
| **decision coefficient** | -1.460 | -0.142 | 0.134 | -0.411 | -3.439 | 0.055 | 0.291 | 0.307 | 0.181 | 0.499 | 0.368 | 0.229 |

| **1.8 Metabolism of Cofactors and Vitamins** | | | | | | | | |
| --- | --- | --- | --- | --- | --- | --- | --- | --- |
|  | **Subdivision of decision coefficient** | | | | | | | |
|  | 1.8.1 | 1.8.2 | 1.8.3 | 1.8.4 | 1.8.5 | 1.8.6 | 1.8.7 | 1.8.8 |
| **the direct determination factor and indirect determination factor** | 0.079 | -0.284 | -0.822 | 0.005 | 0.746 | -0.023 | 0.468 | -0.066 |
|  | -0.284 | 1.320 | 0.595 | -0.353 | -5.007 | 1.026 | -0.587 | 0.260 |
|  | -0.822 | 0.595 | 2.759 | -0.042 | -3.535 | 0.113 | -2.817 | 0.355 |
|  | 0.005 | -0.353 | -0.042 | 0.045 | 0.949 | -0.266 | -0.011 | -0.024 |
|  | 0.746 | -5.007 | -3.535 | 0.949 | 8.115 | -3.667 | -0.817 | 0.152 |
|  | -0.023 | 1.026 | 0.113 | -0.266 | -3.667 | 0.534 | 0.672 | -0.150 |
|  | 0.468 | -0.587 | -2.817 | -0.011 | -0.817 | 0.672 | 2.099 | -1.006 |
|  | -0.066 | 0.260 | 0.355 | -0.024 | 0.152 | -0.150 | -1.006 | 0.185 |
| **decision coefficient** | 0.103 | -3.031 | -3.395 | 0.304 | -3.065 | -1.760 | -1.999 | -0.294 |

| **3.2 Signal Transduction** | | | | | | | | | | | |
| --- | --- | --- | --- | --- | --- | --- | --- | --- | --- | --- | --- |
|  | **Subdivision of decision coefficient** | | | | | | | | | | |
|  | 3.2.1 | 3.2.2 | 3.2.3 | 3.2.4 | 3.2.5 | 3.2.6 | 3.2.7 | 3.2.8 | 3.2.9 | 3.2.10 | 3.2.11 |
| **the direct determination factor and indirect determination factor** | 0.371 | 0.200 | -0.201 | -0.504 | 0.173 | 0.145 | -0.876 | -0.691 | 0.170 | -0.460 | 0.095 |
|  | 0.200 | 0.028 | -0.055 | -0.140 | 0.047 | 0.039 | -0.239 | -0.188 | 0.047 | -0.124 | 0.026 |
|  | -0.201 | -0.055 | 0.030 | 0.138 | -0.048 | -0.037 | 0.244 | 0.193 | -0.052 | 0.121 | -0.026 |
|  | -0.504 | -0.140 | 0.138 | 0.177 | -0.120 | -0.101 | 0.608 | 0.478 | -0.121 | 0.306 | -0.064 |
|  | 0.173 | 0.047 | -0.048 | -0.120 | 0.021 | 0.034 | -0.204 | -0.166 | 0.041 | -0.107 | 0.022 |
|  | 0.145 | 0.039 | -0.037 | -0.101 | 0.034 | 0.016 | -0.175 | -0.137 | 0.034 | -0.087 | 0.018 |
|  | -0.876 | -0.239 | 0.244 | 0.608 | -0.204 | -0.175 | 0.550 | 0.819 | -0.224 | 0.509 | -0.110 |
|  | -0.691 | -0.188 | 0.193 | 0.478 | -0.166 | -0.137 | 0.819 | 0.337 | -0.167 | 0.423 | -0.090 |
|  | 0.170 | 0.047 | -0.052 | -0.121 | 0.041 | 0.034 | -0.224 | -0.167 | 0.025 | -0.093 | 0.022 |
|  | -0.460 | -0.124 | 0.121 | 0.306 | -0.107 | -0.087 | 0.509 | 0.423 | -0.093 | 0.155 | -0.061 |
|  | 0.095 | 0.026 | -0.026 | -0.064 | 0.022 | 0.018 | -0.110 | -0.090 | 0.022 | -0.061 | 0.006 |
| **decision coefficient** | -1.578 | -0.359 | 0.307 | 0.658 | -0.307 | -0.252 | 0.903 | 0.811 | -0.319 | 0.582 | -0.162 |

| **3.3 Signaling Molecules and Interaction** | | | | |
| --- | --- | --- | --- | --- |
|  | **Subdivision of decision coefficient** | | | |
|  | 3.3.1 | 3.3.2 | 3.3.3 |  |
| **the direct determination factor and indirect determination factor** | 0.157 | 0.222 | 0.241 |  |
|  | 0.222 | 0.086 | 0.172 |  |
|  | 0.241 | 0.172 | 0.122 |  |
| **decision coefficient** | 0.621 | 0.480 | 0.535 |  |
