# Supplementary material for: A decision analysis model for KEGG pathway analysis
Source: BMC Bioinformatics. 2016 Oct 6;17:407. doi: 10.1186/s12859-016-1285-1 (PMC5053338; doi:10.1186/s12859-016-1285-1)
Supplement: Additional file 8: Figure S1. — The file gives the decision trees of selected pathway categories and subcategories plotted according to the decision percentage. (for (a) Metabolism and (b) Environmental Information Processing) The activated KEGG subcategory pathways were marked with red color, the inhibited KEGG subcategory pathways were marked with blue color. In the same way, the activated secondary KEGG pathways were marked with red circles; the inhibited secondary KEGG pathways were marked with blue circles. (DOCX 431 kb) [file 12859_2016_1285_MOESM8_ESM.docx]

**Figure S1:** The decision tree of selected pathway categories and subcategories

**1)**. Metabolism

**3)** Environmental Information Processing
