# Supplementary material for: A decision analysis model for KEGG pathway analysis
Source: BMC Bioinformatics. 2016 Oct 6;17:407. doi: 10.1186/s12859-016-1285-1 (PMC5053338; doi:10.1186/s12859-016-1285-1)
Supplement: Additional file 9: Table S7. — This file provides the decision analysis and path analysis results of the selected category pathways and the selected subcategory pathways in Table S7 (a) and (b). (DOCX 1278 kb) [file 12859_2016_1285_MOESM9_ESM.docx]

**Table S7 (a)** The decision analysis and path analysis results of the selected category pathways

| **3. Environmental Information Processing** | | | | | | | | |
| --- | --- | --- | --- | --- | --- | --- | --- | --- |
| $\begin{matrix} x_{j} \\ \text{to} \\ y \end{matrix}$ | $b_{j}^{*}$ | $x_{j}\leftrightarrow x_{k}\to y$ | $r_{jk}b_{k}^{*}$ | $2b_{j}^{*}r_{jk}b_{k}^{*}$ | $\sum_{k\neq j} r_{jk}b_{k}^{*}$ | $r_{jy}$ | $\sum_{j\neq k} r_{kj}b_{j}^{*}$ | $R_{(j)}$ |
| $\begin{matrix} x_{1} \\ \text{to} \\ y \end{matrix}$ | 0.140 | $x_{1}\leftrightarrow x_{2}\to y$ | 0.672 | 0.188 | 0.853 | 0.993 | 0.272 | 0.259 |
|  |  | $x_{1}\leftrightarrow x_{3}\to y$ | 0.181 | 0.051 |  |  |  |  |
| $\begin{matrix} x_{2} \\ \text{to} \\ y \end{matrix}$ | 0.676 | $x_{2}\leftrightarrow x_{1}\to y$ | 0.139 | 0.188 | 0.323 | 0.999 | 1.328 | 0.894 |
|  |  | $x_{2}\leftrightarrow x_{3}\to y$ | 0.184 | 0.249 |  |  |  |  |
| $\begin{matrix} x_{3} \\ \text{to} \\ y \end{matrix}$ | 0.190 | $x_{3}\leftrightarrow x_{1}\to y$ | 0.133 | 0.051 | 0.789 | 0.979 | 0.365 | 0.336 |
|  |  | $x_{3}\leftrightarrow x_{2}\to y$ | 0.656 | 0.249 |  |  |  |  |

| **1. Metabolism** | | | | | | | | |
| --- | --- | --- | --- | --- | --- | --- | --- | --- |
| $\begin{matrix} x_{j} \\ \text{to} \\ y \end{matrix}$ | $b_{j}^{*}$ | $x_{j}\leftrightarrow x_{k}\to y$ | $r_{jk}b_{k}^{*}$ | $2b_{j}^{*}r_{jk}b_{k}^{*}$ | $\sum_{k\neq j} r_{jk}b_{k}^{*}$ | $r_{jy}$ | $\sum_{j\neq k} r_{kj}b_{j}^{*}$ | $R_{(j)}$ |
| $\begin{matrix} x_{1} \\ \text{to} \\ y \end{matrix}$ | -1.948 | $x_{1}\leftrightarrow x_{2}\to y$ | -0.358 | 1.394 | 2.942 | 0.994 | -14.738 | -7.668 |
|  |  | $x_{1}\leftrightarrow x_{3}\to y$ | 1.017 | -3.961 |  |  |  |  |
|  |  | $x_{1}\leftrightarrow x_{4}\to y$ | 0.724 | -2.820 |  |  |  |  |
|  |  | $x_{1}\leftrightarrow x_{5}\to y$ | 2.202 | -8.578 |  |  |  |  |
|  |  | $x_{1}\leftrightarrow x_{6}\to y$ | 0.509 | -1.983 |  |  |  |  |
|  |  | $x_{1}\leftrightarrow x_{7}\to y$ | -1.787 | 6.961 |  |  |  |  |
|  |  | $x_{1}\leftrightarrow x_{8}\to y$ | 0.467 | -1.821 |  |  |  |  |
|  |  | $x_{1}\leftrightarrow x_{9}\to y$ | -0.024 | 0.095 |  |  |  |  |
|  |  | $x_{1}\leftrightarrow x_{10}\to y$ | 0.034 | -0.134 |  |  |  |  |
|  |  | $x_{1}\leftrightarrow x_{11}\to y$ | 0.158 | -0.614 |  |  |  |  |
| $\begin{matrix} x_{2} \\ \text{to} \\ y \end{matrix}$ | -0.501 | $x_{2}\leftrightarrow x_{1}\to y$ | -1.391 | 1.394 | 1.223 | 0.722 | -3.026 | -0.974 |
|  |  | $x_{2}\leftrightarrow x_{3}\to y$ | 0.763 | -0.765 |  |  |  |  |
|  |  | $x_{2}\leftrightarrow x_{4}\to y$ | 0.652 | -0.653 |  |  |  |  |
|  |  | $x_{2}\leftrightarrow x_{5}\to y$ | 1.542 | -1.545 |  |  |  |  |
|  |  | $x_{2}\leftrightarrow x_{6}\to y$ | 0.323 | -0.323 |  |  |  |  |
|  |  | $x_{2}\leftrightarrow x_{7}\to y$ | -1.235 | 1.238 |  |  |  |  |
|  |  | $x_{2}\leftrightarrow x_{8}\to y$ | 0.418 | -0.418 |  |  |  |  |
|  |  | $x_{2}\leftrightarrow x_{9}\to y$ | -0.016 | 0.016 |  |  |  |  |
|  |  | $x_{2}\leftrightarrow x_{10}\to y$ | 0.041 | -0.041 |  |  |  |  |
|  |  | $x_{2}\leftrightarrow x_{11}\to y$ | 0.127 | -0.127 |  |  |  |  |
| $\begin{matrix} x_{3} \\ \text{to} \\ y \end{matrix}$ | 1.070 | $x_{3}\leftrightarrow x_{1}\to y$ | -1.851 | -3.691 | -0.096 | 0.974 | 8.107 | 0.940 |
|  |  | $x_{3}\leftrightarrow x_{2}\to y$ | -0.357 | -0.765 |  |  |  |  |
|  |  | $x_{3}\leftrightarrow x_{4}\to y$ | 0.720 | 1.540 |  |  |  |  |
|  |  | $x_{3}\leftrightarrow x_{5}\to y$ | 2.104 | 4.503 |  |  |  |  |
|  |  | $x_{3}\leftrightarrow x_{6}\to y$ | 0.543 | 1.162 |  |  |  |  |
|  |  | $x_{3}\leftrightarrow x_{7}\to y$ | -1.894 | -4.053 |  |  |  |  |
|  |  | $x_{3}\leftrightarrow x_{8}\to y$ | 0.478 | 1.022 |  |  |  |  |
|  |  | $x_{3}\leftrightarrow x_{9}\to y$ | -0.020 | -0.044 |  |  |  |  |
|  |  | $x_{3}\leftrightarrow x_{10}\to y$ | 0.027 | 0.058 |  |  |  |  |
|  |  | $x_{3}\leftrightarrow x_{11}\to y$ | 0.155 | 0.332 |  |  |  |  |
| $\begin{matrix} x_{4} \\ \text{to} \\ y \end{matrix}$ | 0.757 | $x_{4}\leftrightarrow x_{1}\to y$ | -1.863 | -2.820 | 0.203 | 0.960 | 5.691 | 0.881 |
|  |  | $x_{4}\leftrightarrow x_{2}\to y$ | -0.432 | -0.653 |  |  |  |  |
|  |  | $x_{4}\leftrightarrow x_{3}\to y$ | 1.018 | 1.540 |  |  |  |  |
|  |  | $x_{4}\leftrightarrow x_{5}\to y$ | 2.088 | 3.161 |  |  |  |  |
|  |  | $x_{4}\leftrightarrow x_{6}\to y$ | 0.480 | 0.727 |  |  |  |  |
|  |  | $x_{4}\leftrightarrow x_{7}\to y$ | -1.759 | -2.662 |  |  |  |  |
|  |  | $x_{4}\leftrightarrow x_{8}\to y$ | 0.486 | 0.736 |  |  |  |  |
|  |  | $x_{4}\leftrightarrow x_{9}\to y$ | -0.003 | -0.005 |  |  |  |  |
|  |  | $x_{4}\leftrightarrow x_{10}\to y$ | 0.031 | 0.047 |  |  |  |  |
|  |  | $x_{4}\leftrightarrow x_{11}\to y$ | 0.157 | 0.238 |  |  |  |  |
| $\begin{matrix} x_{5} \\ \text{to} \\ y \end{matrix}$ | 2.213 | $x_{5}\leftrightarrow x_{1}\to y$ | -1.938 | -8.578 | -1.221 | 0.991 | 16.785 | -0.509 |
|  |  | $x_{5}\leftrightarrow x_{2}\to y$ | -0.349 | -1.545 |  |  |  |  |
|  |  | $x_{5}\leftrightarrow x_{3}\to y$ | 1.018 | 4.503 |  |  |  |  |
|  |  | $x_{5}\leftrightarrow x_{4}\to y$ | 0.714 | 3.161 |  |  |  |  |
|  |  | $x_{5}\leftrightarrow x_{6}\to y$ | 0.521 | 2.304 |  |  |  |  |
|  |  | $x_{5}\leftrightarrow x_{7}\to y$ | -1.783 | -7.891 |  |  |  |  |
|  |  | $x_{5}\leftrightarrow x_{8}\to y$ | 0.446 | 1.976 |  |  |  |  |
|  |  | $x_{5}\leftrightarrow x_{9}\to y$ | -0.041 | -0.181 |  |  |  |  |
|  |  | $x_{5}\leftrightarrow x_{10}\to y$ | 0.034 | 0.149 |  |  |  |  |
|  |  | $x_{5}\leftrightarrow x_{11}\to y$ | 0.157 | 0.696 |  |  |  |  |
| $\begin{matrix} x_{6} \\ \text{to} \\ y \end{matrix}$ | 0.569 | $x_{6}\leftrightarrow x_{1}\to y$ | -1.742 | -1.983 | 0.354 | 0.923 | 4.029 | 0.727 |
|  |  | $x_{6}\leftrightarrow x_{2}\to y$ | -0.284 | -0.323 |  |  |  |  |
|  |  | $x_{6}\leftrightarrow x_{3}\to y$ | 1.020 | 1.162 |  |  |  |  |
|  |  | $x_{6}\leftrightarrow x_{4}\to y$ | 0.638 | 0.727 |  |  |  |  |
|  |  | $x_{6}\leftrightarrow x_{5}\to y$ | 2.024 | 2.304 |  |  |  |  |
|  |  | $x_{6}\leftrightarrow x_{7}\to y$ | -1.837 | -2.092 |  |  |  |  |
|  |  | $x_{6}\leftrightarrow x_{8}\to y$ | 0.428 | 0.488 |  |  |  |  |
|  |  | $x_{6}\leftrightarrow x_{9}\to y$ | -0.049 | -0.056 |  |  |  |  |
|  |  | $x_{6}\leftrightarrow x_{10}\to y$ | 0.010 | 0.012 |  |  |  |  |
|  |  | $x_{6}\leftrightarrow x_{11}\to y$ | 0.145 | 0.165 |  |  |  |  |
| $\begin{matrix} x_{7} \\ \text{to} \\ y \end{matrix}$ | -1.907 | $x_{7}\leftrightarrow x_{1}\to y$ | -1.825 | 6.961 | 2.869 | 0.961 | -13.989 | -7.305 |
|  |  | $x_{7}\leftrightarrow x_{2}\to y$ | -0.324 | 1.238 |  |  |  |  |
|  |  | $x_{7}\leftrightarrow x_{3}\to y$ | 1.063 | -4.053 |  |  |  |  |
|  |  | $x_{7}\leftrightarrow x_{4}\to y$ | 0.698 | -2.662 |  |  |  |  |
|  |  | $x_{7}\leftrightarrow x_{5}\to y$ | 2.069 | -7.891 |  |  |  |  |
|  |  | $x_{7}\leftrightarrow x_{6}\to y$ | 0.548 | -2.092 |  |  |  |  |
|  |  | $x_{7}\leftrightarrow x_{8}\to y$ | 0.480 | -1.829 |  |  |  |  |
|  |  | $x_{7}\leftrightarrow x_{9}\to y$ | -0.008 | 0.030 |  |  |  |  |
|  |  | $x_{7}\leftrightarrow x_{10}\to y$ | 0.017 | -0.066 |  |  |  |  |
|  |  | $x_{7}\leftrightarrow x_{11}\to y$ | 0.151 | -0.578 |  |  |  |  |
| $\begin{matrix} x_{8} \\ \text{to} \\ y \end{matrix}$ | 0.557 | $x_{8}\leftrightarrow x_{1}\to y$ | -1.634 | -1.821 | 0.299 | 0.856 | 3.676 | 0.644 |
|  |  | $x_{8}\leftrightarrow x_{2}\to y$ | -0.375 | -0.418 |  |  |  |  |
|  |  | $x_{8}\leftrightarrow x_{3}\to y$ | 0.917 | 1.022 |  |  |  |  |
|  |  | $x_{8}\leftrightarrow x_{4}\to y$ | 0.660 | 0.736 |  |  |  |  |
|  |  | $x_{8}\leftrightarrow x_{5}\to y$ | 1.773 | 1.976 |  |  |  |  |
|  |  | $x_{8}\leftrightarrow x_{6}\to y$ | 0.438 | 0.488 |  |  |  |  |
|  |  | $x_{8}\leftrightarrow x_{7}\to y$ | -1.642 | -1.829 |  |  |  |  |
|  |  | $x_{8}\leftrightarrow x_{9}\to y$ | 0.015 | 0.016 |  |  |  |  |
|  |  | $x_{8}\leftrightarrow x_{10}\to y$ | 0.008 | 0.008 |  |  |  |  |
|  |  | $x_{8}\leftrightarrow x_{11}\to y$ | 0.140 | 0.156 |  |  |  |  |
| $\begin{matrix} x_{9} \\ \text{to} \\ y \end{matrix}$ | -0.231 | $x_{9}\leftrightarrow x_{1}\to y$ | -0.204 | 0.095 | 0.363 | 0.132 | -0.261 | -0.114 |
|  |  | $x_{9}\leftrightarrow x_{2}\to y$ | -0.035 | 0.016 |  |  |  |  |
|  |  | $x_{9}\leftrightarrow x_{3}\to y$ | 0.094 | -0.044 |  |  |  |  |
|  |  | $x_{9}\leftrightarrow x_{4}\to y$ | 0.011 | -0.005 |  |  |  |  |
|  |  | $x_{9}\leftrightarrow x_{5}\to y$ | 0.392 | -0.181 |  |  |  |  |
|  |  | $x_{9}\leftrightarrow x_{6}\to y$ | 0.121 | -0.056 |  |  |  |  |
|  |  | $x_{9}\leftrightarrow x_{7}\to y$ | -0.065 | 0.030 |  |  |  |  |
|  |  | $x_{9}\leftrightarrow x_{8}\to y$ | -0.036 | 0.016 |  |  |  |  |
|  |  | $x_{9}\leftrightarrow x_{10}\to y$ | 0.059 | -0.027 |  |  |  |  |
|  |  | $x_{9}\leftrightarrow x_{11}\to y$ | 0.026 | -0.012 |  |  |  |  |
| $\begin{matrix} x_{10} \\ \text{to} \\ y \end{matrix}$ | 0.177 | $x_{10}\leftrightarrow x_{1}\to y$ | -0.378 | -0.134 | 0.042 | 0.219 | 0.288 | 0.046 |
|  |  | $x_{10}\leftrightarrow x_{2}\to y$ | -0.115 | -0.041 |  |  |  |  |
|  |  | $x_{10}\leftrightarrow x_{3}\to y$ | 0.163 | 0.058 |  |  |  |  |
|  |  | $x_{10}\leftrightarrow x_{4}\to y$ | 0.133 | 0.047 |  |  |  |  |
|  |  | $x_{10}\leftrightarrow x_{5}\to y$ | 0.422 | 0.149 |  |  |  |  |
|  |  | $x_{10}\leftrightarrow x_{6}\to y$ | 0.033 | 0.012 |  |  |  |  |
|  |  | $x_{10}\leftrightarrow x_{7}\to y$ | -0.188 | -0.066 |  |  |  |  |
|  |  | $x_{10}\leftrightarrow x_{8}\to y$ | 0.024 | 0.008 |  |  |  |  |
|  |  | $x_{10}\leftrightarrow x_{9}\to y$ | -0.076 | -0.027 |  |  |  |  |
|  |  | $x_{10}\leftrightarrow x_{11}\to y$ | 0.025 | 0.009 |  |  |  |  |
| $\begin{matrix} x_{11} \\ \text{to} \\ y \end{matrix}$ | 0.161 | $x_{11}\leftrightarrow x_{1}\to y$ | -1.911 | -0.614 | 0.825 | 0.986 | 1.241 | 0.291 |
|  |  | $x_{11}\leftrightarrow x_{2}\to y$ | -0.396 | -0.127 |  |  |  |  |
|  |  | $x_{11}\leftrightarrow x_{3}\to y$ | 1.035 | 0.332 |  |  |  |  |
|  |  | $x_{11}\leftrightarrow x_{4}\to y$ | 0.740 | 0.238 |  |  |  |  |
|  |  | $x_{11}\leftrightarrow x_{5}\to y$ | 2.168 | 0.696 |  |  |  |  |
|  |  | $x_{11}\leftrightarrow x_{6}\to y$ | 0.513 | 0.165 |  |  |  |  |
|  |  | $x_{11}\leftrightarrow x_{7}\to y$ | -1.799 | -0.578 |  |  |  |  |
|  |  | $x_{11}\leftrightarrow x_{8}\to y$ | 0.486 | 0.156 |  |  |  |  |
|  |  | $x_{11}\leftrightarrow x_{9}\to y$ | -0.038 | -0.012 |  |  |  |  |
|  |  | $x_{11}\leftrightarrow x_{10}\to y$ | 0.027 | 0.009 |  |  |  |  |

**Table S7 (b)** The decision analysis and path analysis results of the selected subcategory pathways

| **1.5 Amino Acid Metabolism** | | | | | | | | |
| --- | --- | --- | --- | --- | --- | --- | --- | --- |
| $\begin{matrix} x_{j} \\ \text{to} \\ y \end{matrix}$ | $b_{j}^{*}$ | $x_{j}\leftrightarrow x_{k}\to y$ | $r_{jk}b_{k}^{*}$ | $2b_{j}^{*}r_{jk}b_{k}^{*}$ | $\sum_{k\neq j} r_{jk}b_{k}^{*}$ | $r_{jy}$ | $\sum_{j\neq k} r_{kj}b_{j}^{*}$ | $R_{(j)}$ |
| $\begin{matrix} x_{1} \\ \text{to} \\ y \end{matrix}$ | 0.028 | $x_{1}\leftrightarrow x_{2}\to y$ | 0.095 | 0.005 | 0.597 | 0.625 | 0.122 | 0.034 |
|  |  | $x_{1}\leftrightarrow x_{3}\to y$ | 0.206 | 0.011 |  |  |  |  |
|  |  | $x_{1}\leftrightarrow x_{4}\to y$ | -0.184 | -0.010 |  |  |  |  |
|  |  | $x_{1}\leftrightarrow x_{5}\to y$ | -0.053 | -0.003 |  |  |  |  |
|  |  | $x_{1}\leftrightarrow x_{6}\to y$ | -0.129 | -0.007 |  |  |  |  |
|  |  | $x_{1}\leftrightarrow x_{7}\to y$ | -0.077 | -0.004 |  |  |  |  |
|  |  | $x_{1}\leftrightarrow x_{8}\to y$ | 0.084 | 0.005 |  |  |  |  |
|  |  | $x_{1}\leftrightarrow x_{9}\to y$ | -0.005 | 0.000 |  |  |  |  |
|  |  | $x_{1}\leftrightarrow x_{10}\to y$ | 0.539 | 0.030 |  |  |  |  |
|  |  | $x_{1}\leftrightarrow x_{11}\to y$ | 0.121 | 0.007 |  |  |  |  |
| $\begin{matrix} x_{2} \\ \text{to} \\ y \end{matrix}$ | 0.170 | $x_{2}\leftrightarrow x_{1}\to y$ | 0.016 | 0.005 | 0.696 | 0.866 | 1.107 | 0.266 |
|  |  | $x_{2}\leftrightarrow x_{3}\to y$ | 0.303 | 0.103 |  |  |  |  |
|  |  | $x_{2}\leftrightarrow x_{4}\to y$ | -0.177 | -0.060 |  |  |  |  |
|  |  | $x_{2}\leftrightarrow x_{5}\to y$ | -0.116 | -0.039 |  |  |  |  |
|  |  | $x_{2}\leftrightarrow x_{6}\to y$ | -0.188 | -0.064 |  |  |  |  |
|  |  | $x_{2}\leftrightarrow x_{7}\to y$ | 0.015 | 0.005 |  |  |  |  |
|  |  | $x_{2}\leftrightarrow x_{8}\to y$ | 0.167 | 0.057 |  |  |  |  |
|  |  | $x_{2}\leftrightarrow x_{9}\to y$ | -0.017 | -0.006 |  |  |  |  |
|  |  | $x_{2}\leftrightarrow x_{10}\to y$ | 0.564 | 0.192 |  |  |  |  |
|  |  | $x_{2}\leftrightarrow x_{11}\to y$ | 0.132 | 0.045 |  |  |  |  |
| $\begin{matrix} x_{3} \\ \text{to} \\ y \end{matrix}$ | 0.416 | $x_{3}\leftrightarrow x_{1}\to y$ | 0.014 | 0.011 | 0.411 | 0.826 | 2.480 | 0.514 |
|  |  | $x_{3}\leftrightarrow x_{2}\to y$ | 0.124 | 0.103 |  |  |  |  |
|  |  | $x_{3}\leftrightarrow x_{4}\to y$ | -0.228 | -0.189 |  |  |  |  |
|  |  | $x_{3}\leftrightarrow x_{5}\to y$ | -0.103 | -0.085 |  |  |  |  |
|  |  | $x_{3}\leftrightarrow x_{6}\to y$ | -0.149 | -0.124 |  |  |  |  |
|  |  | $x_{3}\leftrightarrow x_{7}\to y$ | 0.018 | 0.015 |  |  |  |  |
|  |  | $x_{3}\leftrightarrow x_{8}\to y$ | 0.135 | 0.113 |  |  |  |  |
|  |  | $x_{3}\leftrightarrow x_{9}\to y$ | -0.014 | -0.012 |  |  |  |  |
|  |  | $x_{3}\leftrightarrow x_{10}\to y$ | 0.495 | 0.412 |  |  |  |  |
|  |  | $x_{3}\leftrightarrow x_{11}\to y$ | 0.119 | 0.099 |  |  |  |  |
| $\begin{matrix} x_{4} \\ \text{to} \\ y \end{matrix}$ | -0.273 | $x_{4}\leftrightarrow x_{1}\to y$ | 0.019 | -0.010 | 1.173 | 0.900 | -1.846 | -0.567 |
|  |  | $x_{4}\leftrightarrow x_{2}\to y$ | 0.110 | -0.060 |  |  |  |  |
|  |  | $x_{4}\leftrightarrow x_{3}\to y$ | 0.346 | -0.189 |  |  |  |  |
|  |  | $x_{4}\leftrightarrow x_{5}\to y$ | -0.106 | 0.058 |  |  |  |  |
|  |  | $x_{4}\leftrightarrow x_{6}\to y$ | -0.167 | 0.091 |  |  |  |  |
|  |  | $x_{4}\leftrightarrow x_{7}\to y$ | 0.043 | -0.023 |  |  |  |  |
|  |  | $x_{4}\leftrightarrow x_{8}\to y$ | 0.140 | -0.077 |  |  |  |  |
|  |  | $x_{4}\leftrightarrow x_{9}\to y$ | -0.018 | 0.010 |  |  |  |  |
|  |  | $x_{4}\leftrightarrow x_{10}\to y$ | 0.661 | -0.362 |  |  |  |  |
|  |  | $x_{4}\leftrightarrow x_{11}\to y$ | 0.145 | -0.079 |  |  |  |  |
| $\begin{matrix} x_{5} \\ \text{to} \\ y \end{matrix}$ | -0.133 | $x_{5}\leftrightarrow x_{1}\to y$ | 0.011 | -0.003 | 1.081 | 0.948 | -0.977 | -0.270 |
|  |  | $x_{5}\leftrightarrow x_{2}\to y$ | 0.148 | -0.039 |  |  |  |  |
|  |  | $x_{5}\leftrightarrow x_{3}\to y$ | 0.321 | -0.085 |  |  |  |  |
|  |  | $x_{5}\leftrightarrow x_{4}\to y$ | -0.217 | 0.058 |  |  |  |  |
|  |  | $x_{5}\leftrightarrow x_{6}\to y$ | -0.181 | 0.048 |  |  |  |  |
|  |  | $x_{5}\leftrightarrow x_{7}\to y$ | 0.111 | -0.029 |  |  |  |  |
|  |  | $x_{5}\leftrightarrow x_{8}\to y$ | 0.183 | -0.049 |  |  |  |  |
|  |  | $x_{5}\leftrightarrow x_{9}\to y$ | -0.030 | 0.008 |  |  |  |  |
|  |  | $x_{5}\leftrightarrow x_{10}\to y$ | 0.589 | -0.157 |  |  |  |  |
|  |  | $x_{5}\leftrightarrow x_{11}\to y$ | 0.145 | -0.039 |  |  |  |  |
| $\begin{matrix} x_{6} \\ \text{to} \\ y \end{matrix}$ | -0.205 | $x_{6}\leftrightarrow x_{1}\to y$ | 0.017 | -0.007 | 1.134 | 0.929 | -1.441 | -0.423 |
|  |  | $x_{6}\leftrightarrow x_{2}\to y$ | 0.156 | -0.064 |  |  |  |  |
|  |  | $x_{6}\leftrightarrow x_{3}\to y$ | 0.302 | -0.124 |  |  |  |  |
|  |  | $x_{6}\leftrightarrow x_{4}\to y$ | -0.223 | 0.091 |  |  |  |  |
|  |  | $x_{6}\leftrightarrow x_{5}\to y$ | -0.117 | 0.048 |  |  |  |  |
|  |  | $x_{6}\leftrightarrow x_{7}\to y$ | 0.008 | -0.003 |  |  |  |  |
|  |  | $x_{6}\leftrightarrow x_{8}\to y$ | 0.174 | -0.071 |  |  |  |  |
|  |  | $x_{6}\leftrightarrow x_{9}\to y$ | -0.019 | 0.008 |  |  |  |  |
|  |  | $x_{6}\leftrightarrow x_{10}\to y$ | 0.691 | -0.283 |  |  |  |  |
|  |  | $x_{6}\leftrightarrow x_{11}\to y$ | 0.144 | -0.059 |  |  |  |  |
| $\begin{matrix} x_{7} \\ \text{to} \\ y \end{matrix}$ | 0.244 | $x_{7}\leftrightarrow x_{1}\to y$ | -0.009 | -0.004 | 0.025 | 0.269 | 0.435 | 0.072 |
|  |  | $x_{7}\leftrightarrow x_{2}\to y$ | 0.010 | 0.005 |  |  |  |  |
|  |  | $x_{7}\leftrightarrow x_{3}\to y$ | 0.030 | 0.015 |  |  |  |  |
|  |  | $x_{7}\leftrightarrow x_{4}\to y$ | -0.048 | -0.023 |  |  |  |  |
|  |  | $x_{7}\leftrightarrow x_{5}\to y$ | -0.060 | -0.029 |  |  |  |  |
|  |  | $x_{7}\leftrightarrow x_{6}\to y$ | -0.007 | -0.003 |  |  |  |  |
|  |  | $x_{7}\leftrightarrow x_{8}\to y$ | 0.066 | 0.032 |  |  |  |  |
|  |  | $x_{7}\leftrightarrow x_{9}\to y$ | -0.032 | -0.015 |  |  |  |  |
|  |  | $x_{7}\leftrightarrow x_{10}\to y$ | 0.026 | 0.013 |  |  |  |  |
|  |  | $x_{7}\leftrightarrow x_{11}\to y$ | 0.049 | 0.024 |  |  |  |  |
| $\begin{matrix} x_{8} \\ \text{to} \\ y \end{matrix}$ | 0.198 | $x_{8}\leftrightarrow x_{1}\to y$ | 0.012 | 0.005 | 0.734 | 0.931 | 1.434 | 0.329 |
|  |  | $x_{8}\leftrightarrow x_{2}\to y$ | 0.143 | 0.057 |  |  |  |  |
|  |  | $x_{8}\leftrightarrow x_{3}\to y$ | 0.285 | 0.113 |  |  |  |  |
|  |  | $x_{8}\leftrightarrow x_{4}\to y$ | -0.194 | -0.077 |  |  |  |  |
|  |  | $x_{8}\leftrightarrow x_{5}\to y$ | -0.123 | -0.049 |  |  |  |  |
|  |  | $x_{8}\leftrightarrow x_{6}\to y$ | -0.180 | -0.071 |  |  |  |  |
|  |  | $x_{8}\leftrightarrow x_{7}\to y$ | 0.081 | 0.032 |  |  |  |  |
|  |  | $x_{8}\leftrightarrow x_{9}\to y$ | -0.036 | -0.014 |  |  |  |  |
|  |  | $x_{8}\leftrightarrow x_{10}\to y$ | 0.590 | 0.234 |  |  |  |  |
|  |  | $x_{8}\leftrightarrow x_{11}\to y$ | 0.156 | 0.062 |  |  |  |  |
| $\begin{matrix} x_{9} \\ \text{to} \\ y \end{matrix}$ | -0.048 | $x_{9}\leftrightarrow x_{1}\to y$ | 0.003 | 0.000 | 0.655 | 0.608 | -0.224 | -0.060 |
|  |  | $x_{9}\leftrightarrow x_{2}\to y$ | 0.062 | -0.006 |  |  |  |  |
|  |  | $x_{9}\leftrightarrow x_{3}\to y$ | 0.127 | -0.012 |  |  |  |  |
|  |  | $x_{9}\leftrightarrow x_{4}\to y$ | -0.104 | 0.010 |  |  |  |  |
|  |  | $x_{9}\leftrightarrow x_{5}\to y$ | -0.083 | 0.008 |  |  |  |  |
|  |  | $x_{9}\leftrightarrow x_{6}\to y$ | -0.080 | 0.008 |  |  |  |  |
|  |  | $x_{9}\leftrightarrow x_{7}\to y$ | 0.162 | -0.015 |  |  |  |  |
|  |  | $x_{9}\leftrightarrow x_{8}\to y$ | 0.151 | -0.014 |  |  |  |  |
|  |  | $x_{9}\leftrightarrow x_{10}\to y$ | 0.292 | -0.028 |  |  |  |  |
|  |  | $x_{9}\leftrightarrow x_{11}\to y$ | 0.126 | -0.012 |  |  |  |  |
| $\begin{matrix} x_{10} \\ \text{to} \\ y \end{matrix}$ | 0.730 | $x_{10}\leftrightarrow x_{1}\to y$ | 0.021 | 0.030 | 0.185 | 0.915 | 5.070 | 0.803 |
|  |  | $x_{10}\leftrightarrow x_{2}\to y$ | 0.131 | 0.192 |  |  |  |  |
|  |  | $x_{10}\leftrightarrow x_{3}\to y$ | 0.282 | 0.412 |  |  |  |  |
|  |  | $x_{10}\leftrightarrow x_{4}\to y$ | -0.248 | -0.362 |  |  |  |  |
|  |  | $x_{10}\leftrightarrow x_{5}\to y$ | -0.107 | -0.157 |  |  |  |  |
|  |  | $x_{10}\leftrightarrow x_{6}\to y$ | -0.194 | -0.283 |  |  |  |  |
|  |  | $x_{10}\leftrightarrow x_{7}\to y$ | 0.009 | 0.013 |  |  |  |  |
|  |  | $x_{10}\leftrightarrow x_{8}\to y$ | 0.160 | 0.234 |  |  |  |  |
|  |  | $x_{10}\leftrightarrow x_{9}\to y$ | -0.019 | -0.028 |  |  |  |  |
|  |  | $x_{10}\leftrightarrow x_{11}\to y$ | 0.151 | 0.221 |  |  |  |  |
| $\begin{matrix} x_{11} \\ \text{to} \\ y \end{matrix}$ | 0.177 | $x_{11}\leftrightarrow x_{1}\to y$ | 0.019 | 0.007 | 0.756 | 0.933 | 1.288 | 0.299 |
|  |  | $x_{11}\leftrightarrow x_{2}\to y$ | 0.127 | 0.045 |  |  |  |  |
|  |  | $x_{11}\leftrightarrow x_{3}\to y$ | 0.279 | 0.099 |  |  |  |  |
|  |  | $x_{11}\leftrightarrow x_{4}\to y$ | -0.223 | -0.079 |  |  |  |  |
|  |  | $x_{11}\leftrightarrow x_{5}\to y$ | -0.109 | -0.039 |  |  |  |  |
|  |  | $x_{11}\leftrightarrow x_{6}\to y$ | -0.167 | -0.059 |  |  |  |  |
|  |  | $x_{11}\leftrightarrow x_{7}\to y$ | 0.068 | 0.024 |  |  |  |  |
|  |  | $x_{11}\leftrightarrow x_{8}\to y$ | 0.174 | 0.062 |  |  |  |  |
|  |  | $x_{11}\leftrightarrow x_{9}\to y$ | -0.034 | -0.012 |  |  |  |  |
|  |  | $x_{11}\leftrightarrow x_{10}\to y$ | 0.622 | 0.221 |  |  |  |  |

| **3.2 Signal Transduction** | | | | | | | | |
| --- | --- | --- | --- | --- | --- | --- | --- | --- |
| $\begin{matrix} x_{j} \\ \text{to} \\ y \end{matrix}$ | $b_{j}^{*}$ | $x_{j}\leftrightarrow x_{k}\to y$ | $r_{jk}b_{k}^{*}$ | $2b_{j}^{*}r_{jk}b_{k}^{*}$ | $\sum_{k\neq j} r_{jk}b_{k}^{*}$ | $r_{jy}$ | $\sum_{j\neq k} r_{kj}b_{j}^{*}$ | $R_{(j)}$ |
| $\begin{matrix} x_{1} \\ \text{to} \\ y \end{matrix}$ | 2.990 | $x_{1}\leftrightarrow x_{2}\to y$ | -2.584 | 0.200 | -1.999 | 0.991 | 2.778 | -1.578 |
|  |  | $x_{1}\leftrightarrow x_{3}\to y$ | -0.798 | -0.201 |  |  |  |  |
|  |  | $x_{1}\leftrightarrow x_{4}\to y$ | 2.855 | -0.504 |  |  |  |  |
|  |  | $x_{1}\leftrightarrow x_{5}\to y$ | 0.953 | 0.173 |  |  |  |  |
|  |  | $x_{1}\leftrightarrow x_{6}\to y$ | 0.123 | 0.145 |  |  |  |  |
|  |  | $x_{1}\leftrightarrow x_{7}\to y$ | -1.669 | -0.876 |  |  |  |  |
|  |  | $x_{1}\leftrightarrow x_{8}\to y$ | -2.404 | -0.691 |  |  |  |  |
|  |  | $x_{1}\leftrightarrow x_{9}\to y$ | 0.292 | 0.170 |  |  |  |  |
|  |  | $x_{1}\leftrightarrow x_{10}\to y$ | -2.404 | -0.460 |  |  |  |  |
|  |  | $x_{1}\leftrightarrow x_{11}\to y$ | 3.637 | 0.095 |  |  |  |  |
| $\begin{matrix} x_{2} \\ \text{to} \\ y \end{matrix}$ | -2.623 | $x_{2}\leftrightarrow x_{1}\to y$ | 2.946 | 0.200 | 3.612 | 0.990 | -5.216 | -0.359 |
|  |  | $x_{2}\leftrightarrow x_{3}\to y$ | -0.798 | -0.055 |  |  |  |  |
|  |  | $x_{2}\leftrightarrow x_{4}\to y$ | 2.889 | -0.140 |  |  |  |  |
|  |  | $x_{2}\leftrightarrow x_{5}\to y$ | 0.950 | 0.047 |  |  |  |  |
|  |  | $x_{2}\leftrightarrow x_{6}\to y$ | 0.121 | 0.039 |  |  |  |  |
|  |  | $x_{2}\leftrightarrow x_{7}\to y$ | -1.658 | -0.239 |  |  |  |  |
|  |  | $x_{2}\leftrightarrow x_{8}\to y$ | -2.384 | -0.188 |  |  |  |  |
|  |  | $x_{2}\leftrightarrow x_{9}\to y$ | 0.294 | 0.047 |  |  |  |  |
|  |  | $x_{2}\leftrightarrow x_{10}\to y$ | -2.368 | -0.124 |  |  |  |  |
|  |  | $x_{2}\leftrightarrow x_{11}\to y$ | 3.621 | 0.026 |  |  |  |  |
| $\begin{matrix} x_{3} \\ \text{to} \\ y \end{matrix}$ | -0.835 | $x_{3}\leftrightarrow x_{1}\to y$ | 2.859 | -0.201 | 1.812 | 0.977 | -1.285 | 0.307 |
|  |  | $x_{3}\leftrightarrow x_{2}\to y$ | -2.507 | -0.055 |  |  |  |  |
|  |  | $x_{3}\leftrightarrow x_{4}\to y$ | 2.770 | 0.138 |  |  |  |  |
|  |  | $x_{3}\leftrightarrow x_{5}\to y$ | 0.940 | -0.048 |  |  |  |  |
|  |  | $x_{3}\leftrightarrow x_{6}\to y$ | 0.110 | -0.037 |  |  |  |  |
|  |  | $x_{3}\leftrightarrow x_{7}\to y$ | -1.641 | 0.244 |  |  |  |  |
|  |  | $x_{3}\leftrightarrow x_{8}\to y$ | -2.367 | 0.193 |  |  |  |  |
|  |  | $x_{3}\leftrightarrow x_{9}\to y$ | 0.313 | -0.052 |  |  |  |  |
|  |  | $x_{3}\leftrightarrow x_{10}\to y$ | -2.237 | 0.121 |  |  |  |  |
|  |  | $x_{3}\leftrightarrow x_{11}\to y$ | 3.573 | -0.026 |  |  |  |  |
| $\begin{matrix} x_{4} \\ \text{to} \\ y \end{matrix}$ | 2.906 | $x_{4}\leftrightarrow x_{1}\to y$ | 2.938 | -0.504 | -1.914 | 0.992 | 2.794 | 0.658 |
|  |  | $x_{4}\leftrightarrow x_{2}\to y$ | -2.608 | -0.140 |  |  |  |  |
|  |  | $x_{4}\leftrightarrow x_{3}\to y$ | -0.796 | 0.138 |  |  |  |  |
|  |  | $x_{4}\leftrightarrow x_{5}\to y$ | 0.952 | -0.120 |  |  |  |  |
|  |  | $x_{4}\leftrightarrow x_{6}\to y$ | 0.123 | -0.101 |  |  |  |  |
|  |  | $x_{4}\leftrightarrow x_{7}\to y$ | -1.675 | 0.608 |  |  |  |  |
|  |  | $x_{4}\leftrightarrow x_{8}\to y$ | -2.405 | 0.478 |  |  |  |  |
|  |  | $x_{4}\leftrightarrow x_{9}\to y$ | 0.302 | -0.121 |  |  |  |  |
|  |  | $x_{4}\leftrightarrow x_{10}\to y$ | -2.312 | 0.306 |  |  |  |  |
|  |  | $x_{4}\leftrightarrow x_{11}\to y$ | 3.566 | -0.064 |  |  |  |  |
| $\begin{matrix} x_{5} \\ \text{to} \\ y \end{matrix}$ | 0.966 | $x_{5}\leftrightarrow x_{1}\to y$ | 2.951 | 0.173 | 0.028 | 0.993 | -0.929 | -0.307 |
|  |  | $x_{5}\leftrightarrow x_{2}\to y$ | -2.580 | 0.047 |  |  |  |  |
|  |  | $x_{5}\leftrightarrow x_{3}\to y$ | -0.813 | -0.048 |  |  |  |  |
|  |  | $x_{5}\leftrightarrow x_{4}\to y$ | 2.866 | -0.120 |  |  |  |  |
|  |  | $x_{5}\leftrightarrow x_{6}\to y$ | 0.120 | 0.034 |  |  |  |  |
|  |  | $x_{5}\leftrightarrow x_{7}\to y$ | -1.642 | -0.204 |  |  |  |  |
|  |  | $x_{5}\leftrightarrow x_{8}\to y$ | -2.440 | -0.166 |  |  |  |  |
|  |  | $x_{5}\leftrightarrow x_{9}\to y$ | 0.298 | 0.041 |  |  |  |  |
|  |  | $x_{5}\leftrightarrow x_{10}\to y$ | -2.364 | -0.107 |  |  |  |  |
|  |  | $x_{5}\leftrightarrow x_{11}\to y$ | 3.633 | 0.022 |  |  |  |  |
| $\begin{matrix} x_{6} \\ \text{to} \\ y \end{matrix}$ | 0.129 | $x_{6}\leftrightarrow x_{1}\to y$ | 2.831 | 0.145 | 0.810 | 0.940 | -1.213 | -0.252 |
|  |  | $x_{6}\leftrightarrow x_{2}\to y$ | -2.452 | 0.039 |  |  |  |  |
|  |  | $x_{6}\leftrightarrow x_{3}\to y$ | -0.711 | -0.037 |  |  |  |  |
|  |  | $x_{6}\leftrightarrow x_{4}\to y$ | 2.771 | -0.101 |  |  |  |  |
|  |  | $x_{6}\leftrightarrow x_{5}\to y$ | 0.894 | 0.034 |  |  |  |  |
|  |  | $x_{6}\leftrightarrow x_{7}\to y$ | -1.614 | -0.175 |  |  |  |  |
|  |  | $x_{6}\leftrightarrow x_{8}\to y$ | -2.313 | -0.137 |  |  |  |  |
|  |  | $x_{6}\leftrightarrow x_{9}\to y$ | 0.280 | 0.034 |  |  |  |  |
|  |  | $x_{6}\leftrightarrow x_{10}\to y$ | -2.199 | -0.087 |  |  |  |  |
|  |  | $x_{6}\leftrightarrow x_{11}\to y$ | 3.323 | 0.018 |  |  |  |  |
| $\begin{matrix} x_{7} \\ \text{to} \\ y \end{matrix}$ | -1.720 | $x_{7}\leftrightarrow x_{1}\to y$ | 2.901 | -0.876 | 2.700 | 0.980 | -1.627 | 0.903 |
|  |  | $x_{7}\leftrightarrow x_{2}\to y$ | -2.528 | -0.239 |  |  |  |  |
|  |  | $x_{7}\leftrightarrow x_{3}\to y$ | -0.797 | 0.244 |  |  |  |  |
|  |  | $x_{7}\leftrightarrow x_{4}\to y$ | 2.829 | 0.608 |  |  |  |  |
|  |  | $x_{7}\leftrightarrow x_{5}\to y$ | 0.922 | -0.204 |  |  |  |  |
|  |  | $x_{7}\leftrightarrow x_{6}\to y$ | 0.121 | -0.175 |  |  |  |  |
|  |  | $x_{7}\leftrightarrow x_{8}\to y$ | -2.339 | 0.819 |  |  |  |  |
|  |  | $x_{7}\leftrightarrow x_{9}\to y$ | 0.316 | -0.224 |  |  |  |  |
|  |  | $x_{7}\leftrightarrow x_{10}\to y$ | -2.185 | 0.509 |  |  |  |  |
|  |  | $x_{7}\leftrightarrow x_{11}\to y$ | 3.459 | -0.110 |  |  |  |  |
| $\begin{matrix} x_{8} \\ \text{to} \\ y \end{matrix}$ | -2.457 | $x_{8}\leftrightarrow x_{1}\to y$ | 2.925 | -0.691 | 3.446 | 0.989 | -2.354 | 0.811 |
|  |  | $x_{8}\leftrightarrow x_{2}\to y$ | -2.545 | -0.188 |  |  |  |  |
|  |  | $x_{8}\leftrightarrow x_{3}\to y$ | -0.804 | 0.193 |  |  |  |  |
|  |  | $x_{8}\leftrightarrow x_{4}\to y$ | 2.844 | 0.478 |  |  |  |  |
|  |  | $x_{8}\leftrightarrow x_{5}\to y$ | 0.959 | -0.166 |  |  |  |  |
|  |  | $x_{8}\leftrightarrow x_{6}\to y$ | 0.122 | -0.137 |  |  |  |  |
|  |  | $x_{8}\leftrightarrow x_{7}\to y$ | -1.637 | 0.819 |  |  |  |  |
|  |  | $x_{8}\leftrightarrow x_{9}\to y$ | 0.302 | -0.167 |  |  |  |  |
|  |  | $x_{8}\leftrightarrow x_{10}\to y$ | -2.320 | 0.423 |  |  |  |  |
|  |  | $x_{8}\leftrightarrow x_{11}\to y$ | 3.600 | -0.090 |  |  |  |  |
| $\begin{matrix} x_{9} \\ \text{to} \\ y \end{matrix}$ | 0.333 | $x_{9}\leftrightarrow x_{1}\to y$ | 2.623 | 0.170 | 0.591 | 0.924 | -1.345 | -0.319 |
|  |  | $x_{9}\leftrightarrow x_{2}\to y$ | -2.312 | 0.047 |  |  |  |  |
|  |  | $x_{9}\leftrightarrow x_{3}\to y$ | -0.784 | -0.052 |  |  |  |  |
|  |  | $x_{9}\leftrightarrow x_{4}\to y$ | 2.634 | -0.121 |  |  |  |  |
|  |  | $x_{9}\leftrightarrow x_{5}\to y$ | 0.865 | 0.041 |  |  |  |  |
|  |  | $x_{9}\leftrightarrow x_{6}\to y$ | 0.109 | 0.034 |  |  |  |  |
|  |  | $x_{9}\leftrightarrow x_{7}\to y$ | -1.632 | -0.224 |  |  |  |  |
|  |  | $x_{9}\leftrightarrow x_{8}\to y$ | -2.229 | -0.167 |  |  |  |  |
|  |  | $x_{9}\leftrightarrow x_{10}\to y$ | -1.868 | -0.093 |  |  |  |  |
|  |  | $x_{9}\leftrightarrow x_{11}\to y$ | 3.185 | 0.022 |  |  |  |  |
| $\begin{matrix} x_{10} \\ \text{to} \\ y \end{matrix}$ | -2.503 | $x_{10}\leftrightarrow x_{1}\to y$ | 2.871 | -0.460 | 3.440 | 0.936 | -2.270 | 0.582 |
|  |  | $x_{10}\leftrightarrow x_{2}\to y$ | -2.482 | -0.124 |  |  |  |  |
|  |  | $x_{10}\leftrightarrow x_{3}\to y$ | -0.746 | 0.121 |  |  |  |  |
|  |  | $x_{10}\leftrightarrow x_{4}\to y$ | 2.684 | 0.306 |  |  |  |  |
|  |  | $x_{10}\leftrightarrow x_{5}\to y$ | 0.912 | -0.107 |  |  |  |  |
|  |  | $x_{10}\leftrightarrow x_{6}\to y$ | 0.114 | -0.087 |  |  |  |  |
|  |  | $x_{10}\leftrightarrow x_{7}\to y$ | -1.502 | 0.509 |  |  |  |  |
|  |  | $x_{10}\leftrightarrow x_{8}\to y$ | -2.278 | 0.423 |  |  |  |  |
|  |  | $x_{10}\leftrightarrow x_{9}\to y$ | 0.248 | -0.093 |  |  |  |  |
|  |  | $x_{10}\leftrightarrow x_{11}\to y$ | 3.617 | -0.061 |  |  |  |  |
| $\begin{matrix} x_{11} \\ \text{to} \\ y \end{matrix}$ | 3.706 | $x_{11}\leftrightarrow x_{1}\to y$ | 2.934 | 0.095 | -2.275 | 0.981 | 3.521 | -0.162 |
|  |  | $x_{11}\leftrightarrow x_{2}\to y$ | -2.563 | 0.026 |  |  |  |  |
|  |  | $x_{11}\leftrightarrow x_{3}\to y$ | -0.805 | -0.026 |  |  |  |  |
|  |  | $x_{11}\leftrightarrow x_{4}\to y$ | 2.796 | -0.064 |  |  |  |  |
|  |  | $x_{11}\leftrightarrow x_{5}\to y$ | 0.947 | 0.022 |  |  |  |  |
|  |  | $x_{11}\leftrightarrow x_{6}\to y$ | 0.116 | 0.018 |  |  |  |  |
|  |  | $x_{11}\leftrightarrow x_{7}\to y$ | -1.605 | -0.110 |  |  |  |  |
|  |  | $x_{11}\leftrightarrow x_{8}\to y$ | -2.388 | -0.090 |  |  |  |  |
|  |  | $x_{11}\leftrightarrow x_{9}\to y$ | 0.286 | 0.022 |  |  |  |  |
|  |  | $x_{11}\leftrightarrow x_{10}\to y$ | -2.443 | -0.061 |  |  |  |  |

| **1.7 Glycan Biosynthesis and Metabolism** | | | | | | | | |
| --- | --- | --- | --- | --- | --- | --- | --- | --- |
| $\begin{matrix} x_{j} \\ \text{to} \\ y \end{matrix}$ | $b_{j}^{*}$ | $x_{j}\leftrightarrow x_{k}\to y$ | $r_{jk}b_{k}^{*}$ | $2b_{j}^{*}r_{jk}b_{k}^{*}$ | $\sum_{k\neq j} r_{jk}b_{k}^{*}$ | $r_{jy}$ | $\sum_{j\neq k} r_{kj}b_{j}^{*}$ | $R_{(j)}$ |
| $\begin{matrix} x_{1} \\ \text{to} \\ y \end{matrix}$ | -0.508 | $x_{1}\leftrightarrow x_{2}\to y$ | -0.398 | 0.071 | 1.391 | 0.883 | -4.221 | -1.460 |
|  |  | $x_{1}\leftrightarrow x_{3}\to y$ | 0.704 | -0.075 |  |  |  |  |
|  |  | $x_{1}\leftrightarrow x_{4}\to y$ | 0.042 | 0.197 |  |  |  |  |
|  |  | $x_{1}\leftrightarrow x_{5}\to y$ | 0.814 | 0.989 |  |  |  |  |
|  |  | $x_{1}\leftrightarrow x_{6}\to y$ | 0.011 | -0.024 |  |  |  |  |
|  |  | $x_{1}\leftrightarrow x_{7}\to y$ | 0.684 | -2.013 |  |  |  |  |
|  |  | $x_{1}\leftrightarrow x_{8}\to y$ | 0.626 | -0.205 |  |  |  |  |
|  |  | $x_{1}\leftrightarrow x_{9}\to y$ | 0.375 | -0.100 |  |  |  |  |
|  |  | $x_{1}\leftrightarrow x_{10}\to y$ | -0.452 | -0.329 |  |  |  |  |
|  |  | $x_{1}\leftrightarrow x_{11}\to y$ | -0.640 | -0.219 |  |  |  |  |
|  |  | $x_{1}\leftrightarrow x_{12}\to y$ | -0.377 | -0.127 |  |  |  |  |
| $\begin{matrix} x_{2} \\ \text{to} \\ y \end{matrix}$ | -0.517 | $x_{2}\leftrightarrow x_{1}\to y$ | -0.391 | 0.071 | 1.415 | 0.898 | -4.399 | -0.142 |
|  |  | $x_{2}\leftrightarrow x_{3}\to y$ | 0.633 | -0.008 |  |  |  |  |
|  |  | $x_{2}\leftrightarrow x_{4}\to y$ | 0.044 | 0.026 |  |  |  |  |
|  |  | $x_{2}\leftrightarrow x_{5}\to y$ | 0.860 | 0.129 |  |  |  |  |
|  |  | $x_{2}\leftrightarrow x_{6}\to y$ | 0.014 | -0.004 |  |  |  |  |
|  |  | $x_{2}\leftrightarrow x_{7}\to y$ | 0.674 | -0.245 |  |  |  |  |
|  |  | $x_{2}\leftrightarrow x_{8}\to y$ | 0.575 | -0.023 |  |  |  |  |
|  |  | $x_{2}\leftrightarrow x_{9}\to y$ | 0.437 | -0.014 |  |  |  |  |
|  |  | $x_{2}\leftrightarrow x_{10}\to y$ | -0.384 | -0.034 |  |  |  |  |
|  |  | $x_{2}\leftrightarrow x_{11}\to y$ | -0.632 | -0.027 |  |  |  |  |
|  |  | $x_{2}\leftrightarrow x_{12}\to y$ | -0.416 | -0.017 |  |  |  |  |
| $\begin{matrix} x_{3} \\ \text{to} \\ y \end{matrix}$ | 0.925 | $x_{3}\leftrightarrow x_{1}\to y$ | -0.387 | -0.075 | -0.054 | 0.870 | 8.042 | 0.134 |
|  |  | $x_{3}\leftrightarrow x_{2}\to y$ | -0.354 | -0.008 |  |  |  |  |
|  |  | $x_{3}\leftrightarrow x_{4}\to y$ | 0.047 | -0.029 |  |  |  |  |
|  |  | $x_{3}\leftrightarrow x_{5}\to y$ | 0.944 | -0.151 |  |  |  |  |
|  |  | $x_{3}\leftrightarrow x_{6}\to y$ | 0.013 | 0.004 |  |  |  |  |
|  |  | $x_{3}\leftrightarrow x_{7}\to y$ | 0.606 | 0.235 |  |  |  |  |
|  |  | $x_{3}\leftrightarrow x_{8}\to y$ | 0.506 | 0.022 |  |  |  |  |
|  |  | $x_{3}\leftrightarrow x_{9}\to y$ | 0.374 | 0.013 |  |  |  |  |
|  |  | $x_{3}\leftrightarrow x_{10}\to y$ | -0.718 | 0.069 |  |  |  |  |
|  |  | $x_{3}\leftrightarrow x_{11}\to y$ | -0.654 | 0.030 |  |  |  |  |
|  |  | $x_{3}\leftrightarrow x_{12}\to y$ | -0.432 | 0.019 |  |  |  |  |
| $\begin{matrix} x_{4} \\ \text{to} \\ y \end{matrix}$ | 0.052 | $x_{4}\leftrightarrow x_{1}\to y$ | -0.413 | 0.197 | 0.887 | 0.939 | -1.490 | -0.411 |
|  |  | $x_{4}\leftrightarrow x_{2}\to y$ | -0.439 | 0.026 |  |  |  |  |
|  |  | $x_{4}\leftrightarrow x_{3}\to y$ | 0.836 | -0.029 |  |  |  |  |
|  |  | $x_{4}\leftrightarrow x_{5}\to y$ | 1.012 | 0.397 |  |  |  |  |
|  |  | $x_{4}\leftrightarrow x_{6}\to y$ | 0.015 | -0.011 |  |  |  |  |
|  |  | $x_{4}\leftrightarrow x_{7}\to y$ | 0.696 | -0.661 |  |  |  |  |
|  |  | $x_{4}\leftrightarrow x_{8}\to y$ | 0.534 | -0.056 |  |  |  |  |
|  |  | $x_{4}\leftrightarrow x_{9}\to y$ | 0.436 | -0.037 |  |  |  |  |
|  |  | $x_{4}\leftrightarrow x_{10}\to y$ | -0.626 | -0.147 |  |  |  |  |
|  |  | $x_{4}\leftrightarrow x_{11}\to y$ | -0.654 | -0.072 |  |  |  |  |
|  |  | $x_{4}\leftrightarrow x_{12}\to y$ | -0.511 | -0.056 |  |  |  |  |
| $\begin{matrix} x_{5} \\ \text{to} \\ y \end{matrix}$ | 1.519 | $x_{5}\leftrightarrow x_{1}\to y$ | -0.357 | 0.989 | -0.234 | 0.925 | 10.697 | -3.439 |
|  |  | $x_{5}\leftrightarrow x_{2}\to y$ | -0.383 | 0.129 |  |  |  |  |
|  |  | $x_{5}\leftrightarrow x_{3}\to y$ | 0.753 | -0.151 |  |  |  |  |
|  |  | $x_{5}\leftrightarrow x_{4}\to y$ | 0.045 | 0.397 |  |  |  |  |
|  |  | $x_{5}\leftrightarrow x_{6}\to y$ | 0.016 | -0.067 |  |  |  |  |
|  |  | $x_{5}\leftrightarrow x_{7}\to y$ | 0.683 | -3.760 |  |  |  |  |
|  |  | $x_{5}\leftrightarrow x_{8}\to y$ | 0.540 | -0.331 |  |  |  |  |
|  |  | $x_{5}\leftrightarrow x_{9}\to y$ | 0.395 | -0.196 |  |  |  |  |
|  |  | $x_{5}\leftrightarrow x_{10}\to y$ | -0.739 | -1.007 |  |  |  |  |
|  |  | $x_{5}\leftrightarrow x_{11}\to y$ | -0.696 | -0.446 |  |  |  |  |
|  |  | $x_{5}\leftrightarrow x_{12}\to y$ | -0.492 | -0.310 |  |  |  |  |
| $\begin{matrix} x_{6} \\ \text{to} \\ y \end{matrix}$ | 0.017 | $x_{6}\leftrightarrow x_{1}\to y$ | -0.321 | -0.024 | 0.886 | 0.903 | 0.155 | 0.055 |
|  |  | $x_{6}\leftrightarrow x_{2}\to y$ | -0.417 | -0.004 |  |  |  |  |
|  |  | $x_{6}\leftrightarrow x_{3}\to y$ | 0.702 | 0.004 |  |  |  |  |
|  |  | $x_{6}\leftrightarrow x_{4}\to y$ | 0.046 | -0.011 |  |  |  |  |
|  |  | $x_{6}\leftrightarrow x_{5}\to y$ | 1.095 | -0.067 |  |  |  |  |
|  |  | $x_{6}\leftrightarrow x_{7}\to y$ | 0.653 | 0.098 |  |  |  |  |
|  |  | $x_{6}\leftrightarrow x_{8}\to y$ | 0.527 | 0.009 |  |  |  |  |
|  |  | $x_{6}\leftrightarrow x_{9}\to y$ | 0.407 | 0.006 |  |  |  |  |
|  |  | $x_{6}\leftrightarrow x_{10}\to y$ | -0.702 | 0.026 |  |  |  |  |
|  |  | $x_{6}\leftrightarrow x_{11}\to y$ | -0.658 | 0.011 |  |  |  |  |
|  |  | $x_{6}\leftrightarrow x_{12}\to y$ | -0.446 | 0.008 |  |  |  |  |
| $\begin{matrix} x_{7} \\ \text{to} \\ y \end{matrix}$ | 0.753 | $x_{7}\leftrightarrow x_{1}\to y$ | -0.461 | -2.013 | 0.231 | 0.984 | 7.219 | 0.291 |
|  |  | $x_{7}\leftrightarrow x_{2}\to y$ | -0.463 | -0.245 |  |  |  |  |
|  |  | $x_{7}\leftrightarrow x_{3}\to y$ | 0.744 | 0.235 |  |  |  |  |
|  |  | $x_{7}\leftrightarrow x_{4}\to y$ | 0.048 | -0.661 |  |  |  |  |
|  |  | $x_{7}\leftrightarrow x_{5}\to y$ | 1.051 | -3.760 |  |  |  |  |
|  |  | $x_{7}\leftrightarrow x_{6}\to y$ | 0.015 | 0.098 |  |  |  |  |
|  |  | $x_{7}\leftrightarrow x_{8}\to y$ | 0.629 | 0.607 |  |  |  |  |
|  |  | $x_{7}\leftrightarrow x_{9}\to y$ | 0.436 | 0.342 |  |  |  |  |
|  |  | $x_{7}\leftrightarrow x_{10}\to y$ | -0.568 | 1.218 |  |  |  |  |
|  |  | $x_{7}\leftrightarrow x_{11}\to y$ | -0.717 | 0.725 |  |  |  |  |
|  |  | $x_{7}\leftrightarrow x_{12}\to y$ | -0.483 | 0.480 |  |  |  |  |
| $\begin{matrix} x_{8} \\ \text{to} \\ y \end{matrix}$ | 0.681 | $x_{8}\leftrightarrow x_{1}\to y$ | -0.467 | -0.205 | 0.255 | 0.936 | 5.955 | 0.307 |
|  |  | $x_{8}\leftrightarrow x_{2}\to y$ | -0.437 | -0.023 |  |  |  |  |
|  |  | $x_{8}\leftrightarrow x_{3}\to y$ | 0.688 | 0.022 |  |  |  |  |
|  |  | $x_{8}\leftrightarrow x_{4}\to y$ | 0.041 | -0.056 |  |  |  |  |
|  |  | $x_{8}\leftrightarrow x_{5}\to y$ | 0.920 | -0.331 |  |  |  |  |
|  |  | $x_{8}\leftrightarrow x_{6}\to y$ | 0.013 | 0.009 |  |  |  |  |
|  |  | $x_{8}\leftrightarrow x_{7}\to y$ | 0.696 | 0.607 |  |  |  |  |
|  |  | $x_{8}\leftrightarrow x_{9}\to y$ | 0.390 | 0.031 |  |  |  |  |
|  |  | $x_{8}\leftrightarrow x_{10}\to y$ | -0.535 | 0.115 |  |  |  |  |
|  |  | $x_{8}\leftrightarrow x_{11}\to y$ | -0.723 | 0.074 |  |  |  |  |
|  |  | $x_{8}\leftrightarrow x_{12}\to y$ | -0.331 | 0.033 |  |  |  |  |
| $\begin{matrix} x_{9} \\ \text{to} \\ y \end{matrix}$ | 0.456 | $x_{9}\leftrightarrow x_{1}\to y$ | -0.418 | -0.100 | 0.508 | 0.963 | 4.314 | 0.181 |
|  |  | $x_{9}\leftrightarrow x_{2}\to y$ | -0.496 | -0.014 |  |  |  |  |
|  |  | $x_{9}\leftrightarrow x_{3}\to y$ | 0.759 | 0.013 |  |  |  |  |
|  |  | $x_{9}\leftrightarrow x_{4}\to y$ | 0.050 | -0.037 |  |  |  |  |
|  |  | $x_{9}\leftrightarrow x_{5}\to y$ | 1.005 | -0.196 |  |  |  |  |
|  |  | $x_{9}\leftrightarrow x_{6}\to y$ | 0.015 | 0.006 |  |  |  |  |
|  |  | $x_{9}\leftrightarrow x_{7}\to y$ | 0.721 | 0.342 |  |  |  |  |
|  |  | $x_{9}\leftrightarrow x_{8}\to y$ | 0.583 | 0.031 |  |  |  |  |
|  |  | $x_{9}\leftrightarrow x_{10}\to y$ | -0.547 | 0.064 |  |  |  |  |
|  |  | $x_{9}\leftrightarrow x_{11}\to y$ | -0.669 | 0.037 |  |  |  |  |
|  |  | $x_{9}\leftrightarrow x_{12}\to y$ | -0.496 | 0.027 |  |  |  |  |
| $\begin{matrix} x_{10} \\ \text{to} \\ y \end{matrix}$ | -0.858 | $x_{10}\leftrightarrow x_{1}\to y$ | -0.268 | -0.329 | 1.602 | 0.745 | -6.428 | 0.499 |
|  |  | $x_{10}\leftrightarrow x_{2}\to y$ | -0.231 | -0.034 |  |  |  |  |
|  |  | $x_{10}\leftrightarrow x_{3}\to y$ | 0.775 | 0.069 |  |  |  |  |
|  |  | $x_{10}\leftrightarrow x_{4}\to y$ | 0.038 | -0.147 |  |  |  |  |
|  |  | $x_{10}\leftrightarrow x_{5}\to y$ | 0.999 | -1.007 |  |  |  |  |
|  |  | $x_{10}\leftrightarrow x_{6}\to y$ | 0.014 | 0.026 |  |  |  |  |
|  |  | $x_{10}\leftrightarrow x_{7}\to y$ | 0.499 | 1.218 |  |  |  |  |
|  |  | $x_{10}\leftrightarrow x_{8}\to y$ | 0.424 | 0.115 |  |  |  |  |
|  |  | $x_{10}\leftrightarrow x_{9}\to y$ | 0.290 | 0.064 |  |  |  |  |
|  |  | $x_{10}\leftrightarrow x_{11}\to y$ | -0.576 | 0.164 |  |  |  |  |
|  |  | $x_{10}\leftrightarrow x_{12}\to y$ | -0.363 | 0.101 |  |  |  |  |
| $\begin{matrix} x_{11} \\ \text{to} \\ y \end{matrix}$ | -0.784 | $x_{11}\leftrightarrow x_{1}\to y$ | -0.414 | -0.219 | 1.733 | 0.949 | -7.112 | 0.368 |
|  |  | $x_{11}\leftrightarrow x_{2}\to y$ | -0.417 | -0.027 |  |  |  |  |
|  |  | $x_{11}\leftrightarrow x_{3}\to y$ | 0.771 | 0.030 |  |  |  |  |
|  |  | $x_{11}\leftrightarrow x_{4}\to y$ | 0.043 | -0.072 |  |  |  |  |
|  |  | $x_{11}\leftrightarrow x_{5}\to y$ | 1.029 | -0.446 |  |  |  |  |
|  |  | $x_{11}\leftrightarrow x_{6}\to y$ | 0.014 | 0.011 |  |  |  |  |
|  |  | $x_{11}\leftrightarrow x_{7}\to y$ | 0.690 | 0.725 |  |  |  |  |
|  |  | $x_{11}\leftrightarrow x_{8}\to y$ | 0.628 | 0.074 |  |  |  |  |
|  |  | $x_{11}\leftrightarrow x_{9}\to y$ | 0.389 | 0.037 |  |  |  |  |
|  |  | $x_{11}\leftrightarrow x_{10}\to y$ | -0.630 | 0.164 |  |  |  |  |
|  |  | $x_{11}\leftrightarrow x_{12}\to y$ | -0.371 | 0.045 |  |  |  |  |
| $\begin{matrix} x_{12} \\ \text{to} \\ y \end{matrix}$ | -0.589 | $x_{12}\leftrightarrow x_{1}\to y$ | -0.325 | -0.127 | 1.377 | 0.788 | -4.717 | 0.229 |
|  |  | $x_{12}\leftrightarrow x_{2}\to y$ | -0.365 | -0.017 |  |  |  |  |
|  |  | $x_{12}\leftrightarrow x_{3}\to y$ | 0.678 | 0.019 |  |  |  |  |
|  |  | $x_{12}\leftrightarrow x_{4}\to y$ | 0.045 | -0.056 |  |  |  |  |
|  |  | $x_{12}\leftrightarrow x_{5}\to y$ | 0.968 | -0.310 |  |  |  |  |
|  |  | $x_{12}\leftrightarrow x_{6}\to y$ | 0.013 | 0.008 |  |  |  |  |
|  |  | $x_{12}\leftrightarrow x_{7}\to y$ | 0.618 | 0.480 |  |  |  |  |
|  |  | $x_{12}\leftrightarrow x_{8}\to y$ | 0.383 | 0.033 |  |  |  |  |
|  |  | $x_{12}\leftrightarrow x_{9}\to y$ | 0.384 | 0.027 |  |  |  |  |
|  |  | $x_{12}\leftrightarrow x_{10}\to y$ | -0.528 | 0.101 |  |  |  |  |
|  |  | $x_{12}\leftrightarrow x_{11}\to y$ | -0.494 | 0.045 |  |  |  |  |

| **1.3 Lipid Metabolism** | | | | | | | | |
| --- | --- | --- | --- | --- | --- | --- | --- | --- |
| $\begin{matrix} x_{j} \\ \text{to} \\ y \end{matrix}$ | $b_{j}^{*}$ | $x_{j}\leftrightarrow x_{k}\to y$ | $r_{jk}b_{k}^{*}$ | $2b_{j}^{*}r_{jk}b_{k}^{*}$ | $\sum_{k\neq j} r_{jk}b_{k}^{*}$ | $r_{jy}$ | $\sum_{j\neq k} r_{kj}b_{j}^{*}$ | $R_{(j)}$ |
| $\begin{matrix} x_{1} \\ \text{to} \\ y \end{matrix}$ | -5.489 | $x_{1}\leftrightarrow x_{2}\to y$ | 3.254 | 0.458 | 6.304 | 0.815 | -4.290 | 0.265 |
|  |  | $x_{1}\leftrightarrow x_{3}\to y$ | 3.870 | 0.001 |  |  |  |  |
|  |  | $x_{1}\leftrightarrow x_{4}\to y$ | 5.634 | 0.010 |  |  |  |  |
|  |  | $x_{1}\leftrightarrow x_{5}\to y$ | -1.841 | -0.063 |  |  |  |  |
|  |  | $x_{1}\leftrightarrow x_{6}\to y$ | -2.051 | -0.192 |  |  |  |  |
|  |  | $x_{1}\leftrightarrow x_{7}\to y$ | -4.829 | 0.011 |  |  |  |  |
|  |  | $x_{1}\leftrightarrow x_{8}\to y$ | -3.141 | -0.154 |  |  |  |  |
|  |  | $x_{1}\leftrightarrow x_{9}\to y$ | -0.293 | -0.021 |  |  |  |  |
|  |  | $x_{1}\leftrightarrow x_{10}\to y$ | 5.087 | 0.000 |  |  |  |  |
|  |  | $x_{1}\leftrightarrow x_{11}\to y$ | -6.826 | -0.016 |  |  |  |  |
|  |  | $x_{1}\leftrightarrow x_{12}\to y$ | 8.306 | 0.133 |  |  |  |  |
|  |  | $x_{1}\leftrightarrow x_{13}\to y$ | -0.867 | 0.063 |  |  |  |  |
| $\begin{matrix} x_{2} \\ \text{to} \\ y \end{matrix}$ | 4.112 | $x_{2}\leftrightarrow x_{1}\to y$ | -4.343 | 0.458 | -3.141 | 0.971 | 3.792 | 0.571 |
|  |  | $x_{2}\leftrightarrow x_{3}\to y$ | 5.685 | 0.017 |  |  |  |  |
|  |  | $x_{2}\leftrightarrow x_{4}\to y$ | 6.102 | 0.089 |  |  |  |  |
|  |  | $x_{2}\leftrightarrow x_{5}\to y$ | -1.777 | -0.526 |  |  |  |  |
|  |  | $x_{2}\leftrightarrow x_{6}\to y$ | -2.649 | -2.135 |  |  |  |  |
|  |  | $x_{2}\leftrightarrow x_{7}\to y$ | -6.808 | 0.139 |  |  |  |  |
|  |  | $x_{2}\leftrightarrow x_{8}\to y$ | -3.663 | -1.547 |  |  |  |  |
|  |  | $x_{2}\leftrightarrow x_{9}\to y$ | 0.286 | 0.172 |  |  |  |  |
|  |  | $x_{2}\leftrightarrow x_{10}\to y$ | 6.689 | 0.001 |  |  |  |  |
|  |  | $x_{2}\leftrightarrow x_{11}\to y$ | -7.234 | -0.142 |  |  |  |  |
|  |  | $x_{2}\leftrightarrow x_{12}\to y$ | 5.774 | 0.797 |  |  |  |  |
|  |  | $x_{2}\leftrightarrow x_{13}\to y$ | -1.203 | 0.752 |  |  |  |  |
| $\begin{matrix} x_{3} \\ \text{to} \\ y \end{matrix}$ | 7.281 | $x_{3}\leftrightarrow x_{1}\to y$ | -2.917 | 0.001 | -6.427 | 0.855 | 6.103 | 0.012 |
|  |  | $x_{3}\leftrightarrow x_{2}\to y$ | 3.211 | 0.017 |  |  |  |  |
|  |  | $x_{3}\leftrightarrow x_{4}\to y$ | 5.006 | 0.000 |  |  |  |  |
|  |  | $x_{3}\leftrightarrow x_{5}\to y$ | -2.011 | -0.003 |  |  |  |  |
|  |  | $x_{3}\leftrightarrow x_{6}\to y$ | -2.097 | -0.007 |  |  |  |  |
|  |  | $x_{3}\leftrightarrow x_{7}\to y$ | -5.494 | 0.001 |  |  |  |  |
|  |  | $x_{3}\leftrightarrow x_{8}\to y$ | -2.811 | -0.005 |  |  |  |  |
|  |  | $x_{3}\leftrightarrow x_{9}\to y$ | 0.386 | 0.001 |  |  |  |  |
|  |  | $x_{3}\leftrightarrow x_{10}\to y$ | 5.863 | 0.000 |  |  |  |  |
|  |  | $x_{3}\leftrightarrow x_{11}\to y$ | -7.886 | -0.001 |  |  |  |  |
|  |  | $x_{3}\leftrightarrow x_{12}\to y$ | 4.107 | 0.002 |  |  |  |  |
|  |  | $x_{3}\leftrightarrow x_{13}\to y$ | -1.784 | 0.005 |  |  |  |  |
| $\begin{matrix} x_{4} \\ \text{to} \\ y \end{matrix}$ | 6.617 | $x_{4}\leftrightarrow x_{1}\to y$ | -4.673 | 0.010 | -5.638 | 0.934 | 5.975 | 0.056 |
|  |  | $x_{4}\leftrightarrow x_{2}\to y$ | 3.792 | 0.089 |  |  |  |  |
|  |  | $x_{4}\leftrightarrow x_{3}\to y$ | 5.509 | 0.000 |  |  |  |  |
|  |  | $x_{4}\leftrightarrow x_{5}\to y$ | -1.781 | -0.010 |  |  |  |  |
|  |  | $x_{4}\leftrightarrow x_{6}\to y$ | -2.306 | -0.036 |  |  |  |  |
|  |  | $x_{4}\leftrightarrow x_{7}\to y$ | -6.358 | 0.003 |  |  |  |  |
|  |  | $x_{4}\leftrightarrow x_{8}\to y$ | -3.750 | -0.031 |  |  |  |  |
|  |  | $x_{4}\leftrightarrow x_{9}\to y$ | -0.082 | -0.001 |  |  |  |  |
|  |  | $x_{4}\leftrightarrow x_{10}\to y$ | 6.284 | 0.000 |  |  |  |  |
|  |  | $x_{4}\leftrightarrow x_{11}\to y$ | -7.472 | -0.003 |  |  |  |  |
|  |  | $x_{4}\leftrightarrow x_{12}\to y$ | 6.551 | 0.017 |  |  |  |  |
|  |  | $x_{4}\leftrightarrow x_{13}\to y$ | -1.397 | 0.017 |  |  |  |  |
| $\begin{matrix} x_{5} \\ \text{to} \\ y \end{matrix}$ | -2.546 | $x_{5}\leftrightarrow x_{1}\to y$ | -3.968 | -0.063 | 3.313 | 0.766 | -1.914 | -0.422 |
|  |  | $x_{5}\leftrightarrow x_{2}\to y$ | 2.870 | -0.526 |  |  |  |  |
|  |  | $x_{5}\leftrightarrow x_{3}\to y$ | 5.750 | -0.003 |  |  |  |  |
|  |  | $x_{5}\leftrightarrow x_{4}\to y$ | 4.627 | -0.010 |  |  |  |  |
|  |  | $x_{5}\leftrightarrow x_{6}\to y$ | -1.519 | 0.185 |  |  |  |  |
|  |  | $x_{5}\leftrightarrow x_{7}\to y$ | -3.824 | -0.012 |  |  |  |  |
|  |  | $x_{5}\leftrightarrow x_{8}\to y$ | -2.505 | 0.160 |  |  |  |  |
|  |  | $x_{5}\leftrightarrow x_{9}\to y$ | -0.308 | 0.028 |  |  |  |  |
|  |  | $x_{5}\leftrightarrow x_{10}\to y$ | 5.665 | 0.000 |  |  |  |  |
|  |  | $x_{5}\leftrightarrow x_{11}\to y$ | -7.754 | 0.023 |  |  |  |  |
|  |  | $x_{5}\leftrightarrow x_{12}\to y$ | 5.770 | -0.120 |  |  |  |  |
|  |  | $x_{5}\leftrightarrow x_{13}\to y$ | -1.491 | -0.141 |  |  |  |  |
| $\begin{matrix} x_{6} \\ \text{to} \\ y \end{matrix}$ | -2.837 | $x_{6}\leftrightarrow x_{1}\to y$ | -3.967 | -0.192 | 3.741 | 0.904 | -2.428 | -1.832 |
|  |  | $x_{6}\leftrightarrow x_{2}\to y$ | 3.838 | -2.135 |  |  |  |  |
|  |  | $x_{6}\leftrightarrow x_{3}\to y$ | 5.381 | -0.007 |  |  |  |  |
|  |  | $x_{6}\leftrightarrow x_{4}\to y$ | 5.377 | -0.036 |  |  |  |  |
|  |  | $x_{6}\leftrightarrow x_{5}\to y$ | -1.363 | 0.185 |  |  |  |  |
|  |  | $x_{6}\leftrightarrow x_{7}\to y$ | -6.750 | -0.063 |  |  |  |  |
|  |  | $x_{6}\leftrightarrow x_{8}\to y$ | -3.302 | 0.639 |  |  |  |  |
|  |  | $x_{6}\leftrightarrow x_{9}\to y$ | 0.636 | -0.175 |  |  |  |  |
|  |  | $x_{6}\leftrightarrow x_{10}\to y$ | 5.698 | -0.001 |  |  |  |  |
|  |  | $x_{6}\leftrightarrow x_{11}\to y$ | -6.313 | 0.057 |  |  |  |  |
|  |  | $x_{6}\leftrightarrow x_{12}\to y$ | 5.485 | -0.347 |  |  |  |  |
|  |  | $x_{6}\leftrightarrow x_{13}\to y$ | -0.980 | -0.281 |  |  |  |  |
| $\begin{matrix} x_{7} \\ \text{to} \\ y \end{matrix}$ | -7.134 | $x_{7}\leftrightarrow x_{1}\to y$ | -3.715 | 0.011 | 8.038 | 0.904 | -6.212 | 0.081 |
|  |  | $x_{7}\leftrightarrow x_{2}\to y$ | 3.924 | 0.139 |  |  |  |  |
|  |  | $x_{7}\leftrightarrow x_{3}\to y$ | 5.608 | 0.001 |  |  |  |  |
|  |  | $x_{7}\leftrightarrow x_{4}\to y$ | 5.897 | 0.003 |  |  |  |  |
|  |  | $x_{7}\leftrightarrow x_{5}\to y$ | -1.365 | -0.012 |  |  |  |  |
|  |  | $x_{7}\leftrightarrow x_{6}\to y$ | -2.685 | -0.063 |  |  |  |  |
|  |  | $x_{7}\leftrightarrow x_{8}\to y$ | -3.620 | -0.045 |  |  |  |  |
|  |  | $x_{7}\leftrightarrow x_{9}\to y$ | 0.594 | 0.010 |  |  |  |  |
|  |  | $x_{7}\leftrightarrow x_{10}\to y$ | 6.144 | 0.000 |  |  |  |  |
|  |  | $x_{7}\leftrightarrow x_{11}\to y$ | -6.271 | -0.004 |  |  |  |  |
|  |  | $x_{7}\leftrightarrow x_{12}\to y$ | 4.609 | 0.019 |  |  |  |  |
|  |  | $x_{7}\leftrightarrow x_{13}\to y$ | -1.082 | 0.020 |  |  |  |  |
| $\begin{matrix} x_{8} \\ \text{to} \\ y \end{matrix}$ | -3.823 | $x_{8}\leftrightarrow x_{1}\to y$ | -4.508 | -0.154 | 4.752 | 0.929 | -3.442 | -1.210 |
|  |  | $x_{8}\leftrightarrow x_{2}\to y$ | 3.939 | -1.547 |  |  |  |  |
|  |  | $x_{8}\leftrightarrow x_{3}\to y$ | 5.353 | -0.005 |  |  |  |  |
|  |  | $x_{8}\leftrightarrow x_{4}\to y$ | 6.490 | -0.031 |  |  |  |  |
|  |  | $x_{8}\leftrightarrow x_{5}\to y$ | -1.668 | 0.160 |  |  |  |  |
|  |  | $x_{8}\leftrightarrow x_{6}\to y$ | -2.450 | 0.639 |  |  |  |  |
|  |  | $x_{8}\leftrightarrow x_{7}\to y$ | -6.755 | -0.045 |  |  |  |  |
|  |  | $x_{8}\leftrightarrow x_{9}\to y$ | 0.153 | -0.030 |  |  |  |  |
|  |  | $x_{8}\leftrightarrow x_{10}\to y$ | 6.414 | 0.000 |  |  |  |  |
|  |  | $x_{8}\leftrightarrow x_{11}\to y$ | -6.965 | 0.044 |  |  |  |  |
|  |  | $x_{8}\leftrightarrow x_{12}\to y$ | 5.930 | -0.265 |  |  |  |  |
|  |  | $x_{8}\leftrightarrow x_{13}\to y$ | -1.180 | -0.239 |  |  |  |  |
| $\begin{matrix} x_{9} \\ \text{to} \\ y \end{matrix}$ | 1.695 | $x_{9}\leftrightarrow x_{1}\to y$ | 0.949 | -0.021 | -1.605 | 0.090 | 0.536 | -0.046 |
|  |  | $x_{9}\leftrightarrow x_{2}\to y$ | 0.693 | 0.172 |  |  |  |  |
|  |  | $x_{9}\leftrightarrow x_{3}\to y$ | 1.659 | 0.001 |  |  |  |  |
|  |  | $x_{9}\leftrightarrow x_{4}\to y$ | -0.322 | -0.001 |  |  |  |  |
|  |  | $x_{9}\leftrightarrow x_{5}\to y$ | 0.463 | 0.028 |  |  |  |  |
|  |  | $x_{9}\leftrightarrow x_{6}\to y$ | -1.064 | -0.175 |  |  |  |  |
|  |  | $x_{9}\leftrightarrow x_{7}\to y$ | -2.499 | 0.010 |  |  |  |  |
|  |  | $x_{9}\leftrightarrow x_{8}\to y$ | -0.345 | -0.030 |  |  |  |  |
|  |  | $x_{9}\leftrightarrow x_{10}\to y$ | 0.503 | 0.000 |  |  |  |  |
|  |  | $x_{9}\leftrightarrow x_{11}\to y$ | 0.284 | 0.001 |  |  |  |  |
|  |  | $x_{9}\leftrightarrow x_{12}\to y$ | -2.453 | -0.069 |  |  |  |  |
|  |  | $x_{9}\leftrightarrow x_{13}\to y$ | 0.527 | -0.067 |  |  |  |  |
| $\begin{matrix} x_{10} \\ \text{to} \\ y \end{matrix}$ | 6.962 | $x_{10}\leftrightarrow x_{1}\to y$ | -4.010 | 0.000 | -6.009 | 0.953 | 6.328 | 0.001 |
|  |  | $x_{10}\leftrightarrow x_{2}\to y$ | 3.951 | 0.001 |  |  |  |  |
|  |  | $x_{10}\leftrightarrow x_{3}\to y$ | 6.132 | 0.000 |  |  |  |  |
|  |  | $x_{10}\leftrightarrow x_{4}\to y$ | 5.973 | 0.000 |  |  |  |  |
|  |  | $x_{10}\leftrightarrow x_{5}\to y$ | -2.072 | 0.000 |  |  |  |  |
|  |  | $x_{10}\leftrightarrow x_{6}\to y$ | -2.322 | -0.001 |  |  |  |  |
|  |  | $x_{10}\leftrightarrow x_{7}\to y$ | -6.296 | 0.000 |  |  |  |  |
|  |  | $x_{10}\leftrightarrow x_{8}\to y$ | -3.523 | 0.000 |  |  |  |  |
|  |  | $x_{10}\leftrightarrow x_{9}\to y$ | 0.123 | 0.000 |  |  |  |  |
|  |  | $x_{10}\leftrightarrow x_{11}\to y$ | -7.717 | 0.000 |  |  |  |  |
|  |  | $x_{10}\leftrightarrow x_{12}\to y$ | 5.179 | 0.000 |  |  |  |  |
|  |  | $x_{10}\leftrightarrow x_{13}\to y$ | -1.427 | 0.000 |  |  |  |  |
| $\begin{matrix} x_{11} \\ \text{to} \\ y \end{matrix}$ | -8.846 | $x_{11}\leftrightarrow x_{1}\to y$ | -4.235 | -0.016 | 9.769 | 0.923 | -7.785 | -0.105 |
|  |  | $x_{11}\leftrightarrow x_{2}\to y$ | 3.363 | -0.142 |  |  |  |  |
|  |  | $x_{11}\leftrightarrow x_{3}\to y$ | 6.491 | -0.001 |  |  |  |  |
|  |  | $x_{11}\leftrightarrow x_{4}\to y$ | 5.589 | -0.003 |  |  |  |  |
|  |  | $x_{11}\leftrightarrow x_{5}\to y$ | -2.232 | 0.023 |  |  |  |  |
|  |  | $x_{11}\leftrightarrow x_{6}\to y$ | -2.025 | 0.057 |  |  |  |  |
|  |  | $x_{11}\leftrightarrow x_{7}\to y$ | -5.057 | -0.004 |  |  |  |  |
|  |  | $x_{11}\leftrightarrow x_{8}\to y$ | -3.010 | 0.044 |  |  |  |  |
|  |  | $x_{11}\leftrightarrow x_{9}\to y$ | -0.054 | 0.001 |  |  |  |  |
|  |  | $x_{11}\leftrightarrow x_{10}\to y$ | 6.074 | 0.000 |  |  |  |  |
|  |  | $x_{11}\leftrightarrow x_{12}\to y$ | 6.544 | -0.031 |  |  |  |  |
|  |  | $x_{11}\leftrightarrow x_{13}\to y$ | -1.678 | -0.037 |  |  |  |  |
| $\begin{matrix} x_{12} \\ \text{to} \\ y \end{matrix}$ | 8.659 | $x_{12}\leftrightarrow x_{1}\to y$ | -5.265 | 0.133 | -7.915 | 0.744 | 5.965 | 0.420 |
|  |  | $x_{12}\leftrightarrow x_{2}\to y$ | 2.742 | 0.797 |  |  |  |  |
|  |  | $x_{12}\leftrightarrow x_{3}\to y$ | 3.454 | 0.002 |  |  |  |  |
|  |  | $x_{12}\leftrightarrow x_{4}\to y$ | 5.006 | 0.017 |  |  |  |  |
|  |  | $x_{12}\leftrightarrow x_{5}\to y$ | -1.697 | -0.120 |  |  |  |  |
|  |  | $x_{12}\leftrightarrow x_{6}\to y$ | -1.797 | -0.347 |  |  |  |  |
|  |  | $x_{12}\leftrightarrow x_{7}\to y$ | -3.797 | 0.019 |  |  |  |  |
|  |  | $x_{12}\leftrightarrow x_{8}\to y$ | -2.618 | -0.265 |  |  |  |  |
|  |  | $x_{12}\leftrightarrow x_{9}\to y$ | -0.480 | -0.069 |  |  |  |  |
|  |  | $x_{12}\leftrightarrow x_{10}\to y$ | 4.164 | 0.000 |  |  |  |  |
|  |  | $x_{12}\leftrightarrow x_{11}\to y$ | -6.685 | -0.031 |  |  |  |  |
|  |  | $x_{12}\leftrightarrow x_{13}\to y$ | -0.941 | 0.141 |  |  |  |  |
| $\begin{matrix} x_{13} \\ \text{to} \\ y \end{matrix}$ | -2.117 | $x_{13}\leftrightarrow x_{1}\to y$ | -2.248 | 0.063 | 2.815 | 0.698 | -1.351 | 0.409 |
|  |  | $x_{13}\leftrightarrow x_{2}\to y$ | 2.337 | 0.752 |  |  |  |  |
|  |  | $x_{13}\leftrightarrow x_{3}\to y$ | 6.136 | 0.005 |  |  |  |  |
|  |  | $x_{13}\leftrightarrow x_{4}\to y$ | 4.366 | 0.017 |  |  |  |  |
|  |  | $x_{13}\leftrightarrow x_{5}\to y$ | -1.794 | -0.141 |  |  |  |  |
|  |  | $x_{13}\leftrightarrow x_{6}\to y$ | -1.314 | -0.281 |  |  |  |  |
|  |  | $x_{13}\leftrightarrow x_{7}\to y$ | -3.647 | 0.020 |  |  |  |  |
|  |  | $x_{13}\leftrightarrow x_{8}\to y$ | -2.131 | -0.239 |  |  |  |  |
|  |  | $x_{13}\leftrightarrow x_{9}\to y$ | -0.422 | -0.067 |  |  |  |  |
|  |  | $x_{13}\leftrightarrow x_{10}\to y$ | 4.692 | 0.000 |  |  |  |  |
|  |  | $x_{13}\leftrightarrow x_{11}\to y$ | -7.011 | -0.037 |  |  |  |  |
|  |  | $x_{13}\leftrightarrow x_{12}\to y$ | 3.850 | 0.141 |  |  |  |  |

| **1.1 Carbohydrate Metabolism** | | | | | | | | |
| --- | --- | --- | --- | --- | --- | --- | --- | --- |
| $\begin{matrix} x_{j} \\ \text{to} \\ y \end{matrix}$ | $b_{j}^{*}$ | $x_{j}\leftrightarrow x_{k}\to y$ | $r_{jk}b_{k}^{*}$ | $2b_{j}^{*}r_{jk}b_{k}^{*}$ | $\sum_{k\neq j} r_{jk}b_{k}^{*}$ | $r_{jy}$ | $\sum_{j\neq k} r_{kj}b_{j}^{*}$ | $R_{(j)}$ |
| $\begin{matrix} x_{1} \\ \text{to} \\ y \end{matrix}$ | 0.007 | $x_{1}\leftrightarrow x_{2}\to y$ | -0.148 | -0.002 | 0.908 | 0.914 | 0.068 | 0.012 |
|  |  | $x_{1}\leftrightarrow x_{3}\to y$ | -0.055 | -0.001 |  |  |  |  |
|  |  | $x_{1}\leftrightarrow x_{4}\to y$ | 0.470 | 0.006 |  |  |  |  |
|  |  | $x_{1}\leftrightarrow x_{5}\to y$ | -0.037 | 0.000 |  |  |  |  |
|  |  | $x_{1}\leftrightarrow x_{6}\to y$ | -0.099 | -0.001 |  |  |  |  |
|  |  | $x_{1}\leftrightarrow x_{7}\to y$ | 0.260 | 0.003 |  |  |  |  |
|  |  | $x_{1}\leftrightarrow x_{8}\to y$ | 0.194 | 0.003 |  |  |  |  |
|  |  | $x_{1}\leftrightarrow x_{9}\to y$ | 0.128 | 0.002 |  |  |  |  |
|  |  | $x_{1}\leftrightarrow x_{10}\to y$ | 0.306 | 0.004 |  |  |  |  |
|  |  | $x_{1}\leftrightarrow x_{11}\to y$ | 0.077 | 0.001 |  |  |  |  |
|  |  | $x_{1}\leftrightarrow x_{12}\to y$ | -0.625 | -0.008 |  |  |  |  |
|  |  | $x_{1}\leftrightarrow x_{13}\to y$ | 0.290 | 0.004 |  |  |  |  |
|  |  | $x_{1}\leftrightarrow x_{14}\to y$ | 0.147 | 0.002 |  |  |  |  |
| $\begin{matrix} x_{2} \\ \text{to} \\ y \end{matrix}$ | -0.174 | $x_{2}\leftrightarrow x_{1}\to y$ | 0.006 | -0.002 | 1.131 | 0.957 | -1.859 | -0.362 |
|  |  | $x_{2}\leftrightarrow x_{3}\to y$ | -0.063 | 0.022 |  |  |  |  |
|  |  | $x_{2}\leftrightarrow x_{4}\to y$ | 0.472 | -0.164 |  |  |  |  |
|  |  | $x_{2}\leftrightarrow x_{5}\to y$ | -0.047 | 0.016 |  |  |  |  |
|  |  | $x_{2}\leftrightarrow x_{6}\to y$ | -0.110 | 0.038 |  |  |  |  |
|  |  | $x_{2}\leftrightarrow x_{7}\to y$ | 0.254 | -0.088 |  |  |  |  |
|  |  | $x_{2}\leftrightarrow x_{8}\to y$ | 0.179 | -0.062 |  |  |  |  |
|  |  | $x_{2}\leftrightarrow x_{9}\to y$ | 0.129 | -0.045 |  |  |  |  |
|  |  | $x_{2}\leftrightarrow x_{10}\to y$ | 0.324 | -0.112 |  |  |  |  |
|  |  | $x_{2}\leftrightarrow x_{11}\to y$ | 0.106 | -0.037 |  |  |  |  |
|  |  | $x_{2}\leftrightarrow x_{12}\to y$ | -0.549 | 0.191 |  |  |  |  |
|  |  | $x_{2}\leftrightarrow x_{13}\to y$ | 0.315 | -0.109 |  |  |  |  |
|  |  | $x_{2}\leftrightarrow x_{14}\to y$ | 0.116 | -0.040 |  |  |  |  |
| $\begin{matrix} x_{3} \\ \text{to} \\ y \end{matrix}$ | -0.160 | $x_{3}\leftrightarrow x_{1}\to y$ | 0.002 | -0.001 | 0.579 | 0.419 | -0.870 | -0.160 |
|  |  | $x_{3}\leftrightarrow x_{2}\to y$ | -0.068 | 0.022 |  |  |  |  |
|  |  | $x_{3}\leftrightarrow x_{4}\to y$ | 0.215 | -0.069 |  |  |  |  |
|  |  | $x_{3}\leftrightarrow x_{5}\to y$ | -0.028 | 0.009 |  |  |  |  |
|  |  | $x_{3}\leftrightarrow x_{6}\to y$ | -0.043 | 0.014 |  |  |  |  |
|  |  | $x_{3}\leftrightarrow x_{7}\to y$ | 0.100 | -0.032 |  |  |  |  |
|  |  | $x_{3}\leftrightarrow x_{8}\to y$ | 0.120 | -0.038 |  |  |  |  |
|  |  | $x_{3}\leftrightarrow x_{9}\to y$ | 0.077 | -0.025 |  |  |  |  |
|  |  | $x_{3}\leftrightarrow x_{10}\to y$ | 0.096 | -0.031 |  |  |  |  |
|  |  | $x_{3}\leftrightarrow x_{11}\to y$ | 0.099 | -0.032 |  |  |  |  |
|  |  | $x_{3}\leftrightarrow x_{12}\to y$ | -0.169 | 0.054 |  |  |  |  |
|  |  | $x_{3}\leftrightarrow x_{13}\to y$ | 0.119 | -0.038 |  |  |  |  |
|  |  | $x_{3}\leftrightarrow x_{14}\to y$ | 0.059 | -0.019 |  |  |  |  |
| $\begin{matrix} x_{4} \\ \text{to} \\ y \end{matrix}$ | 0.526 | $x_{4}\leftrightarrow x_{1}\to y$ | 0.006 | 0.006 | 0.458 | 0.985 | 5.601 | 0.760 |
|  |  | $x_{4}\leftrightarrow x_{2}\to y$ | -0.156 | -0.164 |  |  |  |  |
|  |  | $x_{4}\leftrightarrow x_{3}\to y$ | -0.065 | -0.069 |  |  |  |  |
|  |  | $x_{4}\leftrightarrow x_{5}\to y$ | -0.036 | -0.038 |  |  |  |  |
|  |  | $x_{4}\leftrightarrow x_{6}\to y$ | -0.125 | -0.131 |  |  |  |  |
|  |  | $x_{4}\leftrightarrow x_{7}\to y$ | 0.274 | 0.289 |  |  |  |  |
|  |  | $x_{4}\leftrightarrow x_{8}\to y$ | 0.208 | 0.219 |  |  |  |  |
|  |  | $x_{4}\leftrightarrow x_{9}\to y$ | 0.130 | 0.137 |  |  |  |  |
|  |  | $x_{4}\leftrightarrow x_{10}\to y$ | 0.313 | 0.330 |  |  |  |  |
|  |  | $x_{4}\leftrightarrow x_{11}\to y$ | 0.085 | 0.090 |  |  |  |  |
|  |  | $x_{4}\leftrightarrow x_{12}\to y$ | -0.586 | -0.616 |  |  |  |  |
|  |  | $x_{4}\leftrightarrow x_{13}\to y$ | 0.316 | 0.333 |  |  |  |  |
|  |  | $x_{4}\leftrightarrow x_{14}\to y$ | 0.093 | 0.098 |  |  |  |  |
| $\begin{matrix} x_{5} \\ \text{to} \\ y \end{matrix}$ | -0.051 | $x_{5}\leftrightarrow x_{1}\to y$ | 0.005 | 0.000 | 0.855 | 0.804 | -0.487 | -0.085 |
|  |  | $x_{5}\leftrightarrow x_{2}\to y$ | -0.159 | 0.016 |  |  |  |  |
|  |  | $x_{5}\leftrightarrow x_{3}\to y$ | -0.088 | 0.009 |  |  |  |  |
|  |  | $x_{5}\leftrightarrow x_{4}\to y$ | 0.373 | -0.038 |  |  |  |  |
|  |  | $x_{5}\leftrightarrow x_{6}\to y$ | -0.083 | 0.008 |  |  |  |  |
|  |  | $x_{5}\leftrightarrow x_{7}\to y$ | 0.195 | -0.020 |  |  |  |  |
|  |  | $x_{5}\leftrightarrow x_{8}\to y$ | 0.146 | -0.015 |  |  |  |  |
|  |  | $x_{5}\leftrightarrow x_{9}\to y$ | 0.120 | -0.012 |  |  |  |  |
|  |  | $x_{5}\leftrightarrow x_{10}\to y$ | 0.280 | -0.029 |  |  |  |  |
|  |  | $x_{5}\leftrightarrow x_{11}\to y$ | 0.109 | -0.011 |  |  |  |  |
|  |  | $x_{5}\leftrightarrow x_{12}\to y$ | -0.433 | 0.044 |  |  |  |  |
|  |  | $x_{5}\leftrightarrow x_{13}\to y$ | 0.269 | -0.028 |  |  |  |  |
|  |  | $x_{5}\leftrightarrow x_{14}\to y$ | 0.122 | -0.012 |  |  |  |  |
| $\begin{matrix} x_{6} \\ \text{to} \\ y \end{matrix}$ | -0.129 | $x_{6}\leftrightarrow x_{1}\to y$ | 0.005 | -0.001 | 1.070 | 0.940 | -1.257 | -0.260 |
|  |  | $x_{6}\leftrightarrow x_{2}\to y$ | -0.148 | 0.038 |  |  |  |  |
|  |  | $x_{6}\leftrightarrow x_{3}\to y$ | -0.053 | 0.014 |  |  |  |  |
|  |  | $x_{6}\leftrightarrow x_{4}\to y$ | 0.509 | -0.131 |  |  |  |  |
|  |  | $x_{6}\leftrightarrow x_{5}\to y$ | -0.033 | 0.008 |  |  |  |  |
|  |  | $x_{6}\leftrightarrow x_{7}\to y$ | 0.256 | -0.066 |  |  |  |  |
|  |  | $x_{6}\leftrightarrow x_{8}\to y$ | 0.197 | -0.051 |  |  |  |  |
|  |  | $x_{6}\leftrightarrow x_{9}\to y$ | 0.121 | -0.031 |  |  |  |  |
|  |  | $x_{6}\leftrightarrow x_{10}\to y$ | 0.291 | -0.075 |  |  |  |  |
|  |  | $x_{6}\leftrightarrow x_{11}\to y$ | 0.077 | -0.020 |  |  |  |  |
|  |  | $x_{6}\leftrightarrow x_{12}\to y$ | -0.518 | 0.134 |  |  |  |  |
|  |  | $x_{6}\leftrightarrow x_{13}\to y$ | 0.300 | -0.078 |  |  |  |  |
|  |  | $x_{6}\leftrightarrow x_{14}\to y$ | 0.066 | -0.017 |  |  |  |  |
| $\begin{matrix} x_{7} \\ \text{to} \\ y \end{matrix}$ | 0.283 | $x_{7}\leftrightarrow x_{1}\to y$ | 0.006 | 0.003 | 0.687 | 0.971 | 2.999 | 0.470 |
|  |  | $x_{7}\leftrightarrow x_{2}\to y$ | -0.156 | -0.088 |  |  |  |  |
|  |  | $x_{7}\leftrightarrow x_{3}\to y$ | -0.057 | -0.032 |  |  |  |  |
|  |  | $x_{7}\leftrightarrow x_{4}\to y$ | 0.510 | 0.289 |  |  |  |  |
|  |  | $x_{7}\leftrightarrow x_{5}\to y$ | -0.035 | -0.020 |  |  |  |  |
|  |  | $x_{7}\leftrightarrow x_{6}\to y$ | -0.117 | -0.066 |  |  |  |  |
|  |  | $x_{7}\leftrightarrow x_{8}\to y$ | 0.204 | 0.116 |  |  |  |  |
|  |  | $x_{7}\leftrightarrow x_{9}\to y$ | 0.127 | 0.072 |  |  |  |  |
|  |  | $x_{7}\leftrightarrow x_{10}\to y$ | 0.314 | 0.178 |  |  |  |  |
|  |  | $x_{7}\leftrightarrow x_{11}\to y$ | 0.090 | 0.051 |  |  |  |  |
|  |  | $x_{7}\leftrightarrow x_{12}\to y$ | -0.610 | -0.346 |  |  |  |  |
|  |  | $x_{7}\leftrightarrow x_{13}\to y$ | 0.308 | 0.175 |  |  |  |  |
|  |  | $x_{7}\leftrightarrow x_{14}\to y$ | 0.103 | 0.058 |  |  |  |  |
| $\begin{matrix} x_{8} \\ \text{to} \\ y \end{matrix}$ | 0.223 | $x_{8}\leftrightarrow x_{1}\to y$ | 0.006 | 0.003 | 0.694 | 0.918 | 2.290 | 0.360 |
|  |  | $x_{8}\leftrightarrow x_{2}\to y$ | -0.139 | -0.062 |  |  |  |  |
|  |  | $x_{8}\leftrightarrow x_{3}\to y$ | -0.086 | -0.038 |  |  |  |  |
|  |  | $x_{8}\leftrightarrow x_{4}\to y$ | 0.491 | 0.219 |  |  |  |  |
|  |  | $x_{8}\leftrightarrow x_{5}\to y$ | -0.034 | -0.015 |  |  |  |  |
|  |  | $x_{8}\leftrightarrow x_{6}\to y$ | -0.114 | -0.051 |  |  |  |  |
|  |  | $x_{8}\leftrightarrow x_{7}\to y$ | 0.259 | 0.116 |  |  |  |  |
|  |  | $x_{8}\leftrightarrow x_{9}\to y$ | 0.134 | 0.060 |  |  |  |  |
|  |  | $x_{8}\leftrightarrow x_{10}\to y$ | 0.274 | 0.122 |  |  |  |  |
|  |  | $x_{8}\leftrightarrow x_{11}\to y$ | 0.081 | 0.036 |  |  |  |  |
|  |  | $x_{8}\leftrightarrow x_{12}\to y$ | -0.560 | -0.250 |  |  |  |  |
|  |  | $x_{8}\leftrightarrow x_{13}\to y$ | 0.273 | 0.122 |  |  |  |  |
|  |  | $x_{8}\leftrightarrow x_{14}\to y$ | 0.110 | 0.049 |  |  |  |  |
| $\begin{matrix} x_{9} \\ \text{to} \\ y \end{matrix}$ | 0.141 | $x_{9}\leftrightarrow x_{1}\to y$ | 0.006 | 0.002 | 0.808 | 0.949 | 1.541 | 0.248 |
|  |  | $x_{9}\leftrightarrow x_{2}\to y$ | -0.158 | -0.045 |  |  |  |  |
|  |  | $x_{9}\leftrightarrow x_{3}\to y$ | -0.087 | -0.025 |  |  |  |  |
|  |  | $x_{9}\leftrightarrow x_{4}\to y$ | 0.485 | 0.137 |  |  |  |  |
|  |  | $x_{9}\leftrightarrow x_{5}\to y$ | -0.043 | -0.012 |  |  |  |  |
|  |  | $x_{9}\leftrightarrow x_{6}\to y$ | -0.110 | -0.031 |  |  |  |  |
|  |  | $x_{9}\leftrightarrow x_{7}\to y$ | 0.254 | 0.072 |  |  |  |  |
|  |  | $x_{9}\leftrightarrow x_{8}\to y$ | 0.212 | 0.060 |  |  |  |  |
|  |  | $x_{9}\leftrightarrow x_{10}\to y$ | 0.304 | 0.086 |  |  |  |  |
|  |  | $x_{9}\leftrightarrow x_{11}\to y$ | 0.092 | 0.026 |  |  |  |  |
|  |  | $x_{9}\leftrightarrow x_{12}\to y$ | -0.570 | -0.161 |  |  |  |  |
|  |  | $x_{9}\leftrightarrow x_{13}\to y$ | 0.295 | 0.083 |  |  |  |  |
|  |  | $x_{9}\leftrightarrow x_{14}\to y$ | 0.130 | 0.037 |  |  |  |  |
| $\begin{matrix} x_{10} \\ \text{to} \\ y \end{matrix}$ | 0.335 | $x_{10}\leftrightarrow x_{1}\to y$ | 0.006 | 0.004 | 0.633 | 0.968 | 3.564 | 0.537 |
|  |  | $x_{10}\leftrightarrow x_{2}\to y$ | -0.168 | -0.112 |  |  |  |  |
|  |  | $x_{10}\leftrightarrow x_{3}\to y$ | -0.046 | -0.031 |  |  |  |  |
|  |  | $x_{10}\leftrightarrow x_{4}\to y$ | 0.492 | 0.330 |  |  |  |  |
|  |  | $x_{10}\leftrightarrow x_{5}\to y$ | -0.043 | -0.029 |  |  |  |  |
|  |  | $x_{10}\leftrightarrow x_{6}\to y$ | -0.112 | -0.075 |  |  |  |  |
|  |  | $x_{10}\leftrightarrow x_{7}\to y$ | 0.265 | 0.178 |  |  |  |  |
|  |  | $x_{10}\leftrightarrow x_{8}\to y$ | 0.182 | 0.122 |  |  |  |  |
|  |  | $x_{10}\leftrightarrow x_{9}\to y$ | 0.128 | 0.086 |  |  |  |  |
|  |  | $x_{10}\leftrightarrow x_{11}\to y$ | 0.088 | 0.059 |  |  |  |  |
|  |  | $x_{10}\leftrightarrow x_{12}\to y$ | -0.595 | -0.399 |  |  |  |  |
|  |  | $x_{10}\leftrightarrow x_{13}\to y$ | 0.324 | 0.217 |  |  |  |  |
|  |  | $x_{10}\leftrightarrow x_{14}\to y$ | 0.111 | 0.074 |  |  |  |  |
| $\begin{matrix} x_{11} \\ \text{to} \\ y \end{matrix}$ | 0.137 | $x_{11}\leftrightarrow x_{1}\to y$ | 0.004 | 0.001 | 0.563 | 0.700 | 1.137 | 0.172 |
|  |  | $x_{11}\leftrightarrow x_{2}\to y$ | -0.135 | -0.037 |  |  |  |  |
|  |  | $x_{11}\leftrightarrow x_{3}\to y$ | -0.117 | -0.032 |  |  |  |  |
|  |  | $x_{11}\leftrightarrow x_{4}\to y$ | 0.328 | 0.090 |  |  |  |  |
|  |  | $x_{11}\leftrightarrow x_{5}\to y$ | -0.041 | -0.011 |  |  |  |  |
|  |  | $x_{11}\leftrightarrow x_{6}\to y$ | -0.073 | -0.020 |  |  |  |  |
|  |  | $x_{11}\leftrightarrow x_{7}\to y$ | 0.186 | 0.051 |  |  |  |  |
|  |  | $x_{11}\leftrightarrow x_{8}\to y$ | 0.133 | 0.036 |  |  |  |  |
|  |  | $x_{11}\leftrightarrow x_{9}\to y$ | 0.095 | 0.026 |  |  |  |  |
|  |  | $x_{11}\leftrightarrow x_{10}\to y$ | 0.216 | 0.059 |  |  |  |  |
|  |  | $x_{11}\leftrightarrow x_{12}\to y$ | -0.345 | -0.094 |  |  |  |  |
|  |  | $x_{11}\leftrightarrow x_{13}\to y$ | 0.224 | 0.061 |  |  |  |  |
|  |  | $x_{11}\leftrightarrow x_{14}\to y$ | 0.088 | 0.024 |  |  |  |  |
| $\begin{matrix} x_{12} \\ \text{to} \\ y \end{matrix}$ | -0.635 | $x_{12}\leftrightarrow x_{1}\to y$ | 0.006 | -0.008 | 1.570 | 0.934 | -6.585 | -1.591 |
|  |  | $x_{12}\leftrightarrow x_{2}\to y$ | -0.150 | 0.191 |  |  |  |  |
|  |  | $x_{12}\leftrightarrow x_{3}\to y$ | -0.043 | 0.054 |  |  |  |  |
|  |  | $x_{12}\leftrightarrow x_{4}\to y$ | 0.485 | -0.616 |  |  |  |  |
|  |  | $x_{12}\leftrightarrow x_{5}\to y$ | -0.035 | 0.044 |  |  |  |  |
|  |  | $x_{12}\leftrightarrow x_{6}\to y$ | -0.105 | 0.134 |  |  |  |  |
|  |  | $x_{12}\leftrightarrow x_{7}\to y$ | 0.272 | -0.346 |  |  |  |  |
|  |  | $x_{12}\leftrightarrow x_{8}\to y$ | 0.197 | -0.250 |  |  |  |  |
|  |  | $x_{12}\leftrightarrow x_{9}\to y$ | 0.127 | -0.161 |  |  |  |  |
|  |  | $x_{12}\leftrightarrow x_{10}\to y$ | 0.314 | -0.399 |  |  |  |  |
|  |  | $x_{12}\leftrightarrow x_{11}\to y$ | 0.074 | -0.094 |  |  |  |  |
|  |  | $x_{12}\leftrightarrow x_{13}\to y$ | 0.298 | -0.378 |  |  |  |  |
|  |  | $x_{12}\leftrightarrow x_{14}\to y$ | 0.130 | -0.165 |  |  |  |  |
| $\begin{matrix} x_{13} \\ \text{to} \\ y \end{matrix}$ | 0.329 | $x_{13}\leftrightarrow x_{1}\to y$ | 0.006 | 0.004 | 0.650 | 0.979 | 3.504 | 0.535 |
|  |  | $x_{13}\leftrightarrow x_{2}\to y$ | -0.166 | -0.109 |  |  |  |  |
|  |  | $x_{13}\leftrightarrow x_{3}\to y$ | -0.058 | -0.038 |  |  |  |  |
|  |  | $x_{13}\leftrightarrow x_{4}\to y$ | 0.506 | 0.333 |  |  |  |  |
|  |  | $x_{13}\leftrightarrow x_{5}\to y$ | -0.042 | -0.028 |  |  |  |  |
|  |  | $x_{13}\leftrightarrow x_{6}\to y$ | -0.118 | -0.078 |  |  |  |  |
|  |  | $x_{13}\leftrightarrow x_{7}\to y$ | 0.266 | 0.175 |  |  |  |  |
|  |  | $x_{13}\leftrightarrow x_{8}\to y$ | 0.185 | 0.122 |  |  |  |  |
|  |  | $x_{13}\leftrightarrow x_{9}\to y$ | 0.127 | 0.083 |  |  |  |  |
|  |  | $x_{13}\leftrightarrow x_{10}\to y$ | 0.330 | 0.217 |  |  |  |  |
|  |  | $x_{13}\leftrightarrow x_{11}\to y$ | 0.093 | 0.061 |  |  |  |  |
|  |  | $x_{13}\leftrightarrow x_{12}\to y$ | -0.576 | -0.378 |  |  |  |  |
|  |  | $x_{13}\leftrightarrow x_{14}\to y$ | 0.097 | 0.064 |  |  |  |  |
| $\begin{matrix} x_{14} \\ \text{to} \\ y \end{matrix}$ | 0.184 | $x_{14}\leftrightarrow x_{1}\to y$ | 0.005 | 0.002 | 0.412 | 0.596 | 1.371 | 0.186 |
|  |  | $x_{14}\leftrightarrow x_{2}\to y$ | -0.109 | -0.040 |  |  |  |  |
|  |  | $x_{14}\leftrightarrow x_{3}\to y$ | -0.052 | -0.019 |  |  |  |  |
|  |  | $x_{14}\leftrightarrow x_{4}\to y$ | 0.265 | 0.098 |  |  |  |  |
|  |  | $x_{14}\leftrightarrow x_{5}\to y$ | -0.034 | -0.012 |  |  |  |  |
|  |  | $x_{14}\leftrightarrow x_{6}\to y$ | -0.046 | -0.017 |  |  |  |  |
|  |  | $x_{14}\leftrightarrow x_{7}\to y$ | 0.158 | 0.058 |  |  |  |  |
|  |  | $x_{14}\leftrightarrow x_{8}\to y$ | 0.133 | 0.049 |  |  |  |  |
|  |  | $x_{14}\leftrightarrow x_{9}\to y$ | 0.099 | 0.037 |  |  |  |  |
|  |  | $x_{14}\leftrightarrow x_{10}\to y$ | 0.202 | 0.074 |  |  |  |  |
|  |  | $x_{14}\leftrightarrow x_{11}\to y$ | 0.065 | 0.024 |  |  |  |  |
|  |  | $x_{14}\leftrightarrow x_{12}\to y$ | -0.448 | -0.165 |  |  |  |  |
|  |  | $x_{14}\leftrightarrow x_{13}\to y$ | 0.173 | 0.064 |  |  |  |  |

| **1.8 Metabolism of Cofactors and Vitamins** | | | | | | | | |
| --- | --- | --- | --- | --- | --- | --- | --- | --- |
| $\begin{matrix} x_{j} \\ \text{to} \\ y \end{matrix}$ | $b_{j}^{*}$ | $x_{j}\leftrightarrow x_{k}\to y$ | $r_{jk}b_{k}^{*}$ | $2b_{j}^{*}r_{jk}b_{k}^{*}$ | $\sum_{k\neq j} r_{jk}b_{k}^{*}$ | $r_{jy}$ | $\sum_{j\neq k} r_{kj}b_{j}^{*}$ | $R_{(j)}$ |
| $\begin{matrix} x_{1} \\ \text{to} \\ y \end{matrix}$ | 0.503 | $x_{1}\leftrightarrow x_{2}\to y$ | -0.520 | -0.284 | -0.18 | 0.323 | 0.796 | 0.103 |
|  |  | $x_{1}\leftrightarrow x_{3}\to y$ | -1.492 | -0.822 |  |  |  |  |
|  |  | $x_{1}\leftrightarrow x_{4}\to y$ | 0.014 | 0.005 |  |  |  |  |
|  |  | $x_{1}\leftrightarrow x_{5}\to y$ | 1.199 | 0.746 |  |  |  |  |
|  |  | $x_{1}\leftrightarrow x_{6}\to y$ | -0.033 | -0.023 |  |  |  |  |
|  |  | $x_{1}\leftrightarrow x_{7}\to y$ | 0.750 | 0.468 |  |  |  |  |
|  |  | $x_{1}\leftrightarrow x_{8}\to y$ | -0.098 | -0.066 |  |  |  |  |
| $\begin{matrix} x_{2} \\ \text{to} \\ y \end{matrix}$ | -1.185 | $x_{2}\leftrightarrow x_{1}\to y$ | 0.221 | -0.284 | 1.930 | 0.745 | -3.293 | -3.031 |
|  |  | $x_{2}\leftrightarrow x_{3}\to y$ | -0.265 | 0.595 |  |  |  |  |
|  |  | $x_{2}\leftrightarrow x_{4}\to y$ | 0.228 | -0.353 |  |  |  |  |
|  |  | $x_{2}\leftrightarrow x_{5}\to y$ | 1.975 | -5.007 |  |  |  |  |
|  |  | $x_{2}\leftrightarrow x_{6}\to y$ | -0.366 | 1.026 |  |  |  |  |
|  |  | $x_{2}\leftrightarrow x_{7}\to y$ | 0.231 | -0.587 |  |  |  |  |
|  |  | $x_{2}\leftrightarrow x_{8}\to y$ | -0.094 | 0.260 |  |  |  |  |
| $\begin{matrix} x_{3} \\ \text{to} \\ y \end{matrix}$ | -1.698 | $x_{3}\leftrightarrow x_{1}\to y$ | 0.442 | -0.822 | 1.889 | 0.191 | -1.998 | -3.395 |
|  |  | $x_{3}\leftrightarrow x_{2}\to y$ | -0.185 | 0.595 |  |  |  |  |
|  |  | $x_{3}\leftrightarrow x_{4}\to y$ | 0.019 | -0.042 |  |  |  |  |
|  |  | $x_{3}\leftrightarrow x_{5}\to y$ | 0.964 | -3.535 |  |  |  |  |
|  |  | $x_{3}\leftrightarrow x_{6}\to y$ | -0.028 | 0.113 |  |  |  |  |
|  |  | $x_{3}\leftrightarrow x_{7}\to y$ | 0.766 | -2.817 |  |  |  |  |
|  |  | $x_{3}\leftrightarrow x_{8}\to y$ | -0.089 | 0.355 |  |  |  |  |
| $\begin{matrix} x_{4} \\ \text{to} \\ y \end{matrix}$ | 0.316 | $x_{4}\leftrightarrow x_{1}\to y$ | 0.023 | 0.005 | 0.506 | 0.822 | 0.825 | 0.304 |
|  |  | $x_{4}\leftrightarrow x_{2}\to y$ | -0.855 | -0.353 |  |  |  |  |
|  |  | $x_{4}\leftrightarrow x_{3}\to y$ | -0.101 | -0.042 |  |  |  |  |
|  |  | $x_{4}\leftrightarrow x_{5}\to y$ | 2.021 | 0.949 |  |  |  |  |
|  |  | $x_{4}\leftrightarrow x_{6}\to y$ | -0.512 | -0.266 |  |  |  |  |
|  |  | $x_{4}\leftrightarrow x_{7}\to y$ | -0.024 | -0.011 |  |  |  |  |
|  |  | $x_{4}\leftrightarrow x_{8}\to y$ | -0.046 | -0.024 |  |  |  |  |
| $\begin{matrix} x_{5} \\ \text{to} \\ y \end{matrix}$ | 2.581 | $x_{5}\leftrightarrow x_{1}\to y$ | 0.234 | 0.746 | -1.695 | 0.886 | 8.528 | -3.065 |
|  |  | $x_{5}\leftrightarrow x_{2}\to y$ | -0.906 | -5.007 |  |  |  |  |
|  |  | $x_{5}\leftrightarrow x_{3}\to y$ | -0.634 | -3.535 |  |  |  |  |
|  |  | $x_{5}\leftrightarrow x_{4}\to y$ | 0.247 | 0.949 |  |  |  |  |
|  |  | $x_{5}\leftrightarrow x_{6}\to y$ | -0.528 | -3.667 |  |  |  |  |
|  |  | $x_{5}\leftrightarrow x_{7}\to y$ | -0.129 | -0.817 |  |  |  |  |
|  |  | $x_{5}\leftrightarrow x_{8}\to y$ | 0.022 | 0.152 |  |  |  |  |
| $\begin{matrix} x_{6} \\ \text{to} \\ y \end{matrix}$ | -0.600 | $x_{6}\leftrightarrow x_{1}\to y$ | 0.028 | -0.023 | 1.438 | 0.839 | -1.515 | -1.760 |
|  |  | $x_{6}\leftrightarrow x_{2}\to y$ | -0.724 | 1.026 |  |  |  |  |
|  |  | $x_{6}\leftrightarrow x_{3}\to y$ | -0.079 | 0.113 |  |  |  |  |
|  |  | $x_{6}\leftrightarrow x_{4}\to y$ | 0.270 | -0.266 |  |  |  |  |
|  |  | $x_{6}\leftrightarrow x_{5}\to y$ | 2.273 | -3.667 |  |  |  |  |
|  |  | $x_{6}\leftrightarrow x_{7}\to y$ | -0.415 | 0.672 |  |  |  |  |
|  |  | $x_{6}\leftrightarrow x_{8}\to y$ | 0.085 | -0.150 |  |  |  |  |
| $\begin{matrix} x_{7} \\ \text{to} \\ y \end{matrix}$ | -1.308 | $x_{7}\leftrightarrow x_{1}\to y$ | -0.288 | 0.468 | 1.273 | -0.035 | 2.234 | -1.999 |
|  |  | $x_{7}\leftrightarrow x_{2}\to y$ | 0.209 | -0.587 |  |  |  |  |
|  |  | $x_{7}\leftrightarrow x_{3}\to y$ | 0.994 | -2.817 |  |  |  |  |
|  |  | $x_{7}\leftrightarrow x_{4}\to y$ | 0.006 | -0.011 |  |  |  |  |
|  |  | $x_{7}\leftrightarrow x_{5}\to y$ | 0.255 | -0.817 |  |  |  |  |
|  |  | $x_{7}\leftrightarrow x_{6}\to y$ | -0.190 | 0.672 |  |  |  |  |
|  |  | $x_{7}\leftrightarrow x_{8}\to y$ | 0.288 | -1.006 |  |  |  |  |
| $\begin{matrix} x_{8} \\ \text{to} \\ y \end{matrix}$ | -0.356 | $x_{8}\leftrightarrow x_{1}\to y$ | 0.138 | -0.066 | 0.483 | 0.127 | 0.069 | -0.294 |
|  |  | $x_{8}\leftrightarrow x_{2}\to y$ | -0.312 | 0.260 |  |  |  |  |
|  |  | $x_{8}\leftrightarrow x_{3}\to y$ | -0.422 | 0.355 |  |  |  |  |
|  |  | $x_{8}\leftrightarrow x_{4}\to y$ | 0.041 | -0.024 |  |  |  |  |
|  |  | $x_{8}\leftrightarrow x_{5}\to y$ | -0.161 | 0.152 |  |  |  |  |
|  |  | $x_{8}\leftrightarrow x_{6}\to y$ | 0.143 | -0.150 |  |  |  |  |
|  |  | $x_{8}\leftrightarrow x_{7}\to y$ | 1.056 | -1.006 |  |  |  |  |

| **1.4 Nucleotide Metabolism** | | | | | | | | |
| --- | --- | --- | --- | --- | --- | --- | --- | --- |
| $\begin{matrix} x_{j} \\ \text{to} \\ y \end{matrix}$ | $b_{j}^{*}$ | $x_{j}\leftrightarrow x_{k}\to y$ | $r_{jk}b_{k}^{*}$ | $2b_{j}^{*}r_{jk}b_{k}^{*}$ | $\sum_{k\neq j} r_{jk}b_{k}^{*}$ | $r_{jy}$ | $\sum_{j\neq k} r_{kj}b_{j}^{*}$ | $R_{(j)}$ |
| $\begin{matrix} x_{1} \\ \text{to} \\ y \end{matrix}$ | 0.610 | $x_{1}\leftrightarrow x_{2}\to y$ | 0.379 | 0.462 | 0.379 | 0.988 | 0.566 | 0.834 |
| $\begin{matrix} x_{2} \\ \text{to} \\ y \end{matrix}$ | 0.408 | $x_{2}\leftrightarrow x_{1}\to y$ | 0.566 | 0.462 | 0.566 | 0.974 | 0.379 | 0.628 |

| **1.2 Energy Metabolism** | | | | | | | | |
| --- | --- | --- | --- | --- | --- | --- | --- | --- |
| $\begin{matrix} x_{j} \\ \text{to} \\ y \end{matrix}$ | $b_{j}^{*}$ | $x_{j}\leftrightarrow x_{k}\to y$ | $r_{jk}b_{k}^{*}$ | $2b_{j}^{*}r_{jk}b_{k}^{*}$ | $\sum_{k\neq j} r_{jk}b_{k}^{*}$ | $r_{jy}$ | $\sum_{j\neq k} r_{kj}b_{j}^{*}$ | $R_{(j)}$ |
| $\begin{matrix} x_{1} \\ \text{to} \\ y \end{matrix}$ | 0.206 | $x_{1}\leftrightarrow x_{2}\to y$ | 0.135 | 0.055 | 0.417 | 0.622 | 0.187 | 0.214 |
|  |  | $x_{1}\leftrightarrow x_{3}\to y$ | 0.282 | 0.116 |  |  |  |  |
| $\begin{matrix} x_{2} \\ \text{to} \\ y \end{matrix}$ | 0.557 | $x_{2}\leftrightarrow x_{1}\to y$ | 0.050 | 0.055 | 0.193 | 0.750 | 0.324 | 0.526 |
|  |  | $x_{2}\leftrightarrow x_{3}\to y$ | 0.143 | 0.160 |  |  |  |  |
| $\begin{matrix} x_{3} \\ \text{to} \\ y \end{matrix}$ | 0.421 | $x_{3}\leftrightarrow x_{1}\to y$ | 0.138 | 0.116 | 0.327 | 0.749 | 0.426 | 0.453 |
|  |  | $x_{3}\leftrightarrow x_{2}\to y$ | 0.190 | 0.160 |  |  |  |  |

| **1.11 Xenobiotics Biodegradation and Metabolism** | | | | | | | | |
| --- | --- | --- | --- | --- | --- | --- | --- | --- |
| $\begin{matrix} x_{j} \\ \text{to} \\ y \end{matrix}$ | $b_{j}^{*}$ | $x_{j}\leftrightarrow x_{k}\to y$ | $r_{jk}b_{k}^{*}$ | $2b_{j}^{*}r_{jk}b_{k}^{*}$ | $\sum_{k\neq j} r_{jk}b_{k}^{*}$ | $r_{jy}$ | $\sum_{j\neq k} r_{kj}b_{j}^{*}$ | $R_{(j)}$ |
| $\begin{matrix} x_{1} \\ \text{to} \\ y \end{matrix}$ | 0.251 | $x_{1}\leftrightarrow x_{2}\to y$ | 0.355 | 0.178 | 0.590 | 0.841 | 0.380 | 0.360 |
|  |  | $x_{1}\leftrightarrow x_{3}\to y$ | 0.235 | 0.118 |  |  |  |  |
| $\begin{matrix} x_{2} \\ \text{to} \\ y \end{matrix}$ | 0.565 | $x_{2}\leftrightarrow x_{1}\to y$ | 0.158 | 0.178 | 0.379 | 0.944 | 0.823 | 0.747 |
|  |  | $x_{2}\leftrightarrow x_{3}\to y$ | 0.221 | 0.250 |  |  |  |  |
| $\begin{matrix} x_{3} \\ \text{to} \\ y \end{matrix}$ | 0.267 | $x_{3}\leftrightarrow x_{1}\to y$ | 0.222 | 0.118 | 0.690 | 0.957 | 0.457 | 0.440 |
|  |  | $x_{3}\leftrightarrow x_{2}\to y$ | 0.469 | 0.250 |  |  |  |  |

| **3.3 Signaling Molecules and Interaction** | | | | | | | | |
| --- | --- | --- | --- | --- | --- | --- | --- | --- |
| $\begin{matrix} x_{j} \\ \text{to} \\ y \end{matrix}$ | $b_{j}^{*}$ | $x_{j}\leftrightarrow x_{k}\to y$ | $r_{jk}b_{k}^{*}$ | $2b_{j}^{*}r_{jk}b_{k}^{*}$ | $\sum_{k\neq j} r_{jk}b_{k}^{*}$ | $r_{jy}$ | $\sum_{j\neq k} r_{kj}b_{j}^{*}$ | $R_{(j)}$ |
| $\begin{matrix} x_{1} \\ \text{to} \\ y \end{matrix}$ | 0.396 | $x_{1}\leftrightarrow x_{2}\to y$ | 0.281 | 0.222 | 0.586 | 0.982 | 0.725 | 0.621 |
|  |  | $x_{1}\leftrightarrow x_{3}\to y$ | 0.305 | 0.241 |  |  |  |  |
| $\begin{matrix} x_{2} \\ \text{to} \\ y \end{matrix}$ | 0.293 | $x_{2}\leftrightarrow x_{1}\to y$ | 0.379 | 0.222 | 0.672 | 0.965 | 0.527 | 0.480 |
|  |  | $x_{2}\leftrightarrow x_{3}\to y$ | 0.293 | 0.172 |  |  |  |  |
| $\begin{matrix} x_{3} \\ \text{to} \\ y \end{matrix}$ | 0.349 | $x_{3}\leftrightarrow x_{1}\to y$ | 0.346 | 0.241 | 0.592 | 0.941 | 0.598 | 0.535 |
|  |  | $x_{3}\leftrightarrow x_{2}\to y$ | 0.246 | 0.172 |  |  |  |  |

| **1.6 Metabolism of Other Amino Acids** | | | | | | | | |
| --- | --- | --- | --- | --- | --- | --- | --- | --- |
| $\begin{matrix} x_{j} \\ \text{to} \\ y \end{matrix}$ | $b_{j}^{*}$ | $x_{j}\leftrightarrow x_{k}\to y$ | $r_{jk}b_{k}^{*}$ | $2b_{j}^{*}r_{jk}b_{k}^{*}$ | $\sum_{k\neq j} r_{jk}b_{k}^{*}$ | $r_{jy}$ | $\sum_{j\neq k} r_{kj}b_{j}^{*}$ | $R_{(j)}$ |
| $\begin{matrix} x_{1} \\ \text{to} \\ y \end{matrix}$ | 0.096 | $x_{1}\leftrightarrow x_{2}\to y$ | 0.537 | 0.103 | 0.738 | 0.833 | 0.209 | 0.150 |
|  |  | $x_{1}\leftrightarrow x_{3}\to y$ | -0.029 | -0.006 |  |  |  |  |
|  |  | $x_{1}\leftrightarrow x_{4}\to y$ | 0.230 | 0.044 |  |  |  |  |
| $\begin{matrix} x_{2} \\ \text{to} \\ y \end{matrix}$ | 0.631 | $x_{2}\leftrightarrow x_{1}\to y$ | 0.081 | 0.103 | 0.345 | 0.976 | 1.596 | 0.834 |
|  |  | $x_{2}\leftrightarrow x_{3}\to y$ | -0.035 | -0.045 |  |  |  |  |
|  |  | $x_{2}\leftrightarrow x_{4}\to y$ | 0.299 | 0.378 |  |  |  |  |
| $\begin{matrix} x_{3} \\ \text{to} \\ y \end{matrix}$ | -0.041 | $x_{3}\leftrightarrow x_{1}\to y$ | 0.068 | -0.006 | 0.873 | 0.832 | -0.093 | -0.070 |
|  |  | $x_{3}\leftrightarrow x_{2}\to y$ | 0.546 | -0.045 |  |  |  |  |
|  |  | $x_{3}\leftrightarrow x_{4}\to y$ | 0.259 | -0.021 |  |  |  |  |
| $\begin{matrix} x_{4} \\ \text{to} \\ y \end{matrix}$ | 0.368 | $x_{4}\leftrightarrow x_{1}\to y$ | 0.060 | 0.044 | 0.544 | 0.912 | 0.789 | 0.536 |
|  |  | $x_{4}\leftrightarrow x_{2}\to y$ | 0.513 | 0.378 |  |  |  |  |
|  |  | $x_{4}\leftrightarrow x_{3}\to y$ | -0.029 | -0.021 |  |  |  |  |
