# Supplementary material for: A decision analysis model for KEGG pathway analysis
Source: BMC Bioinformatics. 2016 Oct 6;17:407. doi: 10.1186/s12859-016-1285-1 (PMC5053338; doi:10.1186/s12859-016-1285-1)
Supplement: Additional file 10: S1. — This file provides the code for the decision analysis model in Matlab (R2008a, version 7.6.0.324). (DOCX 11 kb) [file 12859_2016_1285_MOESM1_ESM.docx]

**S1:** The code of decision analysis in Matlab (R2008a, version 7.6.0.324)

% % producing the simulated data, which satisfy the assumption of regression analysis %%

X=rand(4,4)*100;

e=normrnd(0,20,[4,1]);

Y=X*[1.3,1.6,2.1,-1.8]'+e

% % solving the path coefficient % %

%BJ: the solved path coefficient; Rxy: the correlation matrix of x and y; R: the correlation matrix of x. %

X=X'

Y= Y'

[m,n]=size(X);

R=corrcoef(X);

Rxy=corr(X,Y);

BJ = linsolve(R,Rxy); %BJ=R\Rxy; %BJ=inv(R)*Rxy;

abs(R*BJ-Rxy)./Rxy;

%% solving the decision coefficient (DC) value and subdividing the DC value %%

% RJ: decision coefficient (DC); RJper: decision percentage (*dp*); RBJ: the decomposed matrix of RJ, including direct and indirect determination factor %

RJ=2*Rxy.*BJ-BJ.^2;

RJper=abs(RJ)./sum(abs(RJ));

RBJ=2*R.*repmat(BJ',size(R,1),1).*repmat(BJ,1,size(R,2));
for i=1:size(R,1)
 RBJ(i,i)=RBJ(i,i)/2;
end

%% solving the coefficient of determination (CD) and subdividing the CD %%

% Rsqr: the coefficient of determination (CD); Rper: the decomposed CD, including direct and indirect CD %

Rsqr=sum(sum(tril(RBJ,0)));

Rper=[sum(diag(RBJ)),sum(sum(abs(tril(RBJ,-1))))]/sum(sum(abs(tril(RBJ,0))));
